# Supplementary material for: Trust in scientists and their role in society across 68 countries
Source: Nat Hum Behav. 2025 Jan 20;9(4):713–30. doi: 10.1038/s41562-024-02090-5 (PMC7617525; doi:10.1038/s41562-024-02090-5)
Supplement: Supplementary file 1 — Supplementary Figs. 1–31, Tables 1–22, Methods and Limitations. [file 41562_2024_2090_MOESM1_ESM.pdf]

nature human behaviour

Supplementary information https://doi.org/10.1038/s41562-024-02090-5

Trust in scientists and their role in society across 68 countries

In the format provided by the authors and unedited

Table of Content

Results 5

Fig. S1a. Directed acyclic graph (DAG) underlying results presented in Fig. 1 5

Fig. S1b. Alternative DAG proposed by an anonymous reviewer 6

Fig. S2. Valid sample sizes across countries. 7

Table S1. Weighted grand mean, median, SD, and SE for the 12-item index measuring trust in scientists, the four 3-item mean indices measuring the four dimensions, the 12 individual items, as well as the 1-item measures for confidence in scientists and trust in the scientific method. 8

Fig. S3. Weighted response probabilities for single trust in scientists scale items. 9

Table S2. Weighted linear multilevel regression testing the association of trust in scientists with demographic characteristics, attitudes to science, and country-level indicators (random intercepts across countries) 10

Fig. S4. Standardised estimates of weighted blockwise multilevel regression model testing the association of perceived competence with demographic characteristics, ideological views, attitudes, and country-level indicators (random intercepts across countries) 12

Fig. S5. Standardised estimates of weighted blockwise multilevel regression model testing the association of perceived integrity with demographic characteristics, ideological views, attitudes, and country-level indicators (random intercepts across countries) 13

Fig. S6. Standardised estimates of weighted blockwise multilevel regression model testing the association of perceived benevolence with demographic characteristics, ideological views, attitudes, and country-level indicators (random intercepts across countries) 14

Fig. S7. Standardised estimates of weighted blockwise multilevel regression model testing the association of perceived openness with demographic characteristics, ideological views, attitudes, and country-level indicators (random intercepts across countries) 15

Fig. S8. Random effects of weighted regression model explaining the relationship of education (tertiary) and trust in scientists across countries. 16

Fig. S9. Marginal effects plots for independent variables on trust in scientists as shown in Table S2 17

Fig. S10. Random effects of weighted regression model explaining the relationship of religiosity and trust in scientists across countries. 21

Fig. S11. Random effects of weighted regression model explaining the relationship of political orientation (right) and trust in scientists across countries. 22

Fig. S12. Random effects of weighted regression model explaining the relationship of political orientation (conservative) and trust in scientists across countries. 23

Country-specific explanations for the positive relationship between right-leaning political orientation and trust in scientists in selected countries 24
Fig. S13. Average levels of trust in scientists plotted against Gini index across countries. 25
Table S3. Weighted linear multilevel regression predicting trust in scientists (random intercepts across countries) including interaction effects of Gini index and Corruption Perceptions Index as well as Gini index and Overrepresentation of urban population........ 26
Fig. S14. Comparison of rankings of trust in scientists (TISP data) and trust in the national government (Global Wellcome Monitor data).. 27
Fig. S15. Weighted means and standard errors of normative perceptions of scientists' engagement across countries.. 28
Table S4. Weighted linear multilevel regression explaining normative perceptions of science (random intercepts across countries)... 29
Fig. S16. Random effects of weighted regression model explaining the relationship of trust in scientists and normative perceptions of science in society and policymaking across countries. 30
Table S5. Weighted linear multilevel regressions explaining whether perceived priorities exceed desired priorities (random effects of trust in scientists and science-related populist attitudes across countries) 32
Table S6. Weighted linear multilevel regressions predicting trust in scientists with perceptions that science's efforts to tackle four goals (i.e., perceived priorities) exceed expectations for scientists to prioritize these goals (i.e., desired priorities; random effects of perceived-desired discrepancy scores and science-related populist attitudes across countries).. 36
Fig. S17. Weighted means and standard errors of desired priority for developing defence and military technology across countries.. 37
Table S7. Weighted zero-order correlations of trust in scientists with perceived research priorities. 38
Table S8. Weighted zero-order correlations of trust in scientists with desired research priorities. 39
Sensitivity tests for different trust measures 40
Table S9. Weighted zero-order correlations of trust index with other trust measures and dimension scores.. 41
Table S10. Weighted linear multilevel regression explaining confidence in scientists to act in public interest... 42
Table S11. Weighted linear multilevel regression explaining trust in the scientific method (random intercepts across countries) 46
Fig. S18. Weighted means and standard errors of competence dimension of trust in scientists across countries 50
Fig. S19. Weighted means and standard errors of integrity dimension of trust in scientists across countries.. 51

Fig. S20. Weighted means and standard errors of benevolence dimension of trust in scientists across countries ... 52

Fig. S21. Weighted means and standard errors of openness dimension of trust in scientists across countries ... 53

Fig. S22. Weighted means and standard errors of confidence that scientists act in public interests across countries ... 54

Fig. S23. Weighted means and standard errors of trust in the scientific method across countries ... 55

Fig. S24. Standardised estimates of weighted blockwise multilevel regression model testing the association of confidence in scientists to act in public interest with demographic characteristics, ideological views, attitudes, and country-level indicators (random intercepts across countries) ... 56

Fig. S25. Standardised estimates of weighted blockwise multilevel regression model testing the association of trust in the scientific method with demographic characteristics, ideological views, attitudes, and country-level indicators (random intercepts across countries) ... 57

Fig. S26. Random effects of political orientation (right) on confidence in scientists across countries ... 58

Fig. S27. Random effects of political orientation (right) on trust in scientific method across countries ... 59

Limitations ... 60

Supplementary Methods ... 62

Replication materials ... 62

Countries sampled ... 62

Table S12. Representativeness of surveyed countries by income groups. ... 62

Table S13. Representativeness of surveyed countries by regions. ... 63

Market research companies ... 63

Preregistration ... 63

Table S14. Preregistered research questions and hypotheses whose test results are not presented in the main article. ... 64

Power analysis ... 65

Fig. S28. Power curve of simulation-based power analysis for effects of trust in scientists (b = 0.20, k = 3 countries) ... 66

Fig. S29. Power curve of simulation-based power analysis for effects of science-related populist attitudes (b = 0.10, k = 3 countries). ... 67

Fig. S30. Power curve of simulation-based power analysis for effects of trust in scientists (b = 0.10, k = 15 countries) ... 68

Fig. S31. Power curve of simulation-based power analysis for effects of science-related populist attitudes (b = 0.05, k = 15 countries). ... 69

The dataset......70
Country indicators......70
Analytical Procedures......72
Indices......72
Scaling......73
Analysis of average levels of trust in scientists......73
Analysis of factors explaining trust in scientists......74
Table S15. Weighted zero-order correlations of trust in scientists and the attitudes to science. 75
Table S16. Variance inflation factors for weighted linear multilevel regression model explaining trust in scientists. 76
Table S17. Variance inflation factors for weighted linear multilevel regression model explaining normative perceptions of science in society and policymaking. 77
Analysis of factors explaining perceived and desired priorities of scientific research 77
Table S18. Variance inflation factors for weighted linear multilevel regression models explaining the discrepancy of desires that scientists should prioritize certain issues and perceptions that science addresses these issues. 78
Table S19. Variance inflation factors for weighted linear multilevel regression model explaining trust in scientists with perceptions that science's efforts to tackle four goals (i.e., perceived priorities) exceed expectations for scientists to prioritize these goals (i.e., desired priorities)......79
Table S20. Valid N across countries for analyses testing means and standard errors across countries......80
Table S21. Valid N across countries for multilevel regressions......82
Table S22. Overview IRB applications for ethics approval......84
Additional References......88

Results

There are distinct causal pathways that could be hypothesized and tested. We focus on this particular causal model depicted in our directed acyclic graph (DAG; fig. S1a) which is in line with previous global studies on trust[6,90,91]. For an alternative DAG proposed by an anonymous reviewer see fig. S1b. Our data is publicly available, and we encourage other researchers to explore this and other alternative models.

Fig. S1a. Directed acyclic graph (DAG) underlying results presented in Fig. 1

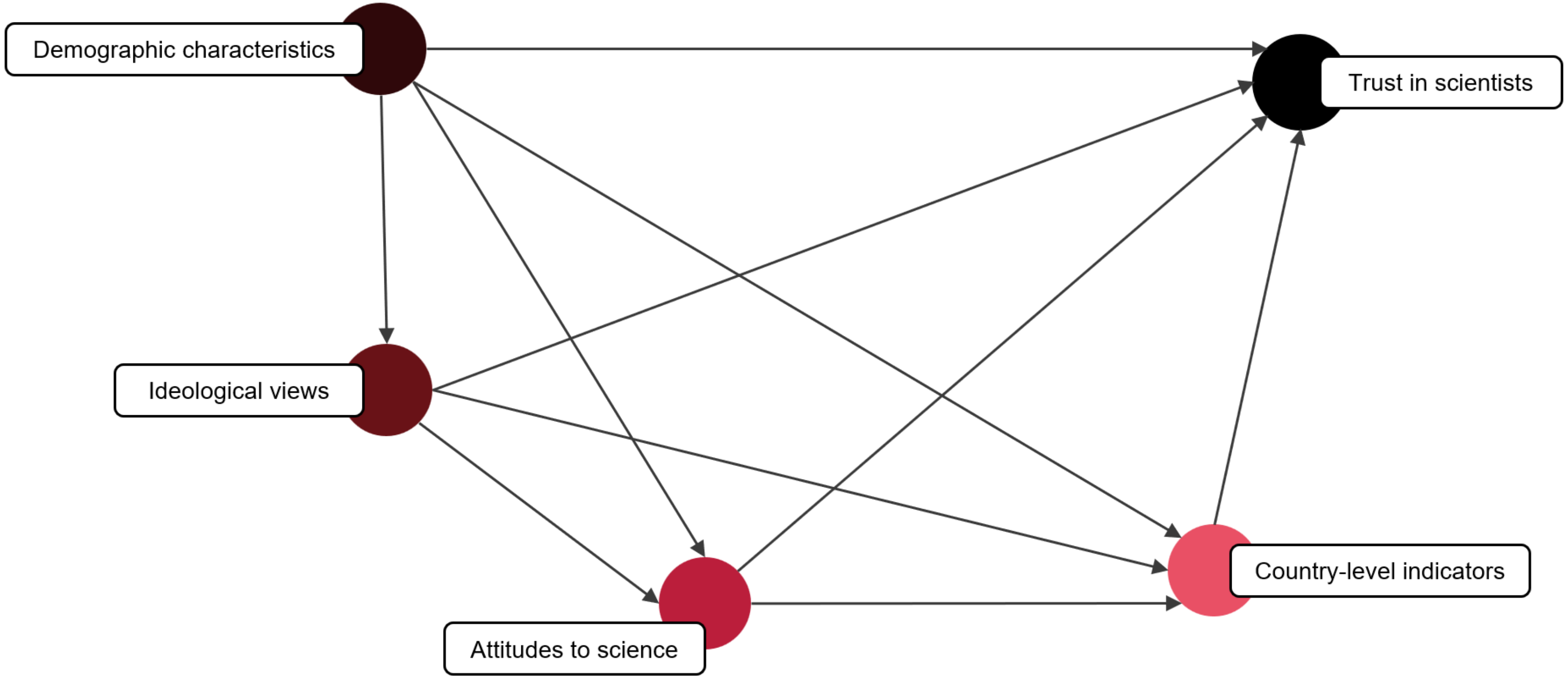

Fig. S1b. Alternative DAG proposed by an anonymous reviewer

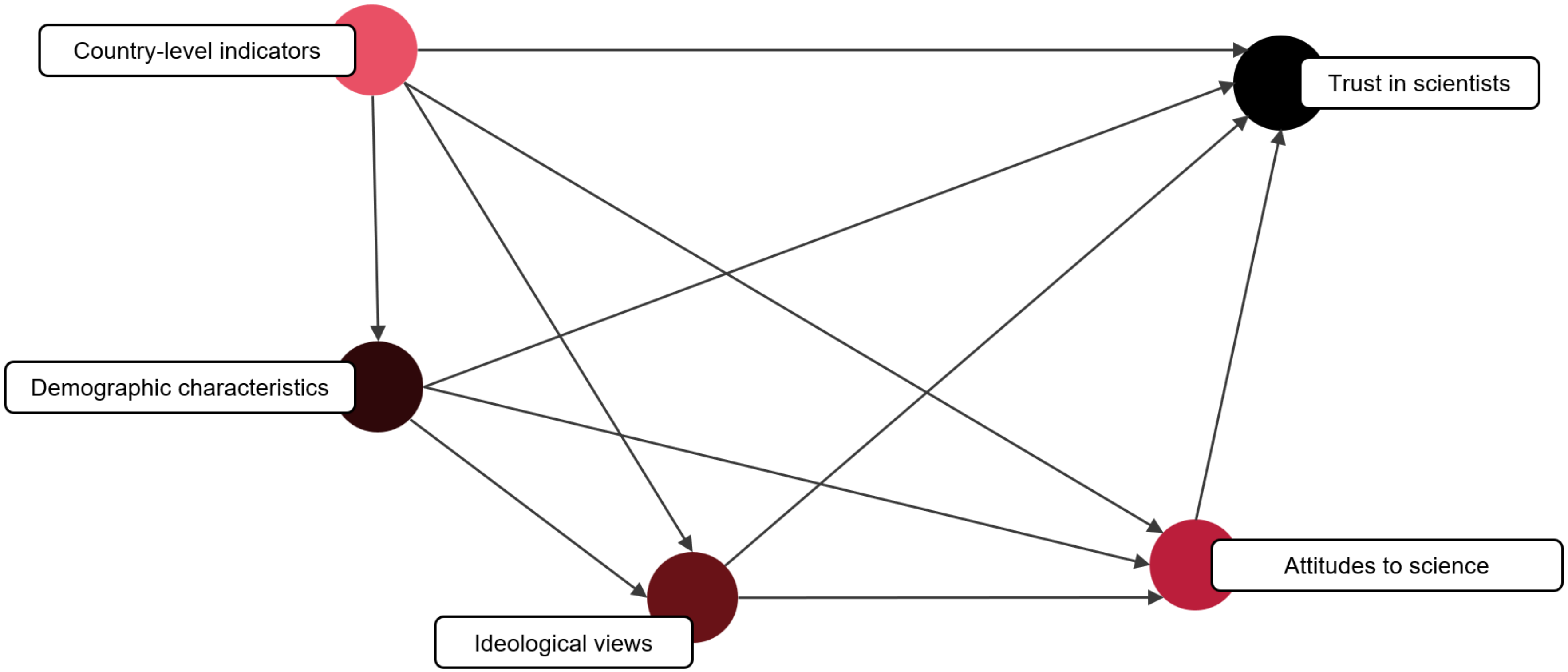

Fig. S2. Valid sample sizes across countries.

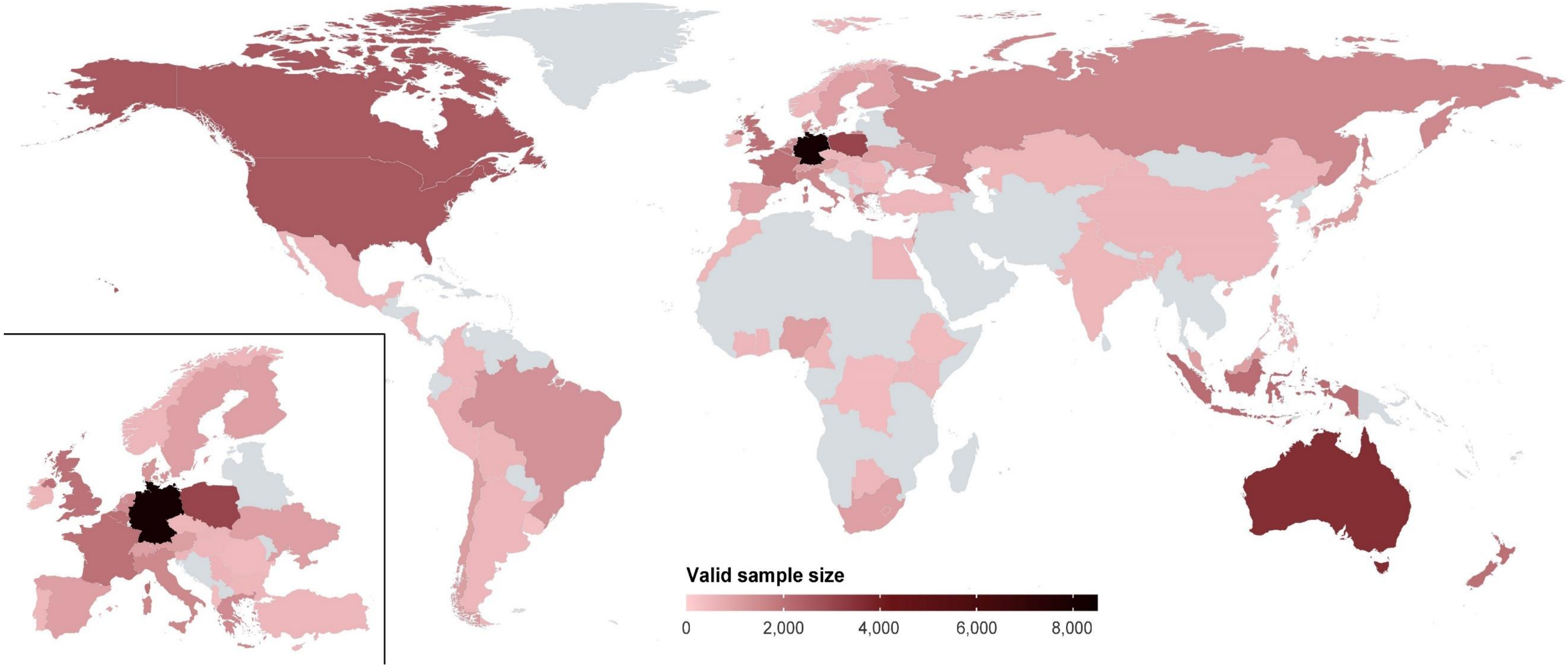

Note: See Mede et al.[46] for valid sample sizes across countries.

Table S1. Weighted grand mean, median, SD, and SE for the 12-item index measuring trust in scientists, the four 3-item mean indices measuring the four dimensions, the 12 individual items, as well as the 1-item measures for confidence in scientists and trust in the scientific method.

Variable	*n*	*M*	*SE*	*SD*	*Me*
**Confidence that scientists act in the best interests of the public**	69,507	3.57	0.01	1.06	4.00
**Trust in the scientific method**	69,521	4.07	0.01	0.96	4.00
**Trust in scientists (12-item index)**	69,527	3.62	<0.001	0.70	3.67
Competence	69,526	4.02	<0.001	0.71	4.00
How expert or inexpert are most scientists?	69,506	3.85	0.01	0.89	4.00
How intelligent or unintelligent are most scientists?	69,502	4.20	<0.001	0.81	4.00
How qualified or unqualified are most scientists when it comes to conducting high-quality research?	69,500	4.02	0.01	0.86	4.00
Integrity	69,526	3.58	<0.001	0.78	3.67
How honest or dishonest are most scientists?	69,501	3.58	0.01	0.92	4.00
How ethical or unethical are most scientists?	69,505	3.55	0.01	0.90	4.00
How sincere or insincere are most scientists?	69,500	3.62	0.01	0.92	4.00
Benevolence	69,522	3.55	0.01	0.82	3.67
How concerned or not concerned are most scientists about people’s well-being?	69,501	3.53	0.01	1.00	4.00
How eager or uneager are most scientists to improve others’ lives?	69,501	3.72	0.01	0.94	4.00
How considerate or inconsiderate are most scientists of others’ interests?	69,488	3.41	0.01	0.97	3.00
Openness	69,523	3.33	0.01	0.86	3.33
How open are most scientists to feedback?	69,498	3.32	0.01	1.02	3.00
How willing or unwilling are most scientists to be transparent?	69,505	3.40	0.01	1.01	3.00
How much or little attention do scientists pay to others' views?	69,489	3.26	0.01	1.03	3.00

Note: Response options varied for each item, e.g., 1 = very [inexpert], 3 = neither [expert] nor [inexpert], 5 = very [expert]

Fig. S3. Weighted response probabilities for single trust in scientists scale items.

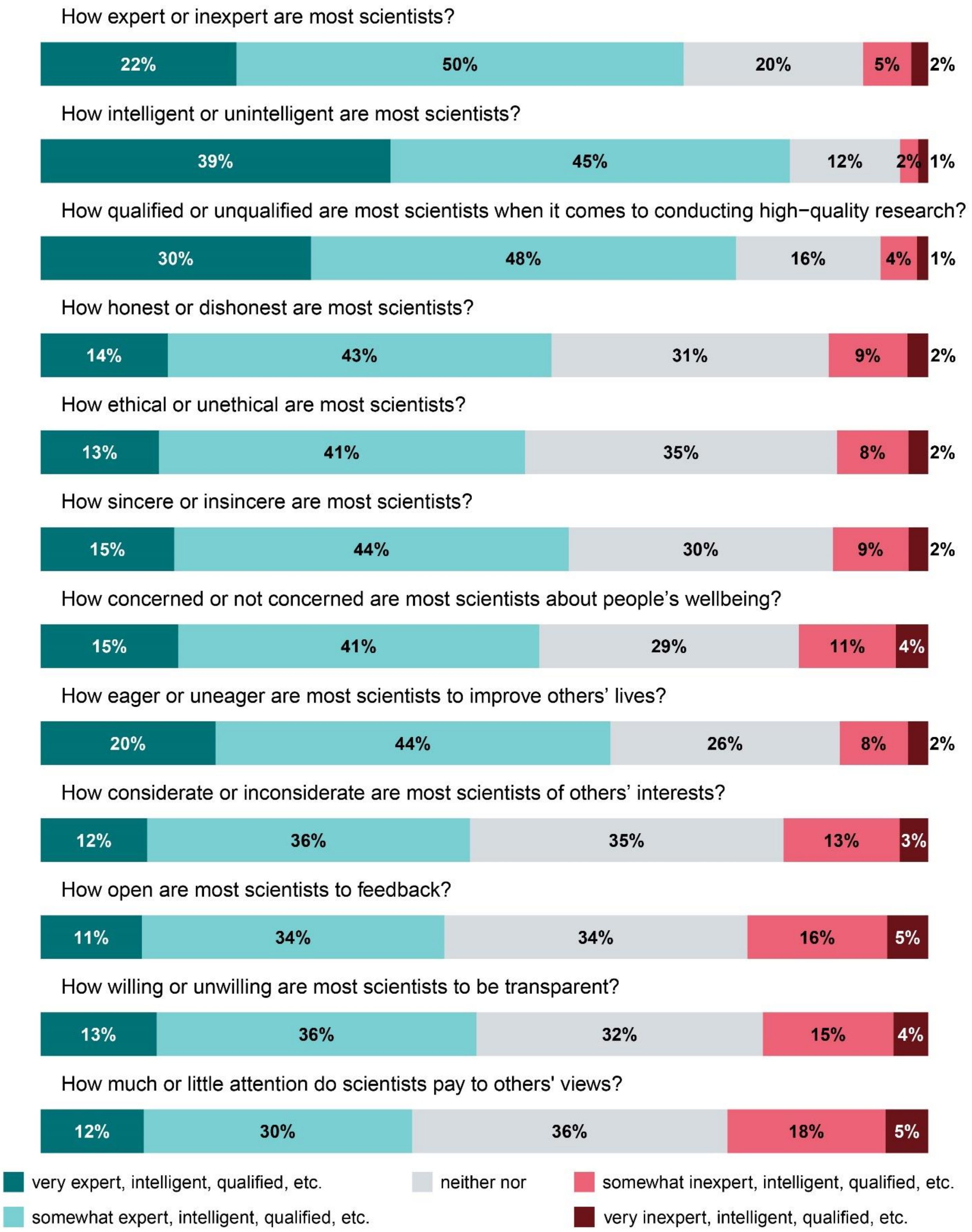

Note: Response options were adapted for each item, e.g., very sincere, very open, very honest

Table S2. Weighted linear multilevel regression testing the association of trust in scientists with demographic characteristics, attitudes to science, and country-level indicators (random intercepts across countries)

	**Block 1: Demographic characteristics**						**Block 2: Ideological views**					
Predictors	*Beta*	*SE*	*CI*	*t*	*p*	*df*	*Beta*	*SE*	*CI*	*t*	*p*	*df*
Intercept	3.654	0.03	3.593 – 3.714	120.445	**<.001**	64.424	3.694	0.031	3.633 – 3.755	121.1	**<.001**	63.309
Gender (male)	-0.019	0.003	-0.024 – -0.014	-7.246	**<.001**	64389.178	-0.012	0.003	-0.018 – -0.006	-3.913	**<.001**	47595.511
Age	0.029	0.002	0.024 – 0.034	11.906	**<.001**	64426.306	0.016	0.003	0.010 – 0.021	5.701	**<.001**	47631.639
Education (tertiary)	0.035	0.003	0.029 – 0.040	12.556	**<.001**	63979.236	0.031	0.003	0.025 – 0.037	10.082	**<.001**	47298.793
Income	0.035	0.003	0.030 – 0.040	13.402	**<.001**	64396.237	0.025	0.003	0.019 – 0.031	8.15	**<.001**	47599.032
Residence place (urban)	0.029	0.003	0.024 – 0.034	11.263	**<.001**	64385.976	0.03	0.003	0.024 – 0.036	10.245	**<.001**	47594.754
Political orientation (right)							0.007	0.004	0.000 – 0.014	1.964	**.049**	47597.812
Political orientation (conservative)							-0.034	0.004	-0.041 – -0.027	-9.624	**<.001**	47600.43
Religiosity							0.051	0.003	0.045 – 0.057	16.679	**<.001**	47596.763
Social dominance orientation							-0.098	0.003	-0.104 – -0.092	-31.982	**<.001**	47602.442
Science-related populist attitudes												
Perceived benefit of science												
Willingness to be vulnerable to science												
Trust in the scientific method												
GDP per capita												
Gvt expenditure on education (% of GDP)												
Gini index												
Science literacy (PISA)												
Academic freedom												
Degree of populism in politics												
**Random Effects**												
σ^2	0.26						0.25					
τ_{00}	0.06 COUNTRY_NAME						0.06 COUNTRY_NAME					
ICC	0.19						0.19					
N	68 COUNTRY_NAME						67 COUNTRY_NAME					
Observations	64458						47664					
Marginal R^2 / Conditional R^2	0.015 / 0.200						0.052 / 0.235					
AIC	168639.87						121133.099					

Predictors	Block 3: Attitudes to science						Block 4: Country indicators					
	Beta	*SE*	*CI*	*t*	*p*	*df*	*Beta*	*SE*	*CI*	*t*	*p*	*df*
Intercept	3.627	0.031	3.566 – 3.689	117.934	**<.001**	62.556	3.616	0.031	3.554 – 3.678	117.571	**<.001**	42.87
Gender (male)	-0.025	0.002	-0.029 – -0.020	-10.305	**<.001**	46632.177	-0.024	0.003	-0.029 – -0.019	-9.409	**<.001**	41567.82
Age	0.007	0.002	0.002 – 0.011	2.962	**0.003**	46658.735	0.007	0.002	0.003 – 0.012	3.162	**0.002**	41581.972
Education (tertiary)	-0.014	0.002	-0.019 – -0.009	-5.595	**<.001**	46595.305	-0.014	0.003	-0.019 – -0.009	-5.365	**<.001**	41608.998
Income	0.005	0.002	-0.000 – 0.009	1.899	0.058	46634.709	0.005	0.003	-0.000 – 0.010	1.937	0.053	41566.65
Residence place (urban)	0.012	0.002	0.008 – 0.017	5.212	**<.001**	46631.79	0.014	0.002	0.009 – 0.019	5.677	**<.001**	41566.073
Political orientation (right)	0.008	0.003	0.003 – 0.014	2.913	**0.004**	46633.56	0.008	0.003	0.003 – 0.014	2.822	**0.005**	41567.126
Political orientation (conservative)	-0.008	0.003	-0.013 – -0.002	-2.668	**0.008**	46635.737	-0.009	0.003	-0.015 – -0.003	-2.997	**0.003**	41566.386
Religiosity	0.051	0.002	0.046 – 0.055	20.4	**<.001**	46633.051	0.05	0.003	0.045 – 0.055	19.269	**<.001**	41566.853
Social dominance orientation	-0.014	0.003	-0.019 – -0.009	-5.549	**<.001**	46636.959	-0.015	0.003	-0.020 – -0.010	-5.725	**<.001**	41568.514
Science-related populist attitudes	-0.022	0.003	-0.027 – -0.017	-8.721	**<.001**	46631.76	-0.021	0.003	-0.026 – -0.016	-8.035	**<.001**	41566.695
Perceived benefit of science	0.153	0.003	0.147 – 0.158	54.442	**<.001**	46636.946	0.152	0.003	0.146 – 0.158	51.502	**<.001**	41567.691
Willingness to be vulnerable to science	0.184	0.003	0.178 – 0.190	64	**<.001**	46635.144	0.186	0.003	0.180 – 0.192	61.653	**<.001**	41568.727
Trust in the scientific method	0.188	0.003	0.182 – 0.194	65.457	**<.001**	46634.858	0.187	0.003	0.181 – 0.193	62.055	**<.001**	41566.861
GDP per capita							0.024	0.042	-0.060 – 0.109	0.577	0.567	42.423
Gvt expenditure on education (% of GDP)							0.044	0.036	-0.029 – 0.117	1.227	0.226	43.33
Gini index							0.095	0.033	0.028 – 0.162	2.866	**0.006**	43.063
Science literacy (PISA)							0.017	0.036	-0.055 – 0.090	0.479	0.635	44.837
Academic freedom							0.013	0.035	-0.058 – 0.085	0.381	0.705	43.197
Degree of populism in politics							-0.024	0.032	-0.089 – 0.040	-0.762	0.45	43.511
**Random Effects**												
σ^2	0.15						0.16					
τ_{00}	0.06 COUNTRY_NAME						0.04 COUNTRY_NAME					
ICC	0.28						0.2					
N	66 COUNTRY_NAME						51 COUNTRY_NAME					
Observations	46707						41629					
Marginal R^2 / Conditional R^2	0.427 / 0.588						0.453 / 0.562					
AIC	97220.101						76574.332					

Note: Significant testing based on two-sided t tests. AIC = Akaike information criterion, ICC = Intraclass Correlation Coefficient. σ^2 = within-country (residual) variance. τ_{00} = between-country variance (variation between individual intercepts and average intercept).

Fig. S4. Standardised estimates of weighted blockwise multilevel regression model testing the association of perceived competence with demographic characteristics, ideological views, attitudes, and country-level indicators (random intercepts across countries)

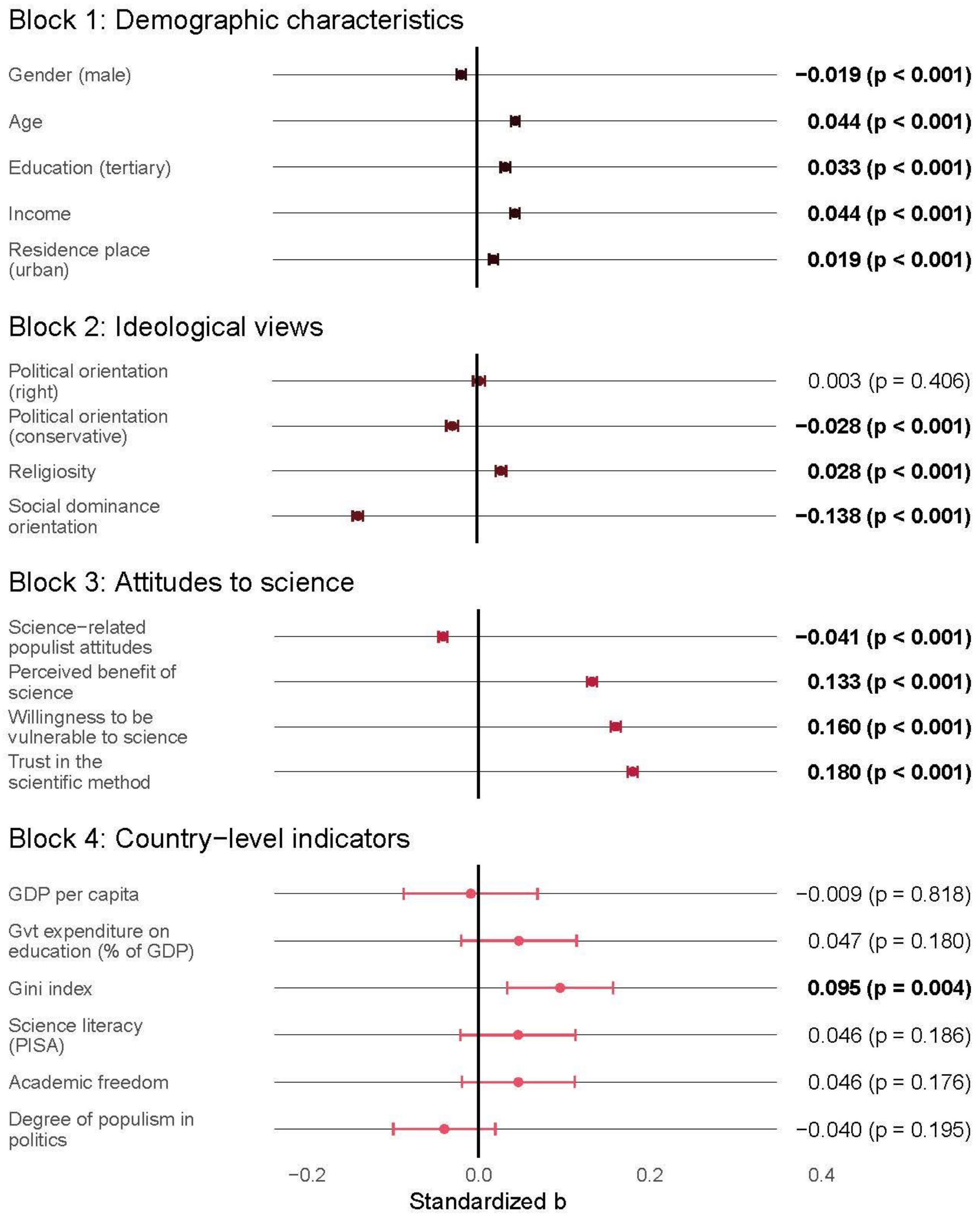

Note: Dots indicate point estimates of fixed effects, horizontal lines indicate 95% confidence intervals based on two-sided t tests. Block 1 uses data from all 68 countries, block 2 uses data from 67 countries (all except Malaysia, where social dominance orientation was not measured), block 3 uses data from 66 countries (all except Malaysia and Mexico, where willingness to be vulnerable to science was not measured), block 4 uses data from 51 countries (all except those where PISA's literacy scores were not available, see supplementary material).

Fig. S5. Standardised estimates of weighted blockwise multilevel regression model testing the association of perceived integrity with demographic characteristics, ideological views, attitudes, and country-level indicators (random intercepts across countries)

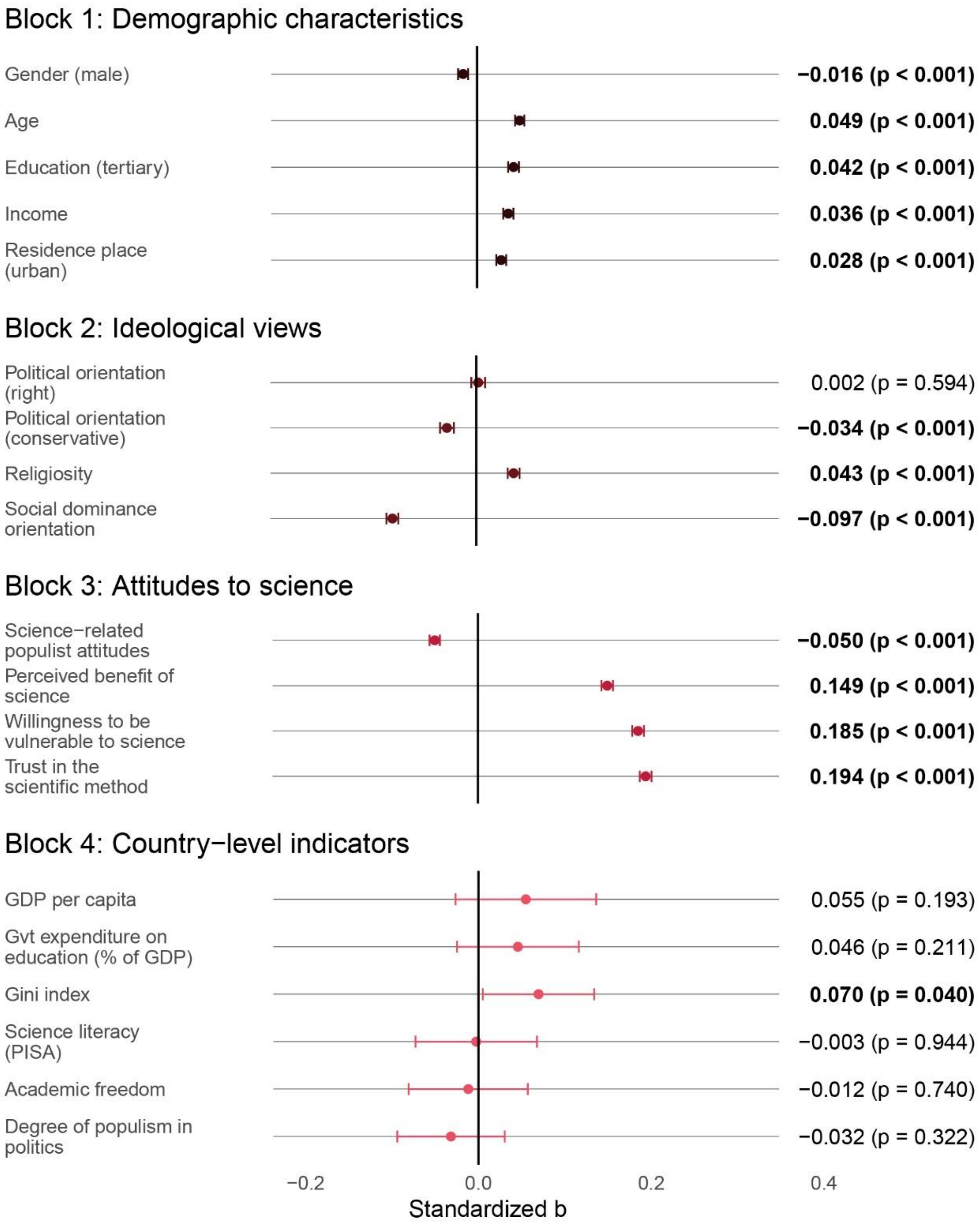

Note: Dots indicate point estimates of fixed effects, horizontal lines indicate 95% confidence intervals based on two-sided t tests. Block 1 uses data from all 68 countries, block 2 uses data from 67 countries (all except Malaysia, where social dominance orientation was not measured), block 3 uses data from 66 countries (all except Malaysia and Mexico, where willingness to be vulnerable to science was not measured), block 4 uses data from 51 countries (all except those where PISA's literacy scores were not available, see supplementary material).

Fig. S6. Standardised estimates of weighted blockwise multilevel regression model testing the association of perceived benevolence with demographic characteristics, ideological views, attitudes, and country-level indicators (random intercepts across countries)

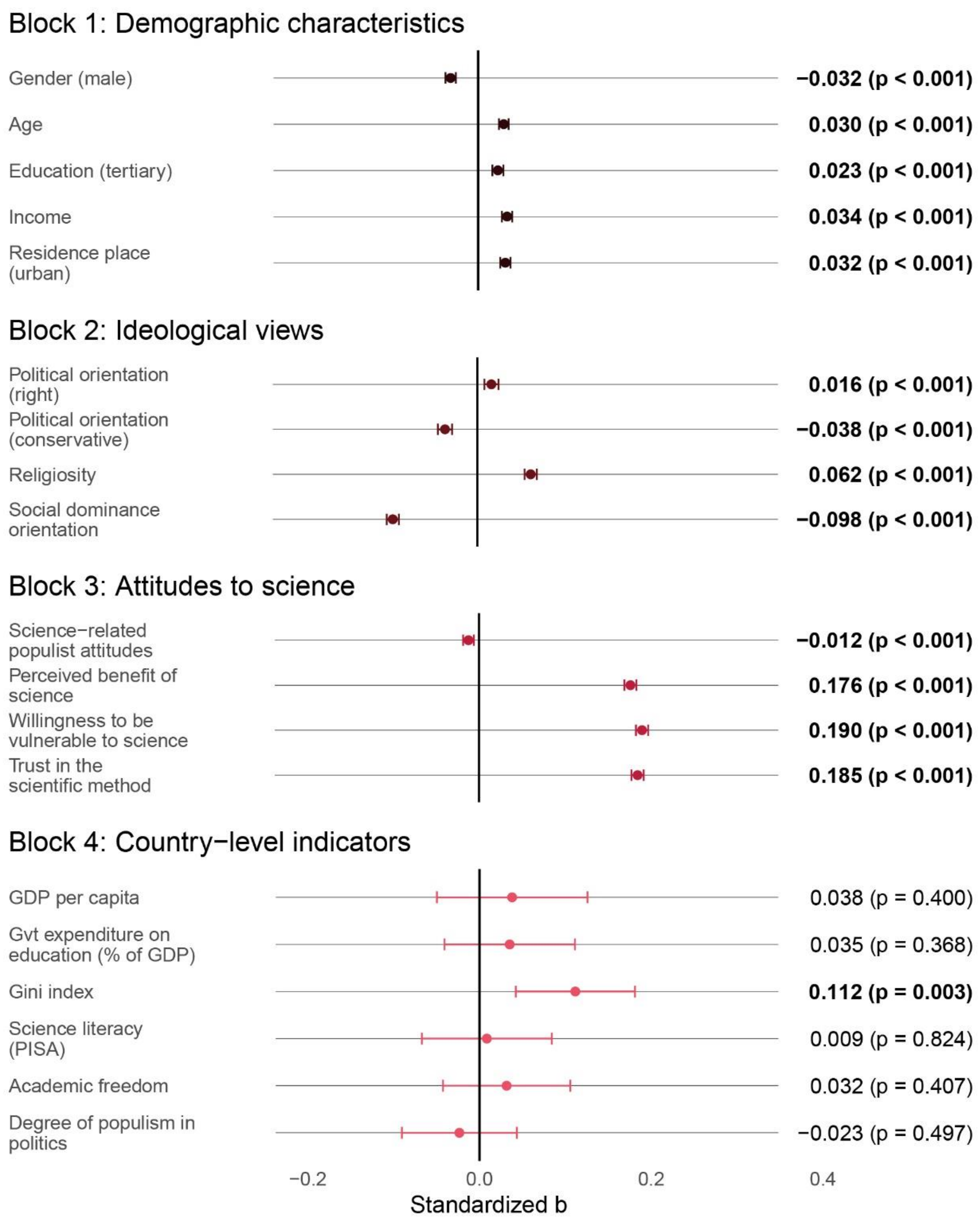

Note: Dots indicate point estimates of fixed effects, horizontal lines indicate 95% confidence intervals based on two-sided t tests. Block 1 uses data from all 68 countries, block 2 uses data from 67 countries (all except Malaysia, where social dominance orientation was not measured), block 3 uses data from 66 countries (all except Malaysia and Mexico, where willingness to be vulnerable to science was not measured), block 4 uses data from 51 countries (all except those where PISA's literacy scores were not available, see supplementary material).

Fig. S7. Standardised estimates of weighted blockwise multilevel regression model testing the association of perceived openness with demographic characteristics, ideological views, attitudes, and country-level indicators (random intercepts across countries)

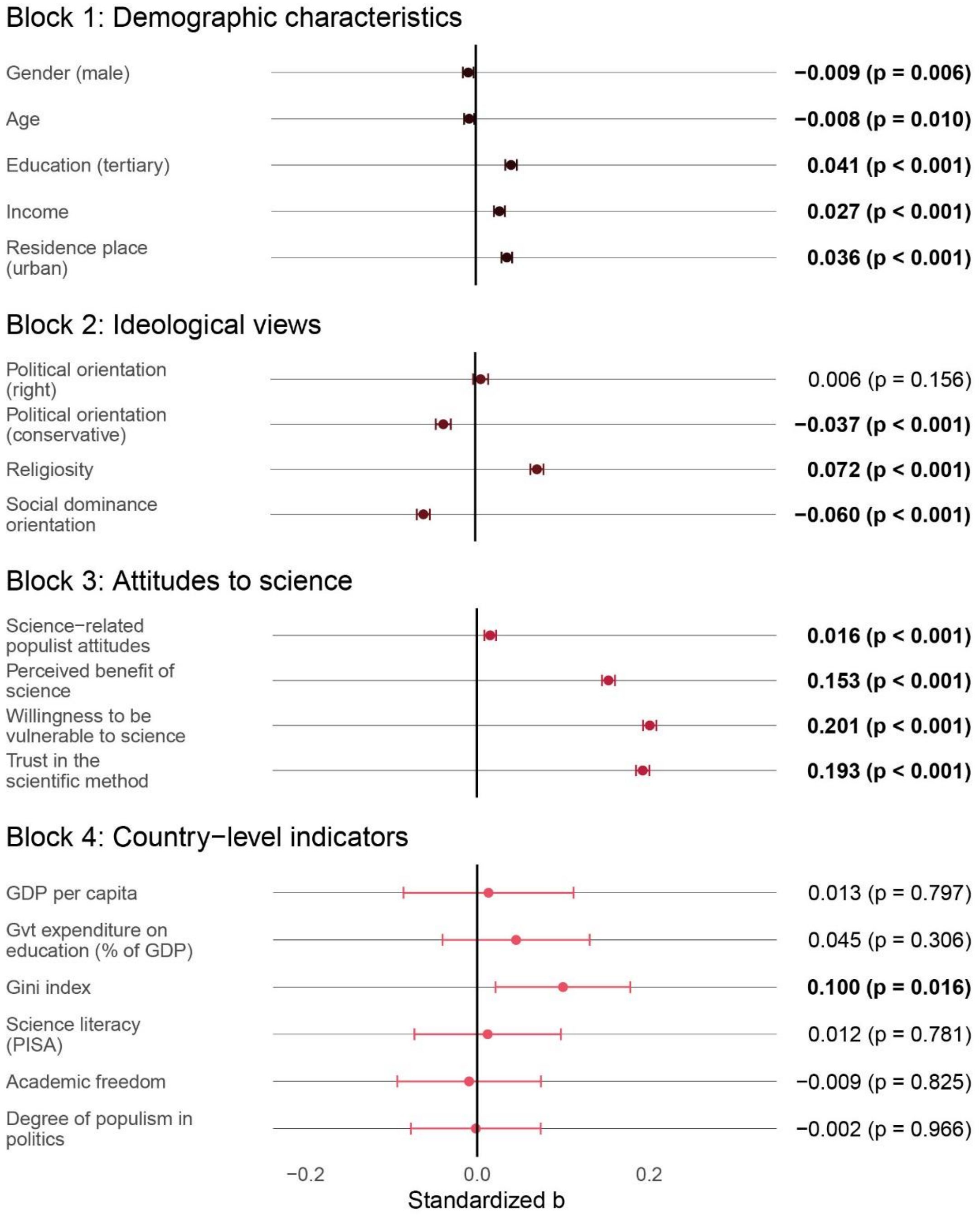

Note: Dots indicate point estimates of fixed effects, horizontal lines indicate 95% confidence intervals based on two-sided t tests. Block 1 uses data from all 68 countries, block 2 uses data from 67 countries (all except Malaysia, where social dominance orientation was not measured), block 3 uses data from 66 countries (all except Malaysia and Mexico, where willingness to be vulnerable to science was not measured), block 4 uses data from 51 countries (all except those where PISA's literacy scores were not available, see supplementary material).

Fig. S8. Random effects of weighted regression model explaining the relationship of education (tertiary) and trust in scientists across countries.

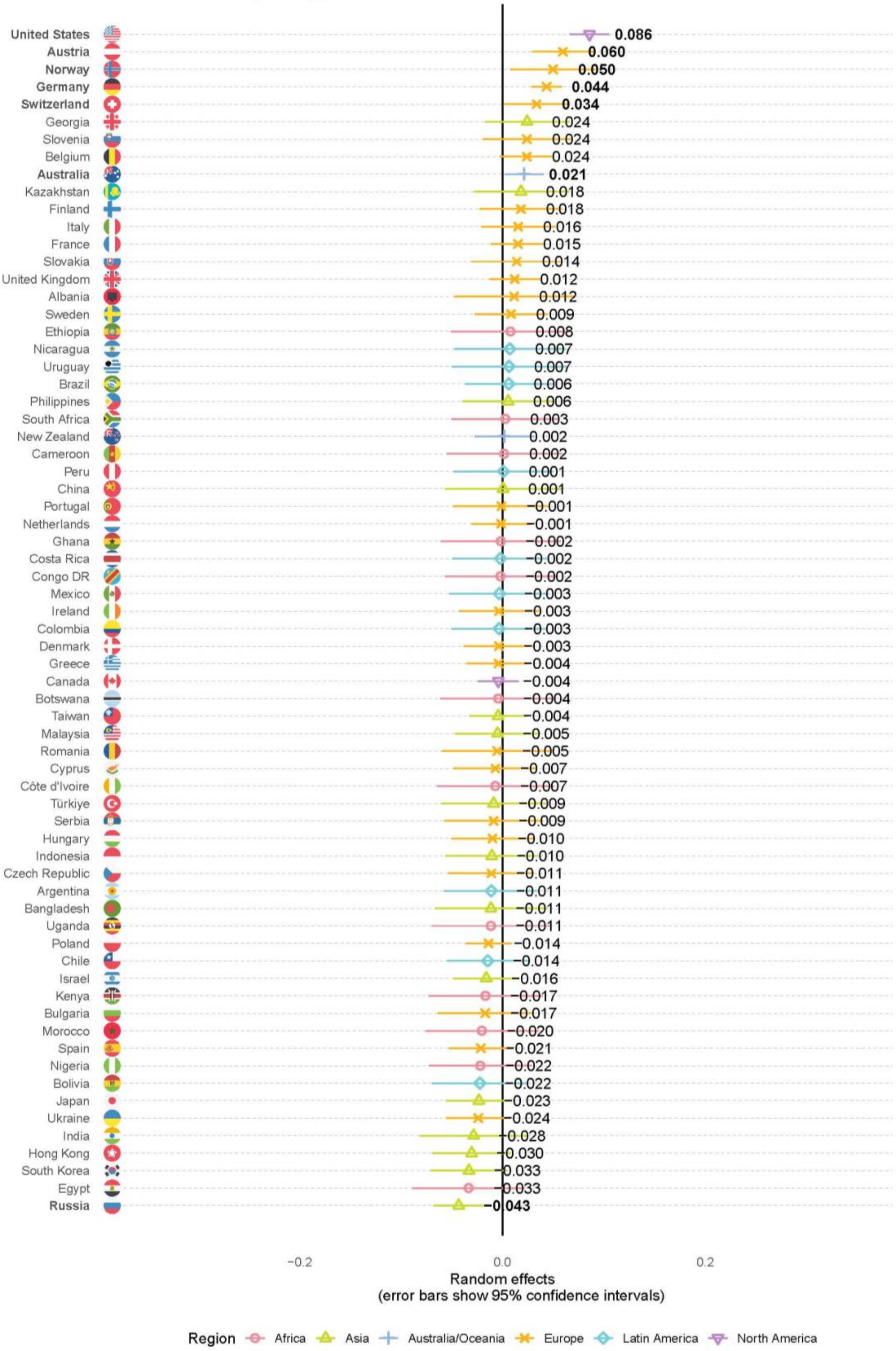

Note: Effects significant at $p < .05$ are printed in bold. Total N = 64,458. Country Ns range between 284 and 6,940 (see table S16 for N across countries).

Fig. S9. Marginal effects plots for independent variables on trust in scientists as shown in Table S2.

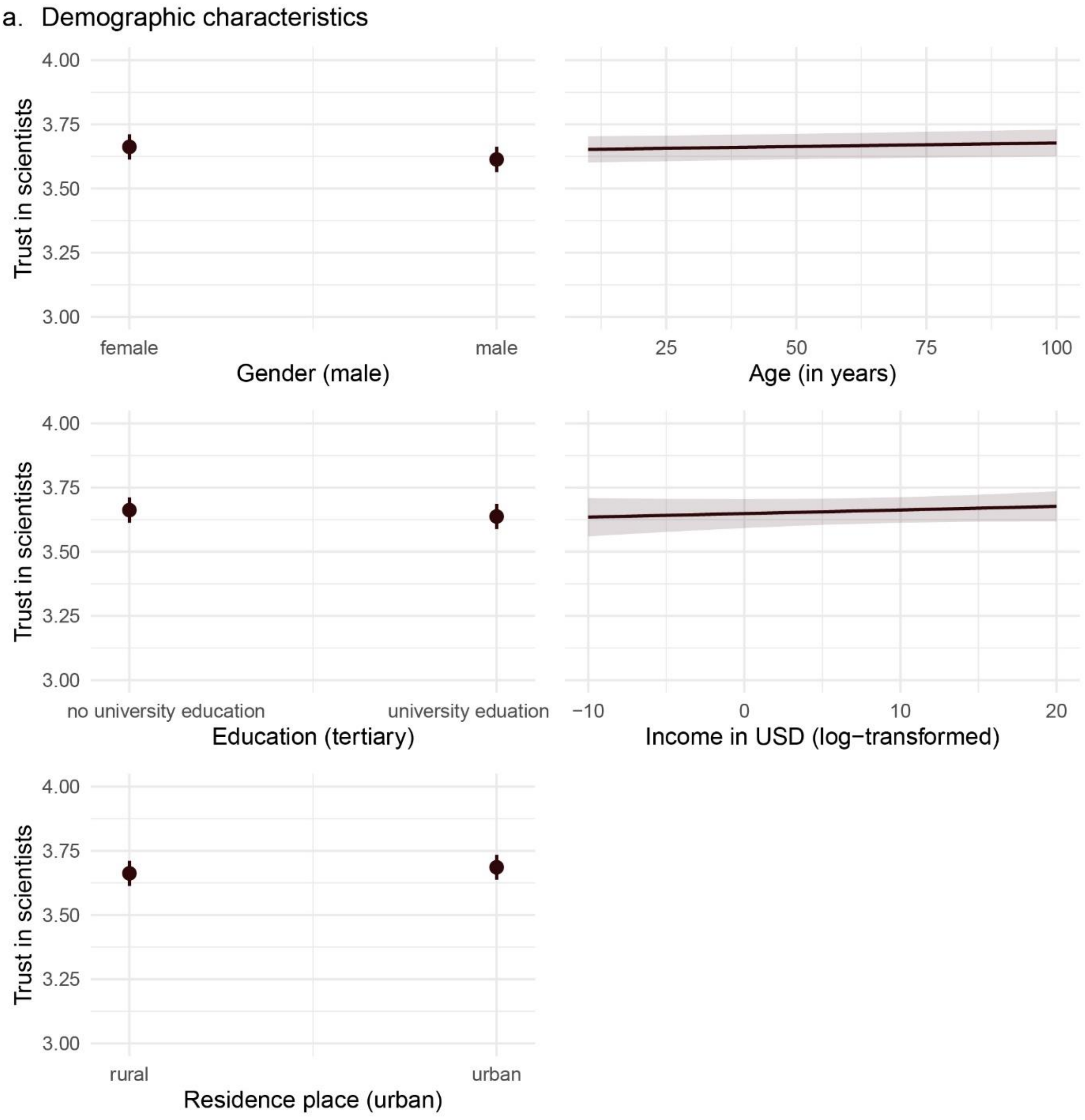

b. Ideological views

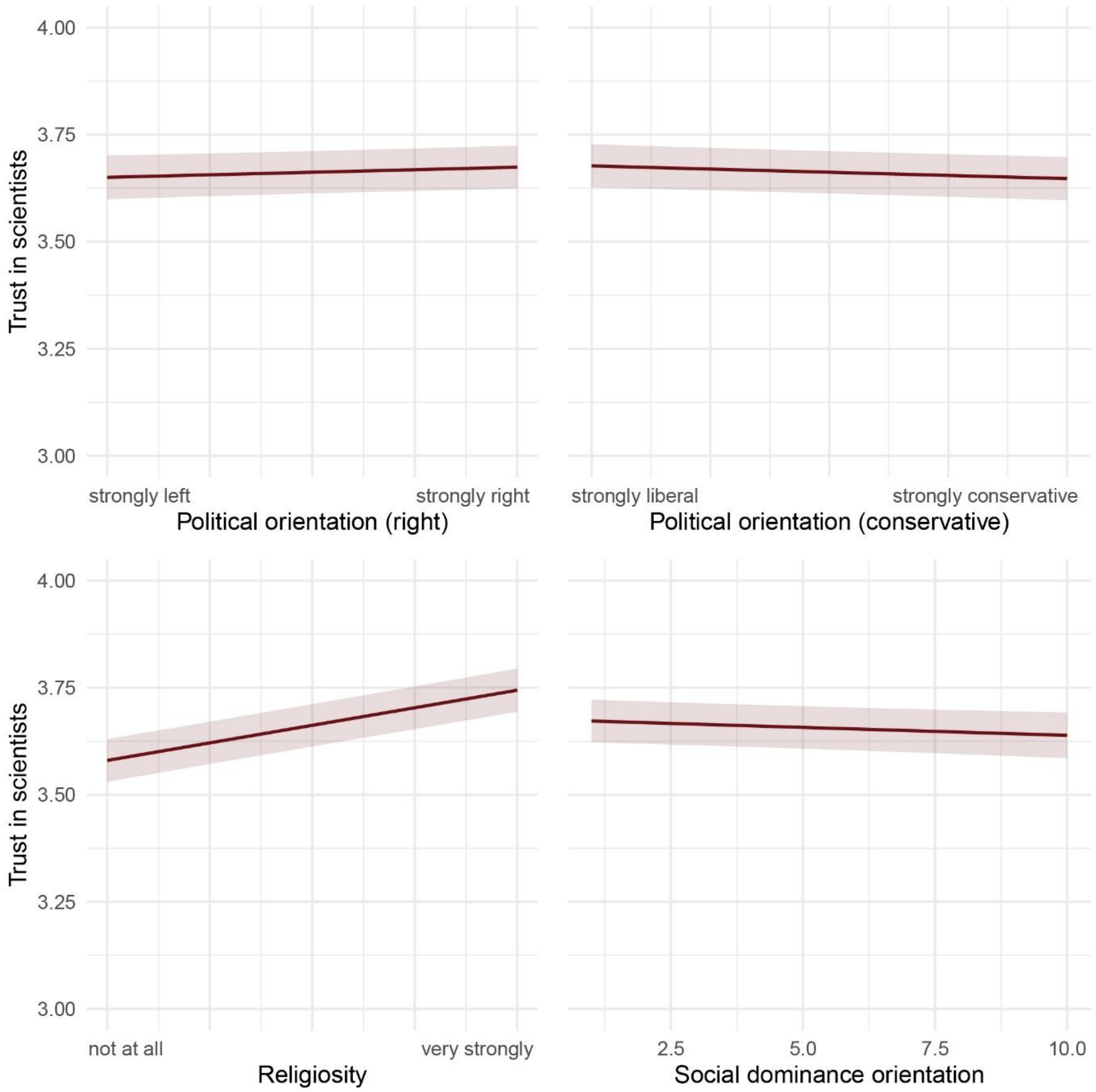

c. Attitudes to science

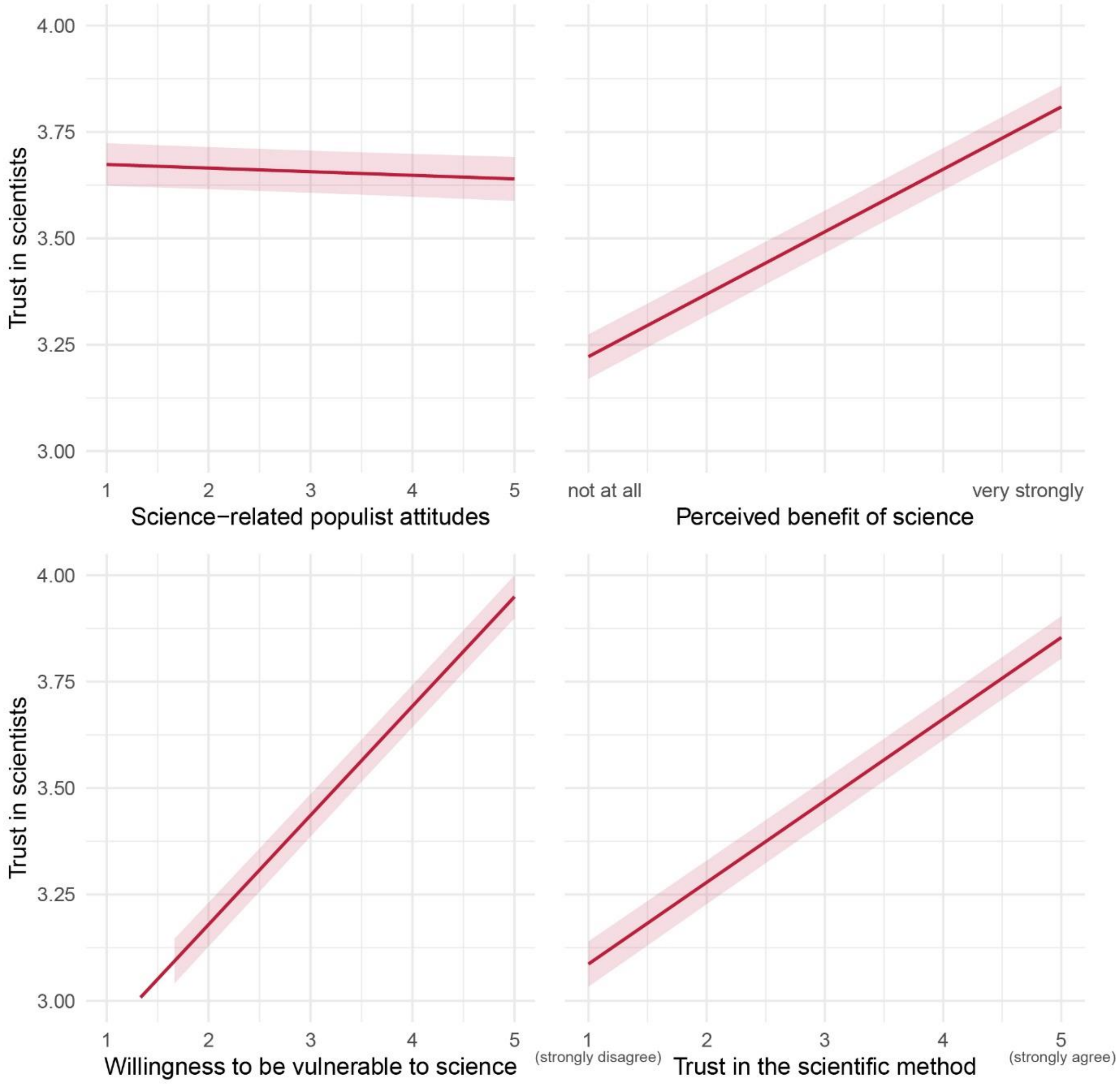

Note: See table S2 for number of observations. Error bands indicate 95% confidence intervals.

d. Country-level indicators

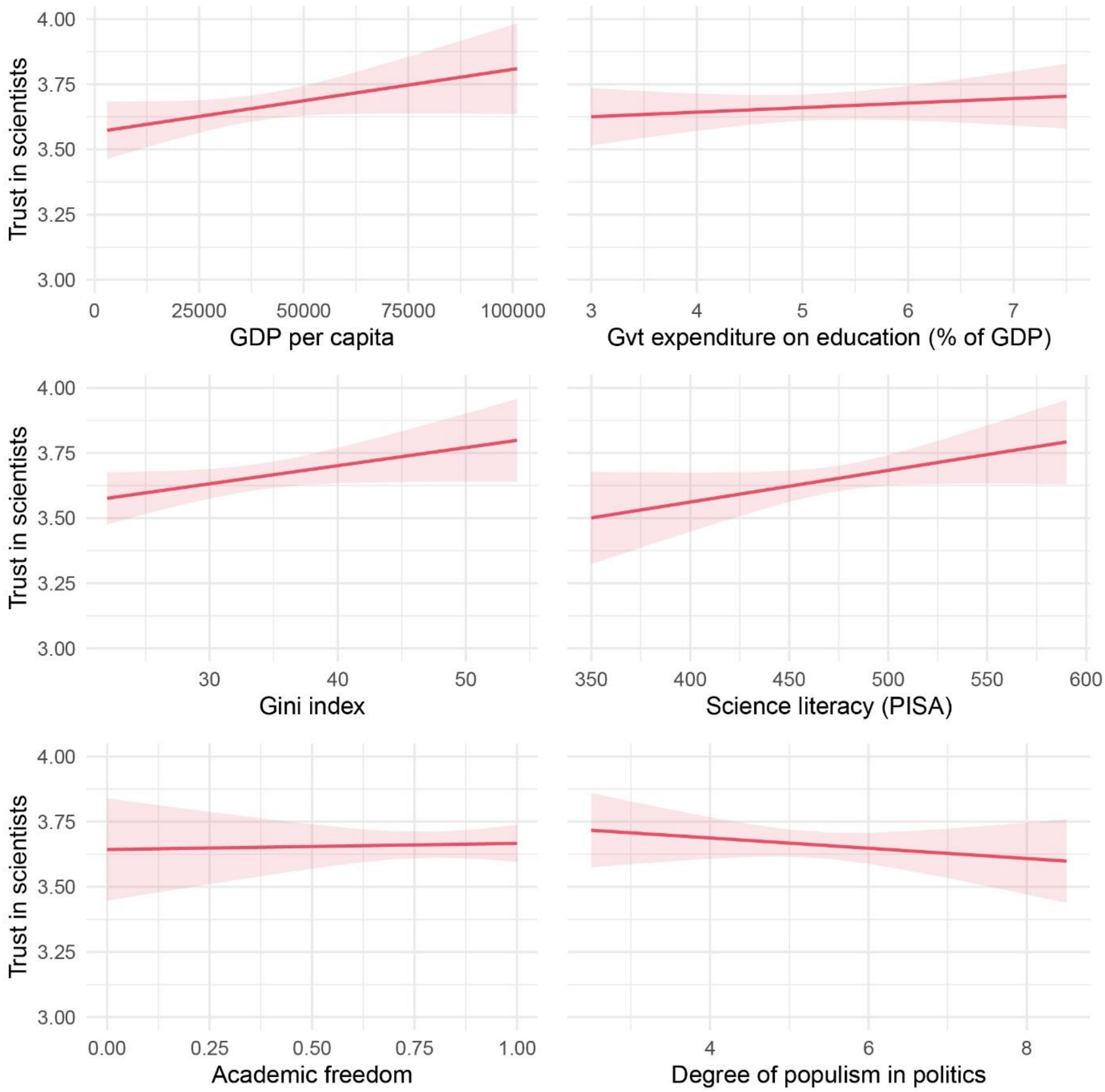

Fig. S10. Random effects of weighted regression model explaining the relationship of religiosity and trust in scientists across countries.

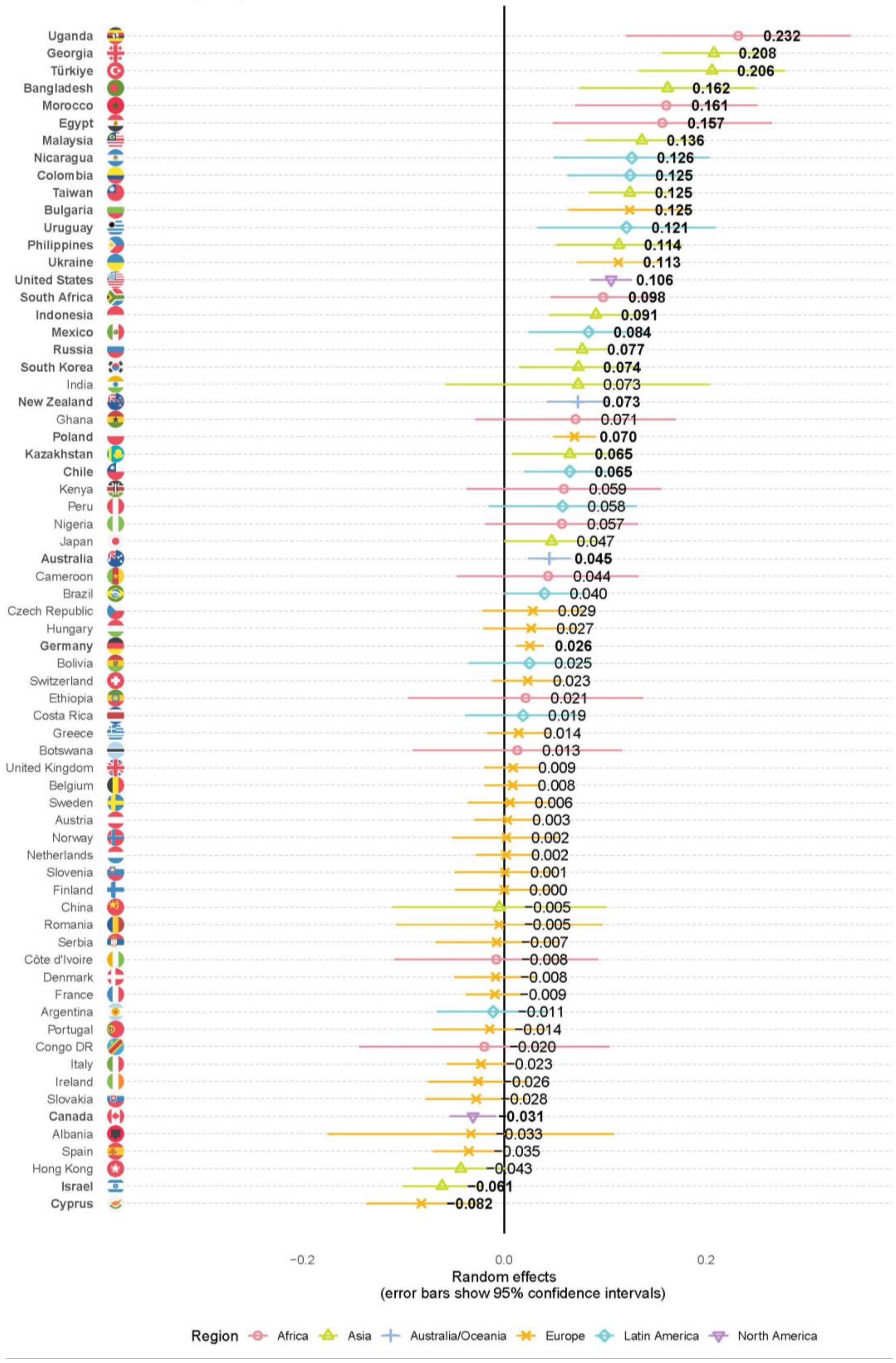

Note: Dots indicate point estimates of random effects, horizontal lines indicate 95% confidence intervals based on two-sided t tests. Effects significant at $p < .05$ are printed in bold. Total N = 64,458. Country Ns range between 284 and 6,940 (see table S16 for N across countries).

Fig. S11. Random effects of weighted regression model explaining the relationship of political orientation (right) and trust in scientists across countries.

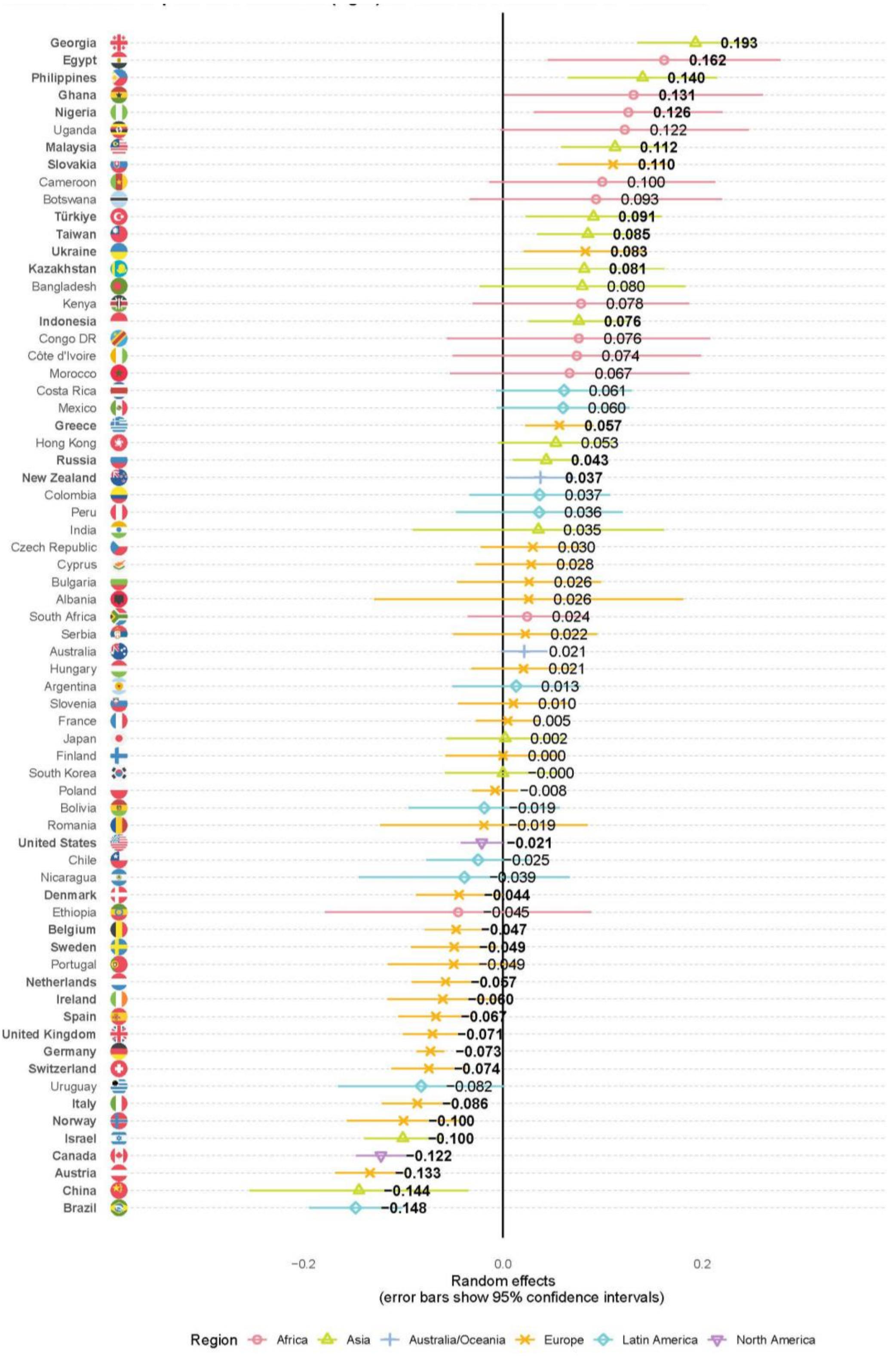

Note: Dots indicate point estimates of random effects, horizontal lines indicate 95% confidence intervals based on two-sided t tests. Effects significant at $p < .05$ are printed in bold. Total N = 64,458. Country Ns range between 284 and 6,940 (see table S16 for N across countries).

Fig. S12. Random effects of weighted regression model explaining the relationship of political orientation (conservative) and trust in scientists across countries.

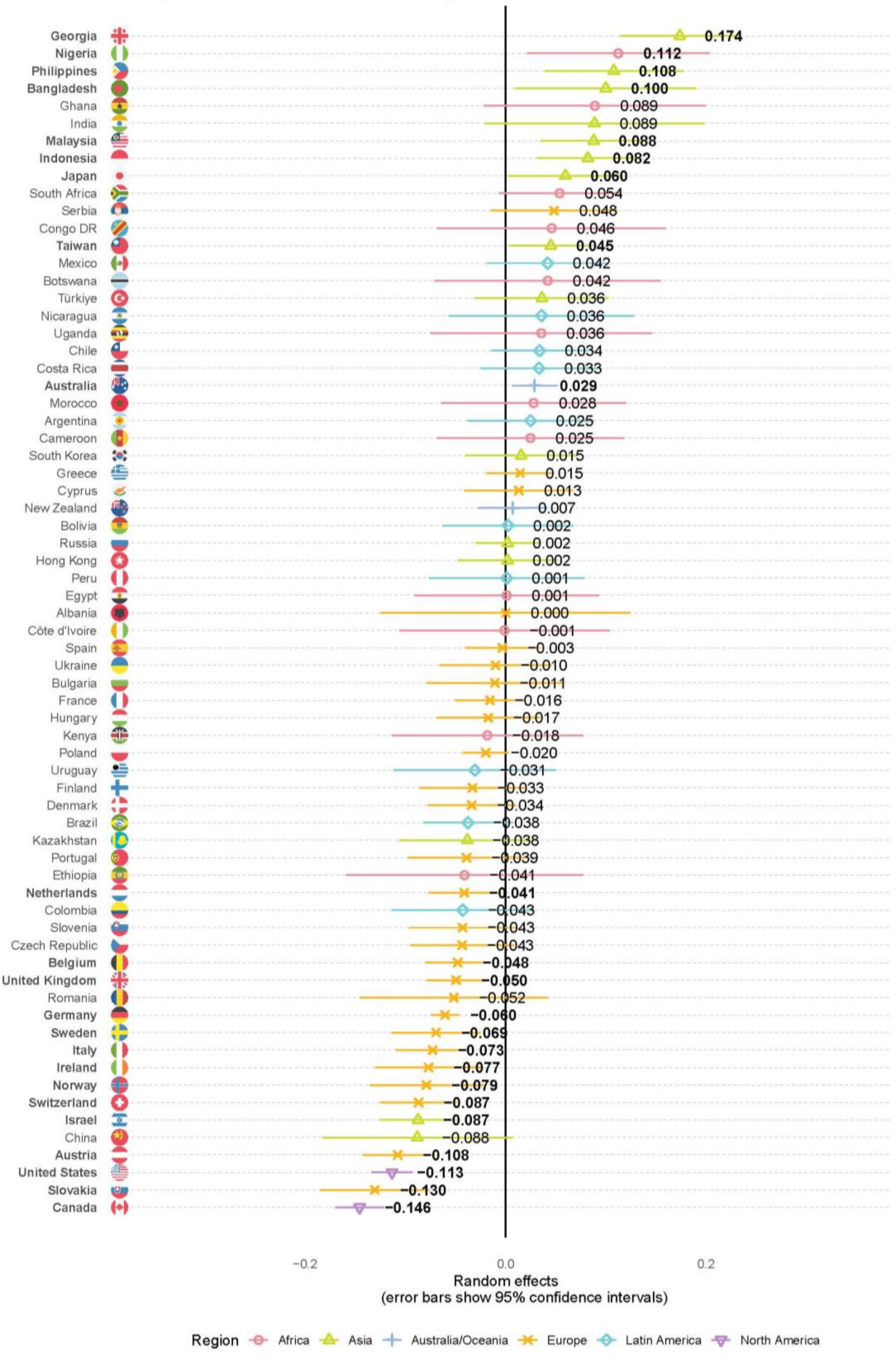

Note: Dots indicate point estimates of random effects, horizontal lines indicate 95% confidence intervals based on two-sided t tests. Effects significant at $p < .05$ are printed in bold. Total $N = 64{,}458$. Country Ns range between 284 and 6,940 (see table S16 for N across countries).

Country-specific explanations for the positive relationship between right-leaning political orientation and trust in scientists in selected countries

As seen in Fig. 3, right-leaning individuals have more trust in scientists in some countries. Given that previous studies have mostly focused on the reasons for distrust in scientists among right-leaning individuals, we discuss why right-leaning people may have higher trust in scientists in some countries. These explanations have been informed by our co-authors in their respective countries.

In the Philippines, left-wing political leaders have promoted reservations and scepticism towards science. At the same time, the former president of the Philippines Rodrigo Duterte, leader of a left-leaning party, heavily promoted vaccination against COVID-19 and insisted on the 'trustworthiness' of scientists and experts[92]. Contrary to genuinely identifying with the left, Duterte was classified as being a cultural populist who took the pandemic seriously, following many science-based strategies (vaccination, masking, etc.), but implementing these through illiberal means (hard lockdowns, "shoot-to-kill" violators of health standards)[93]. The term “left” was also used by the Duterte administration to refer to communists, which have been tagged by the government as terrorists. It is therefore possible that his supporters would rate themselves more towards the right and conservative ends of these spectra.

In Slovakia, higher trust levels among right-leaning people may be due to an affinity of right-leaning positions and technocracy in domestic politics[94].

In Taiwan, the semantic ambiguity of the term “science” may have caused the positive relationship of right-leaning views and trust a role, as the Chinese translation of “science” includes technology and engineering (which but are often viewed favourably by right-leaning individuals) but excludes the humanities (which are often perceived more favourably by left-leaning individuals). Further, around a third of the participants (33.9%) did not answer the left-right question as this political concept is not locally used. Thus, people might not fully understand the meaning of the response options.

In Georgia, and post-Soviet countries more broadly, the left-/right-leaning political distinction might not be very meaningful to people which is why this finding should be interpreted with caution.

Fig. S13. Average levels of trust in scientists plotted against Gini index across countries.

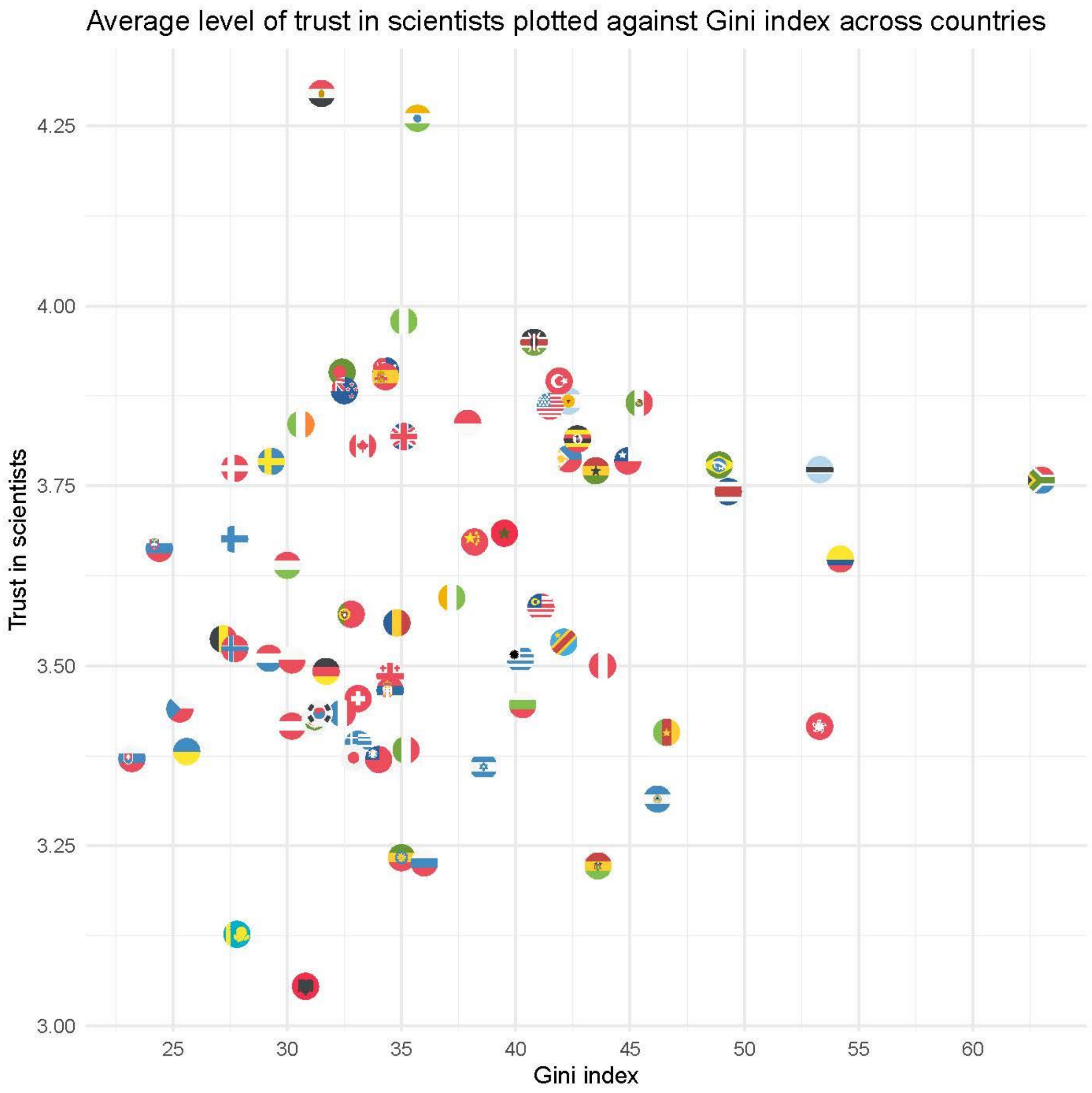

Table S3. Weighted linear multilevel regression predicting trust in scientists (random intercepts across countries) including interaction effects of Gini index and Corruption Perceptions Index as well as Gini index and Overrepresentation of urban population

	**Trust in scientists**					
Predictors	*Beta*	*SE*	*CI*	*t*	*p*	*df*
Intercept	3.590	0.032	3.525 – 3.655	111.914	**<.001**	38.787
Gender (male)	-0.024	0.003	-0.029 – -0.019	-9.411	**<.001**	41568.007
Age	0.007	0.002	0.003 – 0.012	3.165	**.002**	41581.428
Education (tertiary)	-0.014	0.003	-0.019 – -0.009	-5.367	**<.001**	41604.345
Income	0.005	0.003	-0.000 – 0.010	1.938	.053	41566.986
Residence place (urban)	0.014	0.002	0.009 – 0.019	5.678	**<.001**	41566.486
Political orientation (right)	0.008	0.003	0.003 – 0.014	2.824	**.005**	41567.304
Political orientation (conservative)	-0.009	0.003	-0.015 – -0.003	-2.997	**.003**	41566.759
Religiosity	0.005	0.003	0.045 – 0.055	19.267	**<.001**	41566.764
Social dominance orientation	-0.015	0.003	-0.020 – -0.010	-5.727	**<.001**	41568.885
Science-related populist attitudes	-0.021	0.003	-0.026 – -0.016	-8.035	**<.001**	41566.812
Perceived benefit of science	0.152	0.003	0.146 – 0.158	51.502	**<.001**	41567.473
Willingness to be vulnerable to science	0.186	0.003	0.180 – 0.192	61.654	**<.001**	41568.724
Trust in the scientific method	0.187	0.003	0.181 – 0.193	62.053	**<.001**	41567.251
GDP per capita	-0.013	0.053	-0.120 – 0.093	-0.254	.801	38.171
Gvt expenditure on education (% of GDP)	0.028	0.036	-0.044 – 0.101	0.787	.436	39.529
Gini index	0.059	0.034	-0.010 – 0.128	1.726	.092	38.713
Science literacy (PISA)	0.008	0.038	-0.069 – 0.084	0.203	.840	41.182
Academic freedom	-0.024	0.039	-0.102 – 0.054	-0.622	.538	38.912
Degree of populism in politics	-0.001	0.035	-0.072 – 0.070	-0.034	.973	39.389
Perceived level of corruption	-0.101	0.077	-0.256 – 0.054	-1.323	.194	38.319
Overrepresentation of urban population in sample	0.016	0.051	-0.088 – 0.120	0.312	.757	39.214
Gini index x Perceived level of corruption	0.114	0.044	0.026 – 0.202	2.611	**.013**	38.452
Gini index x Overrepresentation of urban population in sample	-0.065	0.063	-0.193 – 0.063	-1.026	.311	38.409
**Random Effects**						
σ^2						0.16
τ_{00}						0.04
ICC						0.19
N						51
Observations						41629
Marginal R^2 / Conditional R^2	0.462 / 0.562					
AIC						76590.573

Note: Significant testing based on two-sided t tests. The Corruption Perceptions Index was retrieved from Transparency International (2018) and reversed so that higher values represent higher perceived corruption. To estimate the overrepresentation of urban population in our sample, we calculated a difference score for each country with the actual difference in urban and rural populations according to data from the World Bank and the difference in urban and rural populations in our sample. Larger values therefore indicate oversampling of urban populations in our sample.

Fig. S14. Comparison of rankings of trust in scientists (TISP data) and trust in the national government (Global Wellcome Monitor data).

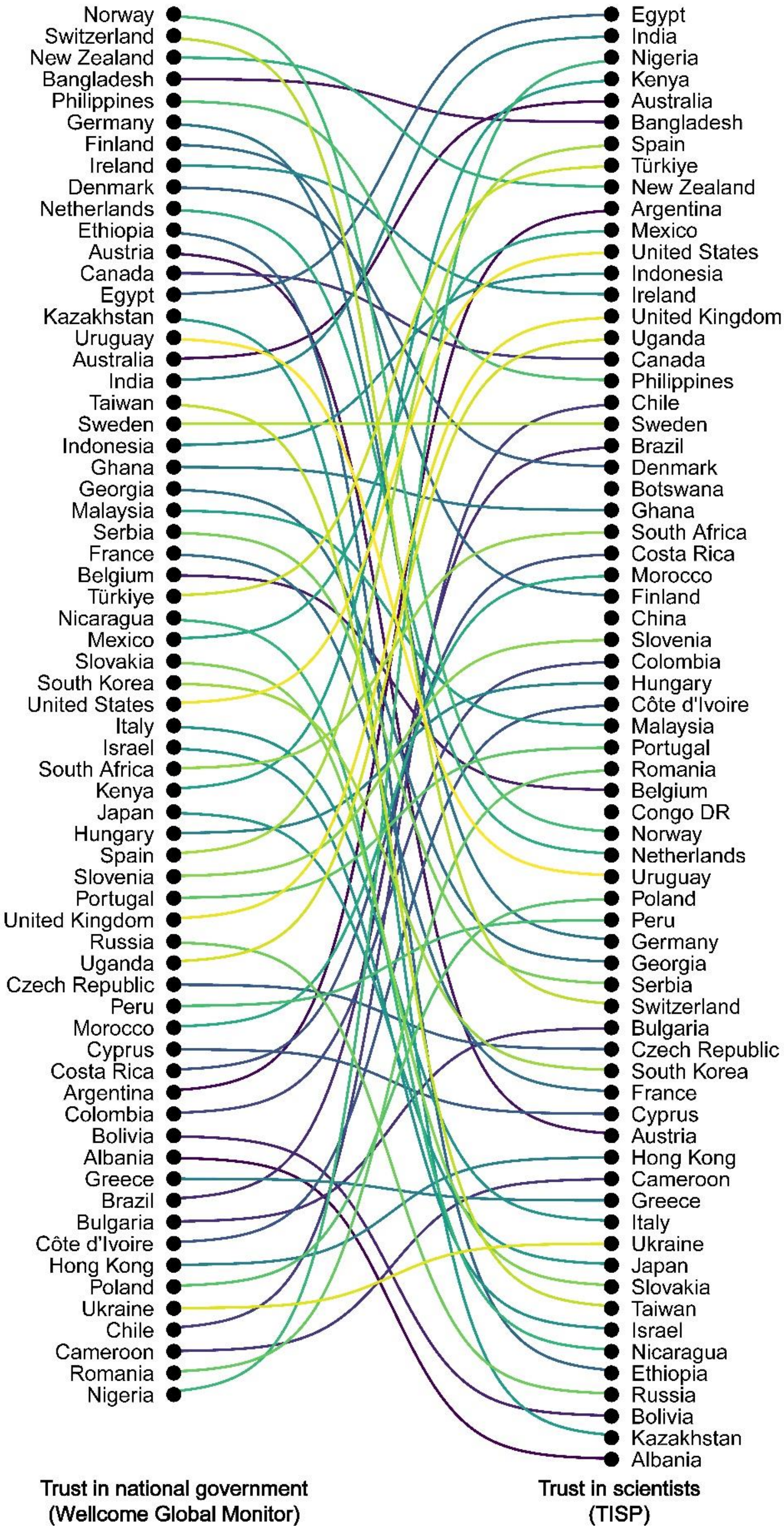

Fig. S15. Weighted means and standard errors of normative perceptions of scientists' engagement across countries

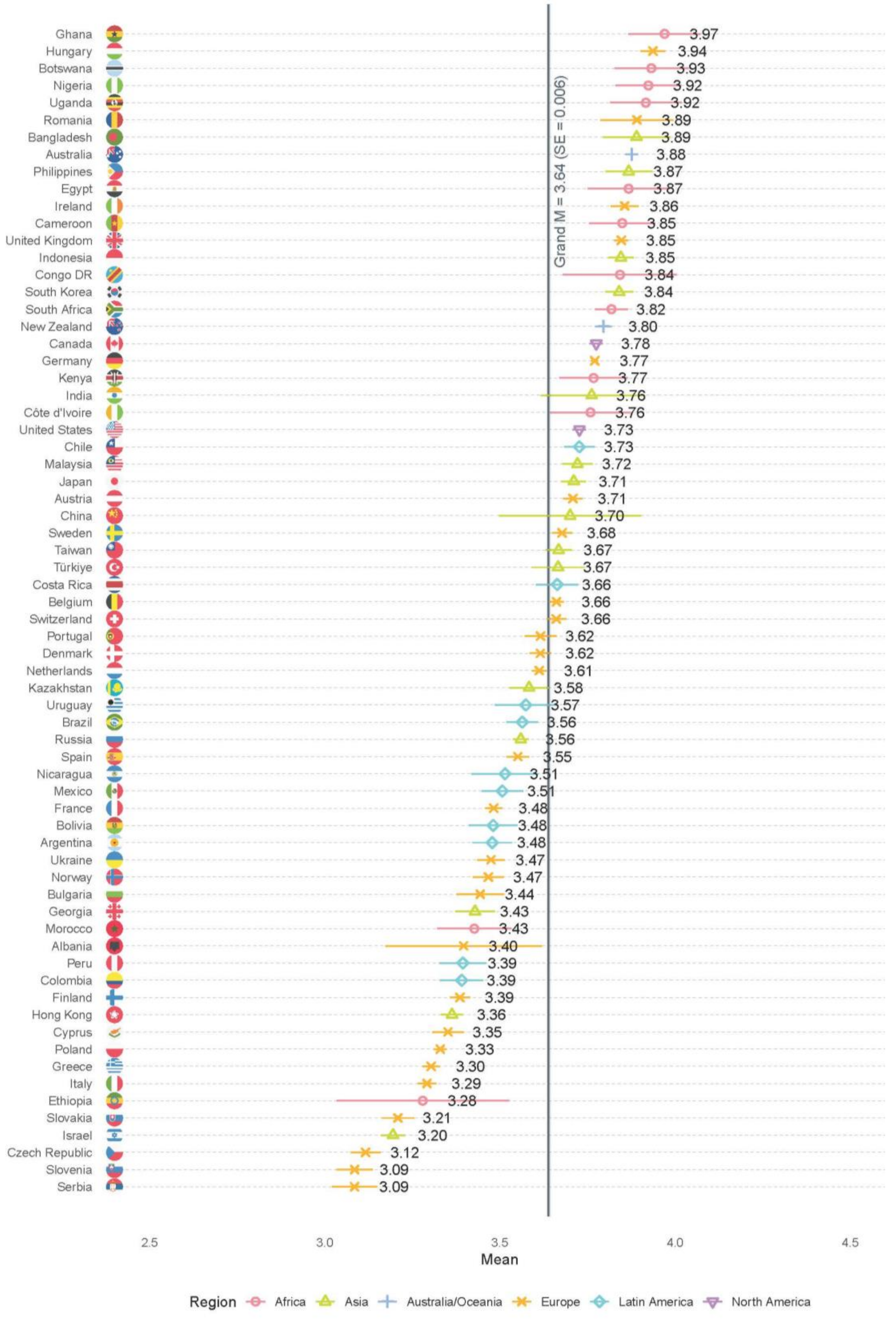

Note: Error bars means +/- standard error. Vertical line indicates the grand mean. Total N = 69,510. Country Ns range between 312 and 8,011 (see table S20 for N across countries).

Table S4. Weighted linear multilevel regression explaining normative perceptions of science (random intercepts across countries).

	Normative perceptions of science					
Predictors	*Beta*	*SE*	*CI*	*t*	*p*	*df*
Intercept	3.685	0.029	3.626 – 3.743	124.927	**<.001**	66.701
Trust in scientists	0.262	0.015	0.232 – 0.291	17.857	**<.001**	48.473
Science-related populist attitudes	0.091	0.012	0.067 – 0.115	7.631	**<.001**	43.641
Gender (male)	0.003	0.003	-0.003 – 0.010	0.962	.336	47534.96
Age	-0.027	0.003	-0.033 – -0.020	-8.426	**<.001**	47602.09
Education (tertiary)	0.037	0.003	0.031 – 0.044	10.74	**<.001**	46780.9
Income	0.007	0.003	0.001 – 0.014	2.136	**.033**	47538.03
Residence place (urban)	0.015	0.003	0.009 – 0.022	4.536	**<.001**	47515.59
Political orientation (right)	-0.02	0.004	-0.028 – -0.012	-4.914	**<.001**	47562.61
Political orientation (conservative)	-0.032	0.004	-0.040 – -0.024	-7.91	**<.001**	47550.38
Religiosity	0.008	0.004	0.001 – 0.015	2.352	**.019**	47564.3
Social dominance orientation	-0.084	0.004	-0.091 – -0.077	-23.528	**<.001**	47547.65
**Random Effects**						
σ^2	0.31					
τ_{00}	0.05					
τ_{11}	0.01					
τ_{11}	0.01					
ρ_{01}	-0.11					
	-0.46					
ICC	0.19					
N COUNTRY_NAME	67					
Observations	47646					
Marginal R^2 / Conditional R^2	0.179 / 0.332					
AIC	132786.797					

Note: Significant testing based on two-sided t tests. AIC = Akaike information criterion, ICC = Intraclass Correlation Coefficient. σ^2 = within-country (residual) variance. τ_{00} = between-country variance (variation between individual intercepts and average intercept). τ_{11} = random slope variance (variation between individual slopes and average slope). ρ_{01} = random intercept-slope correlation.

Fig. S16. Random effects of weighted regression model explaining the relationship of trust in scientists and normative perceptions of science in society and policymaking across countries.

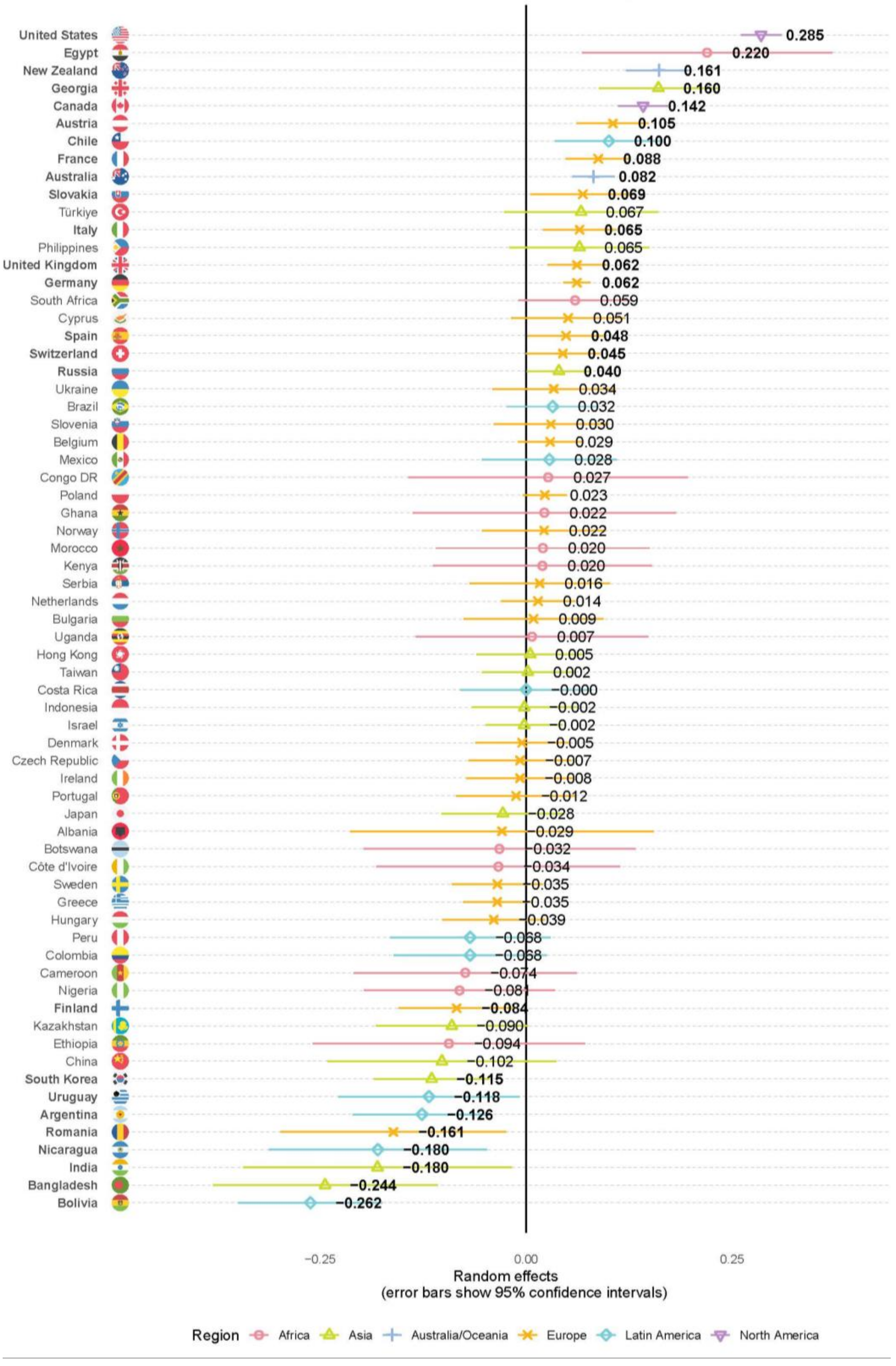

Note: Dots indicate point estimates of random effects, horizontal lines indicate 95% confidence intervals based on two-sided t tests. Effects significant at $p < .05$ are printed in bold. Total $N = 47{,}646$. Country *N*s range between 171 and 5,918 (see table S16 for N across countries).

Note on analyses on the discrepancy between perceived and desired research priorities.

The preregistered analyses treated the discrepancy between perceived and desired priorities as the dependent variable and trust in scientists as an independent variable (Table S5). We ran an exploratory analysis with trust in scientists as the dependent variable and the discrepancy scores as independent variables. The results of these exploratory analyses are reported in the main text and Table S6.

Table S5. Weighted linear multilevel regressions explaining whether perceived priorities exceed desired priorities (random effects of trust in scientists and science-related populist attitudes across countries)

	Improve public health						Solve energy problems					
Predictors	*Beta*	*SE*	*CI*	*t*	*p*	*df*	*Beta*	*SE*	*CI*	*t*	*p*	*df*
Intercept	-0.647	0.04	-0.727 – -0.568	-16.352	**<.001**	48.809	-0.527	0.035	-0.598 – -0.456	-14.968	**<.001**	45.265
Trust in scientists	0.304	0.017	0.270 – 0.338	18.035	**<.001**	50.476	0.218	0.014	0.190 – 0.246	15.733	**<.001**	54.188
Science-related populist attitudes	-0.027	0.01	-0.046 – -0.007	-2.768	**.008**	40.707	-0.001	0.01	-0.023 – 0.020	-0.125	.901	37.882
Gender (male)	0.052	0.005	0.042 – 0.062	10.125	**<.001**	41532	0.01	0.005	-0.001 – 0.020	1.862	.063	41531.1
Age	-0.048	0.005	-0.057 – -0.039	-10.115	**<.001**	41106	-0.018	0.005	-0.027 – -0.008	-3.681	**<.001**	41298.4
Education (tertiary)	0.006	0.005	-0.004 – 0.017	1.238	.216	41057	-0.01	0.005	-0.021 – 0.001	-1.85	.064	40817.5
Income	-0.01	0.005	-0.020 – 0.000	-1.941	.052	41547	0.001	0.005	-0.010 – 0.011	0.161	.872	41528.4
Residence place (urban)	-0.006	0.005	-0.016 – 0.004	-1.143	.253	41548	-0.013	0.005	-0.023 – -0.003	-2.536	**.011**	41528.8
Political orientation (right)	0.032	0.006	0.020 – 0.044	5.233	**<.001**	41460	0.045	0.006	0.033 – 0.057	7.208	**<.001**	41406
Political orientation (conservative)	-0.02	0.006	-0.032 – -0.008	-3.286	**.001**	41533	0.016	0.006	0.003 – 0.028	2.513	**.012**	41520.3
Religiosity	0.005	0.005	-0.005 – 0.016	0.997	.319	41492	0.022	0.005	0.012 – 0.033	4.124	**<.001**	41480.9
Social dominance orientation	0.114	0.005	0.104 – 0.125	21.17	**<.001**	41057	0.105	0.006	0.094 – 0.116	18.871	**<.001**	41118.4
Perceived benefit of science	0.128	0.006	0.116 – 0.140	20.698	**<.001**	41526	0.071	0.006	0.059 – 0.084	11.231	**<.001**	41524.4
Willingness to be vulnerable to science	-0.069	0.006	-0.082 – -0.057	-10.769	**<.001**	41410	-0.06	0.007	-0.073 – -0.047	-9.069	**<.001**	41393.4
Trust in the scientific method	-0.014	0.006	-0.027 – -0.002	-2.198	**.028**	41542	-0.016	0.007	-0.029 – -0.004	-2.509	**.012**	41527
GDP per capita	0.036	0.039	-0.042 – 0.115	0.94	.353	38.378	-0.019	0.038	-0.096 – 0.058	-0.493	.625	38.687
Gvt expenditure on education (% of GDP)	0.035	0.034	-0.035 – 0.104	1.007	.320	40.598	0.097	0.034	0.029 – 0.165	2.874	**.006**	41.151
Gini index	0.063	0.032	-0.002 – 0.127	1.951	.057	45.456	0.009	0.032	-0.054 – 0.072	0.286	.776	45.937

Science literacy (PISA)	0.126	0.036	0.053 – 0.199	3.472	**.001**	51.253	0.053	0.036	-0.019 – 0.124	1.482	.145	51.532
Academic freedom	-0.065	0.034	-0.134 – 0.004	-1.895	.065	44.981	-0.044	0.034	-0.112 – 0.023	-1.318	.194	45.291
Degree of populism in politics	-0.009	0.031	-0.071 – 0.054	-0.272	.787	45.029	-0.004	0.03	-0.065 – 0.058	-0.127	.900	44.511
**Random Effects**												
σ^2	0.67						0.71					
τ_{00}	0.07 COUNTRY_NAME						0.05 COUNTRY_NAME					
τ_{11}	0.01 COUNTRY_NAME.TRUST_SCI_m_z_grp						0.01 COUNTRY_NAME.TRUST_SCI_m_z_grp					
	0.00 COUNTRY_NAME.SCIPOP_goertz_z_grp						0.00 COUNTRY_NAME.SCIPOP_goertz_z_grp					
ρ_{01}	-0.78						-0.68					
	0.28						0.26					
ICC	0.11						0.08					
N	51 COUNTRY_NAME						51 COUNTRY_NAME					
Observations	41610						41593					
Marginal R^2 / Conditional R^2	0.161 / 0.250						0.083 / 0.156					
AIC	135116.454						137195.686					

Table S5. Continued

	**Reduce poverty**						**Develop defense and military technology**					
Predictors	*Beta*	*SE*	*CI*	*t*	*p*	*df*	*Beta*	*SE*	*CI*	*t*	*p*	*df*
Intercept	-1.082	0.061	-1.204 – -0.960	-17.811	**<.001**	48.575	0.881	0.061	0.758 – 1.003	14.481	**<.001**	47.128
Trust in scientists	0.365	0.021	0.323 – 0.408	17.341	**<.001**	52.081	-0.254	0.019	-0.293 – -0.215	-13.01	**<.001**	54.508
Science-related populist attitudes	0.018	0.015	-0.012 – 0.049	1.208	.234	39.253	-0.106	0.013	-0.132 – -0.081	-8.507	**<.001**	24.089
Gender (male)	0.046	0.006	0.033 – 0.058	7.188	**<.001**	41512	0.136	0.007	0.122 – 0.150	19.102	**<.001**	41492.6
Age	-0.055	0.006	-0.067 – -0.044	-9.388	**<.001**	41466	-0.034	0.007	-0.047 – -0.021	-5.121	**<.001**	40129.3
Education (tertiary)	0.011	0.007	-0.002 – 0.024	1.705	.088	41449	0.064	0.007	0.049 – 0.078	8.709	**<.001**	41370.2
Income	-0.007	0.006	-0.019 – 0.006	-1.006	.314	41514	0.065	0.007	0.050 – 0.079	8.878	**<.001**	41517.7
Residence place (urban)	-0.001	0.006	-0.013 – 0.011	-0.145	.885	41511	0.006	0.007	-0.008 – 0.020	0.874	.382	41517.9
Political orientation (right)	0.104	0.008	0.089 – 0.119	13.711	**<.001**	41512	-0.154	0.009	-0.170 – -0.137	-18.054	**<.001**	40998.9

Political orientation (conservative)	0.003	0.008	-0.012 – 0.018	0.422	.673	41519	-0.045	0.009	-0.062 – -0.028	-5.27	**<.001**	41450.2
Religiosity	0.003	0.007	-0.010 – 0.016	0.485	.628	41525	-0.078	0.007	-0.093 – -0.064	-10.578	**<.001**	41249.3
Social dominance orientation	0.256	0.007	0.243 – 0.269	38.014	**<.001**	41407	-0.145	0.008	-0.160 – -0.130	-19.231	**<.001**	39818.5
Perceived benefit of science	0.052	0.008	0.037 – 0.067	6.783	**<.001**	41522	0.071	0.009	0.054 – 0.088	8.259	**<.001**	41471.6
Willingness to be vulnerable to science	-0.106	0.008	-0.122 – -0.090	-13.261	**<.001**	41494	0.117	0.009	0.100 – 0.135	13.086	**<.001**	41304.8
Trust in the scientific method	-0.03	0.008	-0.046 – -0.015	-3.802	**<.001**	41514	0.059	0.009	0.041 – 0.076	6.56	**<.001**	41519.6
GDP per capita	-0.025	0.066	-0.158 – 0.108	-0.386	.702	41.419	-0.039	0.071	-0.183 – 0.105	-0.543	.590	40.964
Gvt expenditure on education (% of GDP)	0.129	0.058	0.013 – 0.246	2.233	**.031**	43.578	-0.182	0.062	-0.307 – -0.056	-2.912	**.006**	42.476
Gini index	0.071	0.053	-0.037 – 0.178	1.323	.192	46.105	0.009	0.058	-0.107 – 0.125	0.162	.872	44.697
Science literacy (PISA)	0.156	0.06	0.037 – 0.276	2.62	**.012**	51.3	-0.064	0.064	-0.192 – 0.064	-1.001	.322	48.994
Academic freedom	-0.064	0.057	-0.180 – 0.051	-1.125	.266	46.417	0.128	0.062	0.004 – 0.252	2.079	**.043**	45.033
Degree of populism in politics	0.068	0.052	-0.036 – 0.173	1.311	.196	47.319	-0.133	0.056	-0.245 – -0.020	-2.376	**.022**	45.606
**Random Effects**												
σ^2	1.04						1.31					
τ_{00}	0.17 COUNTRY_NAME						0.16 COUNTRY_NAME					
τ_{11}	0.02 COUNTRY_NAME.TRUST_SCI_m_z_grp						0.01 COUNTRY_NAME.TRUST_SCI_m_z_grp					
	0.01 COUNTRY_NAME.SCIPOP_goertz_z_grp						0.00 COUNTRY_NAME.SCIPOP_goertz_z_grp					
ρ_{01}	-0.68						-0.57					
	0.19						-0.48					
ICC	0.15						0.12					
N	51 COUNTRY_NAME						51 COUNTRY_NAME					
Observations	41586						41586					
Marginal R^2 / Conditional R^2	0.155 / 0.282						0.132 / 0.234					
AIC	153149.198						162702.628					

Note: Significant testing based on two-sided t tests. AIC = Akaike information criterion, ICC = Intraclass Correlation Coefficient. σ^2 = within-country (residual) variance. τ_{00} = between-country variance (variation between individual intercepts and average intercept). τ_{11} = random slope variance (variation between individual slopes and average slope). ρ_{01} = random intercept-slope correlation.

Table S6. Weighted linear multilevel regressions predicting trust in scientists with perceptions that science's efforts to tackle four goals (i.e., perceived priorities) exceed expectations for scientists to prioritize these goals (i.e., desired priorities; random effects of perceived-desired discrepancy scores and science-related populist attitudes across countries).

	Trust in scientists					
Predictors	*Beta*	*SE*	*CI*	*t*	*p*	*df*
Intercept	3.611	0.031	3.549 – 3.673	117.379	**<.001**	43.389
Improve public health	0.064	0.007	0.051 – 0.078	9.713	**<.001**	32.91
Solve energy problems	0.024	0.005	0.013 – 0.035	4.523	**<.001**	30.71
Reduce poverty	0.064	0.007	0.050 – 0.078	9.499	**<.001**	31.692
Develop defense and military technology	-0.052	0.006	-0.063 – -0.040	-9.194	**<.001**	34.988
Science-related populist attitudes	-0.022	0.007	-0.036 – -0.008	-3.134	**.003**	39.334
Gender (male)	-0.022	0.002	-0.027 – -0.017	-9.116	**<.001**	41402.87
Age	0.012	0.002	0.008 – 0.017	5.506	**<.001**	41407.11
Education (tertiary)	-0.011	0.002	-0.016 – -0.006	-4.5	**<.001**	41425.01
Income	0.007	0.002	0.002 – 0.012	2.963	**.003**	41401.14
Residence place (urban)	0.012	0.002	0.008 – 0.017	5.218	**<.001**	41401.07
Political orientation (right)	-0.007	0.003	-0.012 – -0.001	-2.307	**.021**	41430.84
Political orientation (conservative)	-0.008	0.003	-0.013 – -0.002	-2.611	**.009**	41422.25
Religiosity	0.041	0.003	0.036 – 0.046	16.406	**<.001**	41411.34
Social dominance orientation	-0.043	0.003	-0.048 – -0.038	-16.496	**<.001**	41383.65
Perceived benefit of science	0.128	0.003	0.122 – 0.133	44.466	**<.001**	41321.43
Willingness to be vulnerable to science	0.185	0.003	0.179 – 0.191	63.77	**<.001**	41419.08
Trust in the scientific method	0.175	0.003	0.170 – 0.181	60.632	**<.001**	41409.59
GDP per capita	0.025	0.04	-0.055 – 0.105	0.626	.534	42.03
Gvt expenditure on education (% of GDP)	0.047	0.034	-0.022 – 0.117	1.376	.176	43.245
Gini index	0.1	0.032	0.036 – 0.164	3.167	**.003**	43.491
Science literacy (PISA)	0.018	0.034	-0.051 – 0.088	0.529	.600	45.877
Academic freedom	-0.004	0.034	-0.072 – 0.064	-0.113	.911	43.556
Degree of populism in politics	-0.015	0.03	-0.076 – 0.047	-0.479	.634	44.065
**Random Effects**						
σ^2	0.15					
τ_{00}	0.04					
τ_{11}	0					
τ_{11}	0					
τ_{11}	0					
τ_{11}	0					
τ_{11}	0					
ρ_{01}	-0.11					
	-0.08					
	-0.34					
	0.04					
	-0.09					
ICC	0.24					
N COUNTRY_NAME	51					
Observations	41534					
Marginal R^2 / Conditional R^2	0.481 / 0.607					
AIC	72552.404					

Note: Significant testing based on two-sided t tests.

Fig. S17. Weighted means and standard errors of desired priority for developing defence and military technology across countries.

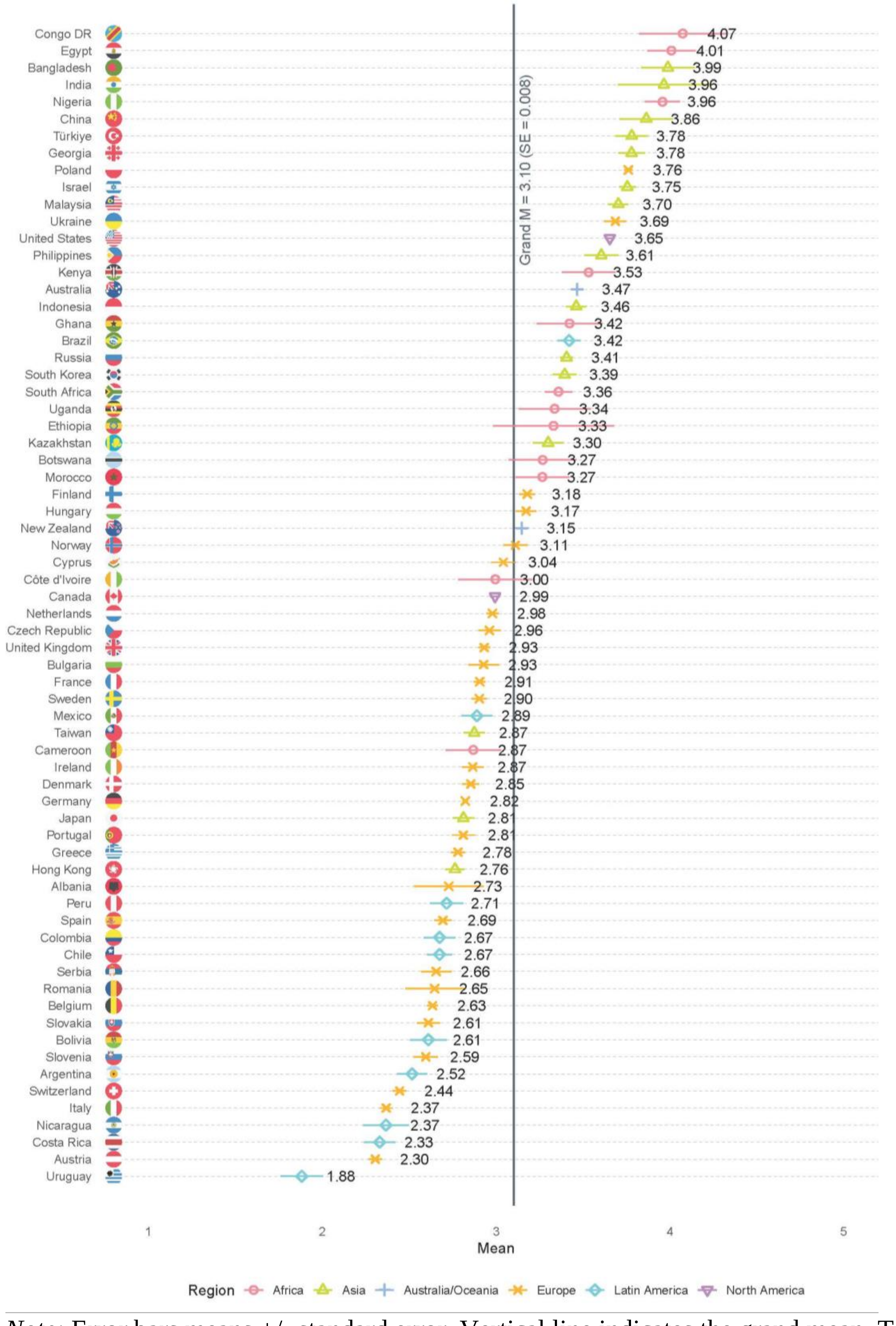

Note: Error bars means +/- standard error. Vertical line indicates the grand mean. Total N = 69,429. Country Ns range between 310 and 8,005 (see table S20 for a detailed overview).

Table S7. Weighted zero-order correlations of trust in scientists with perceived research priorities.

	Trust in scientists	Improve public health	Solve energy problems	Reduce poverty	Develop defence and military technology
Trust in scientists	1				
Improve public health	.47	1			
Solve energy problems	.42	.64	1		
Reduce poverty	.40	.52	.51	1	
Develop defence and military technology	.14	.14	.22	.10	1

Note: Estimates are based on post-stratification weights accounting for unequal selection probabilities across countries, genders, age groups, and education levels. All values significant at $p < .001$ (two-sided t tests).

Table S8. Weighted zero-order correlations of trust in scientists with desired research priorities.

	Trust in scientists	Improve public health	Solve energy problems	Reduce poverty	Develop defence and military technology
Trust in scientists	1				
Improve public health	.21	1			
Solve energy problems	.23	.44	1		
Reduce poverty	.14	.42	.37	1	
Develop defence and military technology	.22	.08	.10	.12	1

Note: Estimates are based on post-stratification weights accounting for unequal selection probabilities across countries, genders, age groups, and education levels. All values significant at $p < .001$ (two-sided t tests).

Sensitivity tests for different trust measures

Our study relies on a multidimensional measure for trust in scientists that is based on established theoretical assumptions and existing empirical analyses[48] suggesting that trust in scientists is a multidimensional construct consisting of four components of trust perceptions, i.e., competence, integrity, benevolence, and openness.

We applied several procedures to ensure that the scale captures these four dimensions and thus accurately reflects the latent construct "trust in scientists" as conceptualized in the relevant literature[48]. We validated the scale in a pretest in the United States (n = 401, October 2022). Then, we confirmed the theoretical assumptions underlying the scale, showing that the 12 items largely map onto the four theoretical dimensions and form four stable factors. Albeit limited cross-country measurement invariance, we find high scale reliability across all countries as well as globally (see the associated publication[38], which provides a detailed description of the TISP dataset as well as psychometric analyses and measurement performance tests of key measures, including the scale measuring trust in scientists).

Other measures used to assess trust in science and scientists include the commonly used 1-item measures asking people to indicate their "confidence that scientists act in the best interests of the public"[39] and measures of trust in the scientific method. These 1-item measures have been criticized as they arguably capture only single aspects of trust in scientists: The confidence measure, for example, primarily addresses people's perceptions of scientists' benevolence, whereas the trust-in-methods measure does not refer to the trustworthiness of scientists but to the reliability of their work. However, these 1-item measures are still commonly used[25] and may be valid and useful alternatives to multi-item measures[95]. Hence, we included the 1-item confidence measure as well as the trust-in-methods measure in the questionnaire and re-ran the analyses underlying Figure 1, Figure 2, and table S2 (see fig. S19-S20 as well as tables S8 and S9). We also provide detailed descriptive statistics (*M, Me, SE, SD*; table S1) as well as zero-order correlations (table S7) of the 12-item trust measure, the confidence measure, the trust-in-methods measure, all 12 individual items measuring trust in scientists and the four dimensions of the 12-item measure. Additionally, we re-ran the analyses underlying Figure 1 for all four-dimension scores as well as the confidence and the trust-in-method measure (fig. S13-S18).

We find that the grand mean of our 12-item trust in scientists measure ($M = 3.62$, $SE < 0.001$) and, as expected, the perceptions of scientists' benevolence measure ($M = 3.55$, $SE = 0.01$) to be very close to the grand mean of the commonly used 1-item measure of confidence in scientists ($M = 3.57$, $SE = 0.01$), and find some differences across countries which is plausible given that the "confidence in scientists to act in the public interest" measure only captures one component of trust in scientists (e.g., benevolence). The differences in means of the four components of the 12-item trust in scientists measure (i.e., competence, benevolence, integrity, openness) across countries (fig. S13-S16), further highlight the importance of distinguishing between different dimensions of trustworthiness when assessing perceptions of scientists. Given this evidence, as well its high alpha and omega reliability and the results of our factor analyses, we conclude that our trust in scientists measure is a valid measure to assess trust in scientists given that it reflects the results of the 1-item measure but additionally adds nuance to understand underlying trustworthiness dimensions.

Table S9. Weighted zero-order correlations of trust index with other trust measures and dimension scores

	Trust in scientists (12-item index)	Confidence that scientists act in the best interests of the public	Trust in the scientific method
**Trust in scientists (12-item index)**	1		
**Confidence that scientists act in the best interests of the public**	0.71	1	
**Trust in the scientific method**	0.47	0.48	1
**Competence**	0.81	0.54	0.44
**Integrity**	0.91	0.66	0.43
**Benevolence**	0.91	0.66	0.41
**Openness**	0.88	0.63	0.39
How expert or inexpert are most scientists?	0.67	0.44	0.34
How intelligent or unintelligent are most scientists?	0.66	0.43	0.38
How qualified or unqualified are most scientists when it comes to conducting high-quality research?	0.71	0.47	0.38
How honest or dishonest are most scientists?	0.76	0.55	0.36
How ethical or unethical are most scientists?	0.77	0.54	0.36
How sincere or insincere are most scientists?	0.81	0.60	0.38
How concerned or not concerned are most scientists about people's well-being?	0.76	0.55	0.34
How eager or uneager are most scientists to improve others' lives?	0.78	0.57	0.37
How considerate or inconsiderate are most scientists of others' interests?	0.78	0.57	0.35
How open are most scientists to feedback?	0.73	0.50	0.32
How willing or unwilling are most scientists to be transparent?	0.77	0.55	0.35
How much or little attention do scientists pay to others' views?	0.73	0.54	0.33

Note: Estimates are based on post-stratification weights accounting for unequal selection probabilities across countries, genders, age groups, and education levels. All values significant at $p < .001$ (two-sided t tests).

Table S10. Weighted linear multilevel regression explaining confidence in scientists to act in public interest

	Block 1: Demographic characteristics						Block 2: Ideological views					
Predictors	*Beta*	*SE*	*CI*	*t*	*p*	*df*	*Beta*	*SE*	*CI*	*t*	*p*	*df*
Intercept	3.636	0.042	3.552 – 3.720	86.185	**<.001**	63.58	3.679	0.04	3.599 – 3.760	91.548	**<.001**	60.67
Gender (male)	0.006	0.004	-0.002 – 0.014	1.436	.151	64373.73	0.010	0.005	0.001 – 0.018	2.103	**.035**	47589.03
Age	0.015	0.004	0.008 – 0.022	4.009	**<.001**	64415.89	-0.005	0.004	-0.013 – 0.004	-1.115	.265	47632.04
Education (tertiary)	0.045	0.004	0.036 – 0.053	10.531	**<.001**	63667.53	0.042	0.005	0.033 – 0.051	8.969	**<.001**	46916.14
Income	0.037	0.004	0.029 – 0.045	9.148	**<.001**	64381.78	0.023	0.005	0.014 – 0.032	5.013	**<.001**	47593.04
Residence place (urban)	0.038	0.004	0.030 – 0.046	9.742	**<.001**	64369.88	0.039	0.004	0.030 – 0.048	8.793	**<.001**	47587.87
Political orientation (right)							-0.005	0.005	-0.015 – 0.006	-0.914	.361	47591.98
Political orientation (conservative)							-0.042	0.005	-0.053 – -0.032	-7.842	**<.001**	47595.47
Religiosity							0.056	0.005	0.046 – 0.065	11.943	**<.001**	47590.72
Social dominance orientation							-0.113	0.005	-0.122 – -0.103	-24.179	**<.001**	47597.5
Science-related populist attitudes												
Perceived benefit of science												
Willingness to be vulnerable to science												
Trust in the scientific method												
GDP per capita												
Gvt expenditure on education (% of GDP)												
Gini index												

Science literacy (PISA)		
Academic freedom		
Degree of populism in politics		
**Random Effects**		
σ^2	0.61	0.57
τ_{00}	0.12 COUNTRY_NAME	0.10 COUNTRY_NAME
ICC	0.16	0.15
N	68 COUNTRY_NAME	67 COUNTRY_NAME
Observations	64442	47657
Marginal R^2 / Conditional R^2	0.008 / 0.166	0.032 / 0.179
AIC	223462.948	160765.649

Table S10. Continued

	**Block 3: Attitudes to science**						**Block 4: Country indicators**					
Predictors	*Beta*	*SE*	*CI*	*t*	*p*	*df*	*Beta*	*SE*	*CI*	*t*	*p*	*df*
Intercept	3.595	0.039	3.517 – 3.672	92.762	**<.001**	60.936	3.536	0.037	3.461 – 3.611	95.036	**<.001**	41.94
Gender (male)	-0.010	0.004	-0.017 – -0.003	-2.712	**.007**	46627.87	-0.012	0.004	-0.019 – -0.004	-2.983	**.003**	41565.18
Age	-0.020	0.003	-0.027 – -0.013	-5.787	**<.001**	46663.34	-0.02	0.004	-0.027 – -0.013	-5.482	**<.001**	41584.54
Education (tertiary)	-0.024	0.004	-0.031 – -0.016	-6.120	**<.001**	46333.10	-0.024	0.004	-0.032 – -0.016	-5.907	**<.001**	41587.7
Income	-0.006	0.004	-0.013 – 0.002	-1.514	.130	46631.10	-0.004	0.004	-0.011 – 0.004	-0.895	.371	41563.41
Residence place (urban)	0.015	0.004	0.008 – 0.022	4.072	**<.001**	46627.18	0.016	0.004	0.009 – 0.024	4.245	**<.001**	41562.48
Political orientation (right)	-0.002	0.004	-0.010 – 0.007	-0.384	.701	46629.92	-0.002	0.005	-0.012 – 0.007	-0.525	.600	41564.22
Political orientation (conservative)	-0.002	0.004	-0.011 – 0.006	-0.515	.607	46633.20	-0.006	0.005	-0.015 – 0.003	-1.242	.214	41563.12
Religiosity	0.06	0.004	0.053 – 0.068	15.554	**<.001**	46629.22	0.06	0.004	0.052 – 0.068	14.681	**<.001**	41563.73

Social dominance orientation	0.013	0.004	0.005 – 0.021	3.28	**.001**	46634.39	0.014	0.004	0.006 – 0.022	3.342	**.001**	41565.78
Science-related populist attitudes	-0.063	0.004	-0.070 – -0.055	-16.048	<**.001**	46627.18	-0.062	0.004	-0.070 – -0.054	-14.993	<**.001**	41563.42
Perceived benefit of science	0.212	0.004	0.203 – 0.221	48.423	<**.001**	46634.55	0.21	0.005	0.201 – 0.219	45.496	<**.001**	41565.14
Willingness to be vulnerable to science	0.226	0.004	0.217 – 0.234	50.299	<**.001**	46631.62	0.227	0.005	0.217 – 0.236	47.897	<**.001**	41566.21
Trust in the scientific method	0.302	0.004	0.293 – 0.311	67.521	<**.001**	46631.37	0.300	0.005	0.290 – 0.309	63.409	<**.001**	41563.6
GDP per capita							0.067	0.051	-0.035 – 0.169	1.322	.193	41.283
Gvt expenditure on education (% of GDP)							0.035	0.044	-0.053 – 0.124	0.808	.423	42.379
Gini index							0.016	0.04	-0.064 – 0.097	0.410	.684	42.187
Science literacy (PISA)							-0.052	0.044	-0.140 – 0.036	-1.183	.243	44.945
Academic freedom							-0.046	0.043	-0.132 – 0.041	-1.064	.293	42.478
Degree of populism in politics							-0.049	0.039	-0.127 – 0.029	-1.255	.216	43.017
**Random Effects**												
σ^2	0.37						0.4					
τ_{00}	0.09 COUNTRY_NAME						0.06 COUNTRY_NAME					
ICC	0.2						0.13					
N	66 COUNTRY_NAME						51 COUNTRY_NAME					
Observations	46701						41625					
Marginal R^2 / Conditional R^2	0.403 / 0.523						0.411 / 0.486					
AIC	138635.961						113895.986					

Note: Significant testing based on two-sided t tests. AIC = Akaike information criterion, ICC = Intraclass Correlation Coefficient. σ^2 = within-country (residual) variance. τ_{00} = between-country variance (variation between individual intercepts and average intercept).

Table S11. Weighted linear multilevel regression explaining trust in the scientific method (random intercepts across countries)

	**Block 1: Demographic characteristics**						**Block 2: Ideological views**					
Predictors	*Beta*	*SE*	*CI*	*t*	*p*	*df*	*Beta*	*SE*	*CI*	*t*	*p*	*df*
Intercept	4.215	0.032	4.151 – 4.278	132.635	**<.001**	65.09	4.254	0.032	4.190 – 4.317	134.251	**<.001**	62.014
Gender (male)	0.053	0.004	0.045 – 0.060	14.235	**<.001**	64391.01	0.059	0.004	0.051 – 0.068	14.269	**<.001**	47600.32
Age	0.03	0.003	0.023 – 0.037	8.744	**<.001**	64440.69	0.021	0.004	0.013 – 0.028	5.43	**<.001**	47646.72
Education (tertiary)	0.067	0.004	0.059 – 0.075	17.044	**<.001**	62557.91	0.065	0.004	0.056 – 0.073	15.097	**<.001**	46271.49
Income	0.043	0.004	0.036 – 0.050	11.48	**<.001**	64401	0.03	0.004	0.021 – 0.038	6.944	**<.001**	47604.36
Residence place (urban)	0.037	0.004	0.030 – 0.044	10.178	**<.001**	64385.6	0.041	0.004	0.033 – 0.049	10.101	**<.001**	47598.59
Political orientation (right)							0.014	0.005	0.005 – 0.024	2.914	**.004**	47603.75
Political orientation (conservative)							-0.04	0.005	-0.049 – -0.030	-8.007	**<.001**	47608.12
Religiosity							-0.027	0.004	-0.036 – -0.019	-6.352	**<.001**	47602.24
Social dominance orientation							-0.144	0.004	-0.152 – -0.136	-33.628	**<.001**	47609.83
Science-related populist attitudes												
Perceived benefit of science												
Willingness to be vulnerable to science												
GDP per capita												
Gvt expenditure on education (% of GDP)												

Gini index		
Science literacy (PISA)		
Academic freedom		
Degree of populism in politics		
**Random Effects**		
σ^2	0.53	0.48
τ_{00}	0.06 $_{COUNTRY\_NAME}$	0.06 $_{COUNTRY\_NAME}$
ICC	0.11	0.11
N	68 $_{COUNTRY\_NAME}$	67 $_{COUNTRY\_NAME}$
Observations	64454	47663
Marginal R^2 / Conditional R^2	0.022 / 0.129	0.063 / 0.169
AIC	213890.768	152831.032

Table S11. Continued

	Block 3: Attitudes to science						Block 4: Country-level indicators					
Predictors	*Beta*	*SE*	*CI*	*t*	*p*	*df*	*Beta*	*SE*	*CI*	*t*	*p*	*df*
Intercept	4.182	0.03	4.122 – 4.241	140.106	**<.001**	61.68	4.107	0.027	4.053 – 4.161	152.886	**<.001**	43.01
Gender (male)	0.056	0.004	0.049 – 0.063	15.581	**<.001**	46642.75	0.057	0.004	0.049 – 0.064	14.879	**<.001**	41577.56
Age	0.009	0.003	0.003 – 0.016	2.749	**.006**	46685.65	0.009	0.004	0.002 – 0.016	2.579	**.010**	41601.7
Education (tertiary)	0.014	0.004	0.007 – 0.021	3.711	**<.001**	45662.82	0.015	0.004	0.008 – 0.023	3.915	**<.001**	41468.48
Income	0.008	0.004	0.000 – 0.015	2.073	**.038**	46646.1	0.009	0.004	0.001 – 0.016	2.195	**.028**	41574.49
Residence place (urban)	0.025	0.004	0.018 – 0.032	7.054	**<.001**	46641.42	0.025	0.004	0.018 – 0.033	6.713	**<.001**	41573.16

Political orientation (right)	0.025	0.004	0.017 – 0.034	5.824	**<.001**	46645.55	0.023	0.005	0.014 – 0.032	5.022	**<.001**	41575.76
Political orientation (conservative)	-0.008	0.004	-0.016 – 0.001	-1.795	.073	46650.15	-0.007	0.005	-0.016 – 0.002	-1.516	.129	41574.3
Religiosity	-0.035	0.004	-0.042 – -0.028	-9.295	**<.001**	46644.2	-0.035	0.004	-0.043 – -0.027	-8.856	**<.001**	41574.7
Social dominance orientation	-0.047	0.004	-0.054 – -0.039	-12.284	**<.001**	46651.07	-0.047	0.004	-0.054 – -0.039	-11.493	**<.001**	41577.37
Science-related populist attitudes	-0.057	0.004	-0.065 – -0.050	-15.067	**<.001**	46641.79	-0.057	0.004	-0.065 – -0.049	-14.297	**<.001**	41574.77
Perceived benefit of science	0.217	0.004	0.209 – 0.225	52.677	**<.001**	46652.77	0.218	0.004	0.210 – 0.227	49.982	**<.001**	41577.58
Willingness to be vulnerable to science	0.342	0.004	0.334 – 0.350	84.341	**<.001**	46645.07	0.344	0.004	0.335 – 0.352	80.087	**<.001**	41577.08
GDP per capita							-0.052	0.036	-0.126 – 0.021	-1.44	.157	41.986
Gvt expenditure on education (% of GDP)							0.012	0.032	-0.052 – 0.076	0.392	.697	43.312
Gini index							0.068	0.029	0.009 – 0.126	2.334	**.024**	43.45
Science literacy (PISA)							-0.021	0.032	-0.085 – 0.044	-0.65	.519	47.938
Academic freedom							0.018	0.031	-0.045 – 0.080	0.577	.567	44.008
Degree of populism in politics							0.01	0.028	-0.047 – 0.066	0.343	.733	45.051
**Random Effects**												
σ^2	0.35						0.38					
τ_{00}	0.05 COUNTRY_NAME						0.03 COUNTRY_NAME					
ICC	0.13						0.07					

N	66 COUNTRY_NAME	51 COUNTRY_NAME
Observations	46709	41631
Marginal R^2 / Conditional R^2	0.363 / 0.448	0.371 / 0.417
AIC	135853.157	111665.054

Note: Significant testing based on two-sided t tests. AIC = Akaike information criterion, ICC = Intraclass Correlation Coefficient. σ^2 = within-country (residual) variance. τ_{00} = between-country variance (variation between individual intercepts and average intercept).

Fig. S18. Weighted means and standard errors of competence dimension of trust in scientists across countries

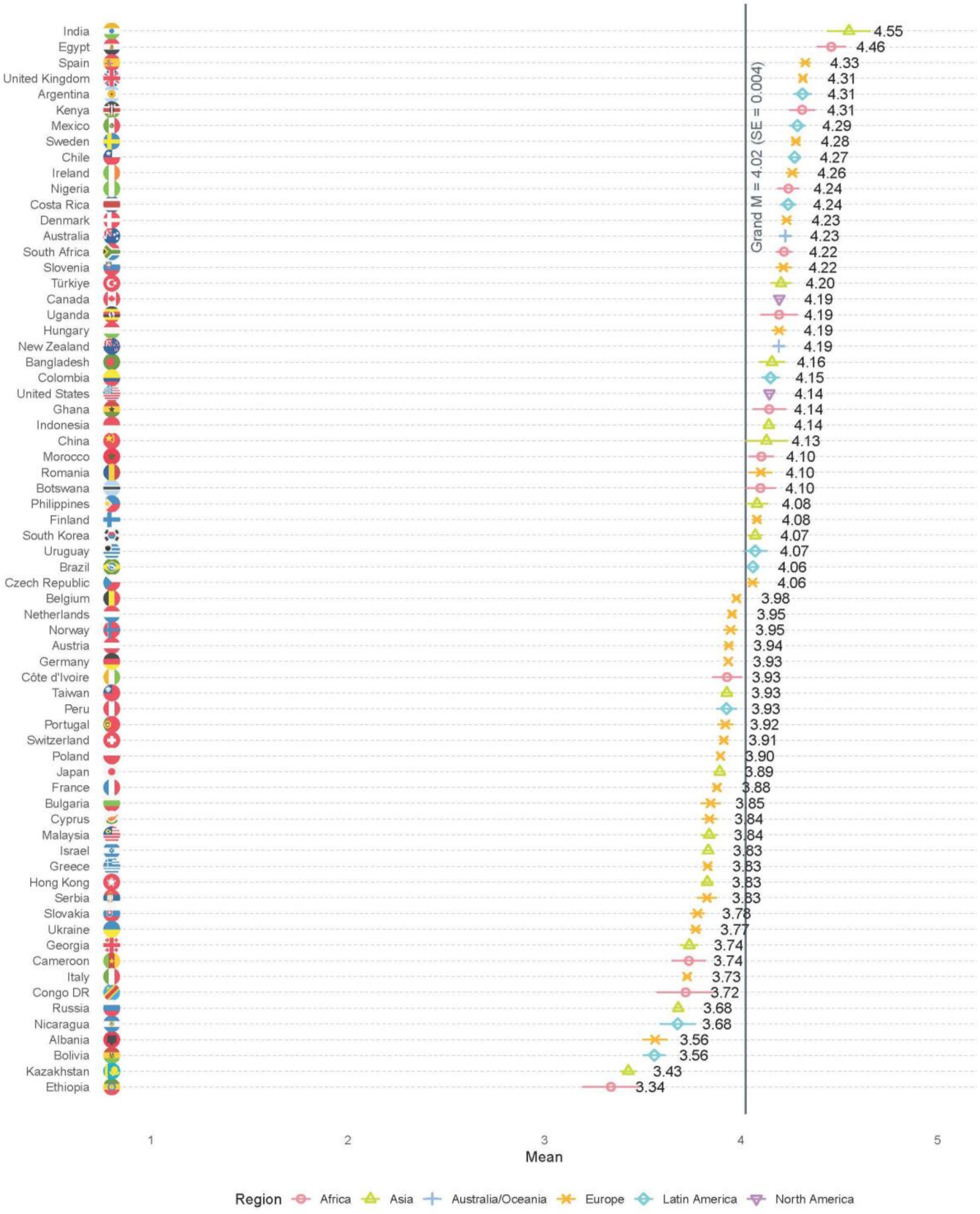

Note: Error bars means +/- standard error. Vertical line indicates the grand mean. Total N = 69,526. Country Ns range between 312 and 8,011 (see table S20 for a detailed overview).

Fig. S19. Weighted means and standard errors of integrity dimension of trust in scientists across countries

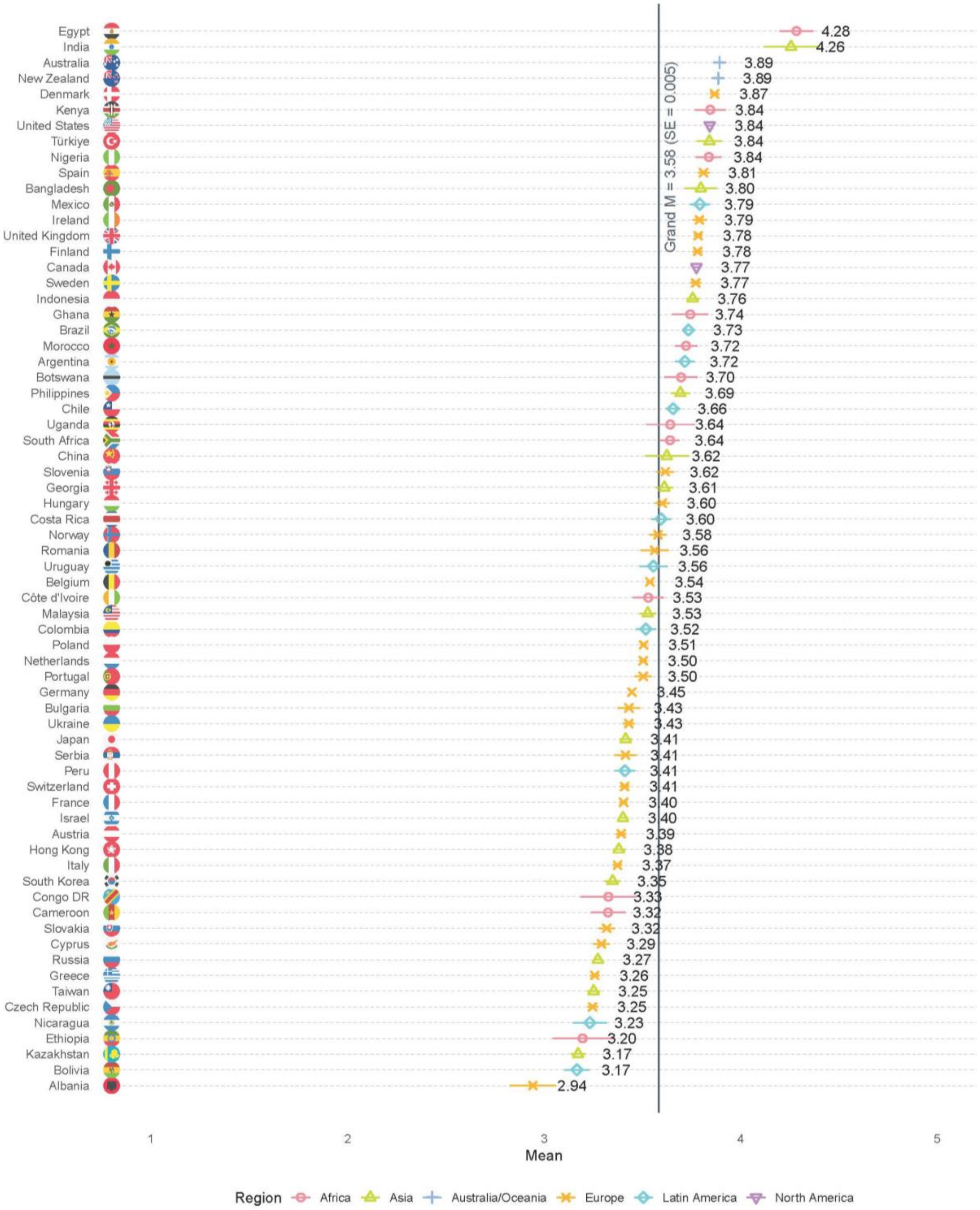

Note: Error bars means +/- standard error. Vertical line indicates the grand mean. Total *N* = 69,526. Country *N*s range between 312 and 8,011 (see table S20 for a detailed overview).

Fig. S20. Weighted means and standard errors of benevolence dimension of trust in scientists across countries

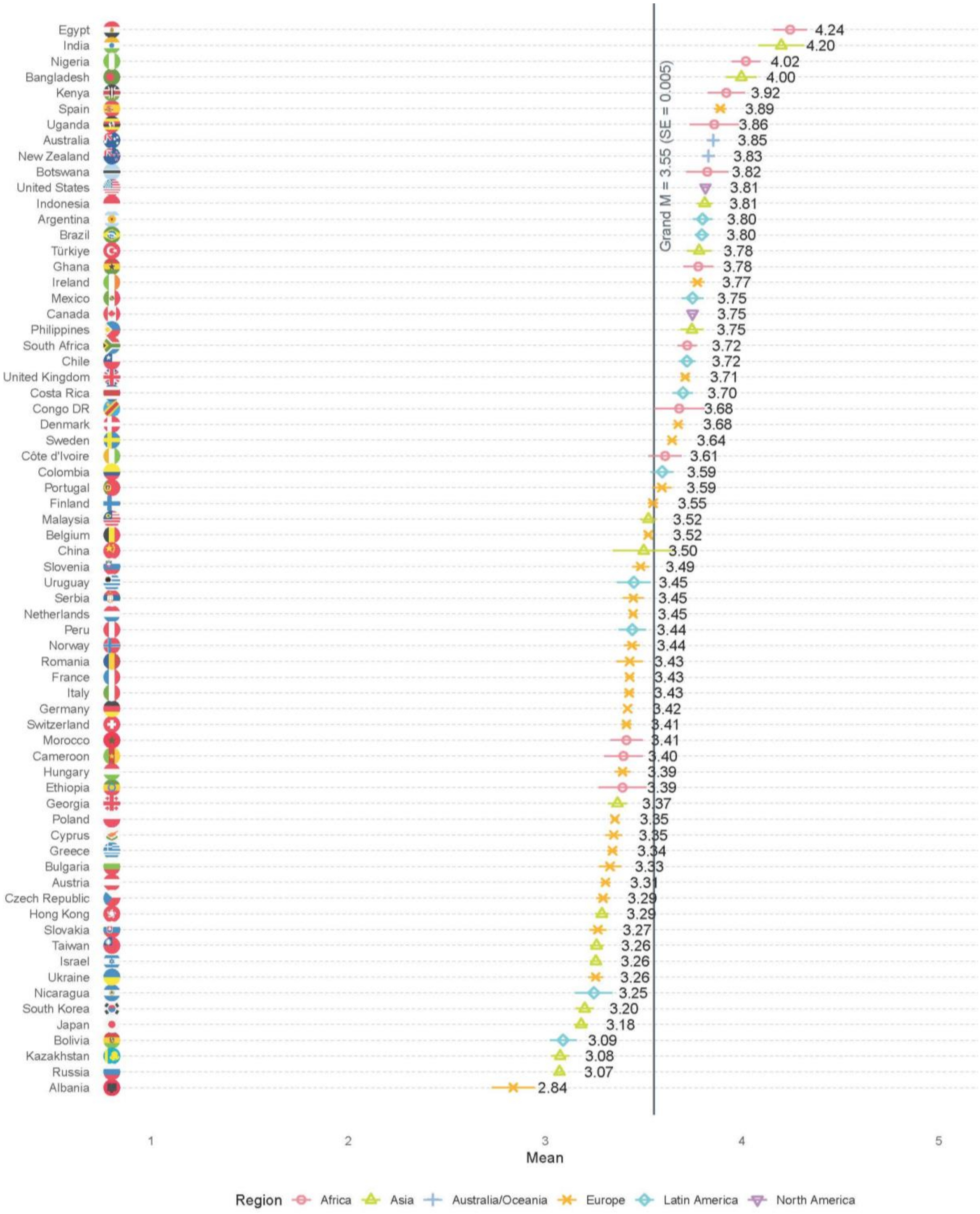

Note: Error bars means +/- standard error. Vertical line indicates the grand mean. Total N = 69,522. Country Ns range between 311 and 8,011 (see able S20 for a detailed overview).

Fig. S21. Weighted means and standard errors of openness dimension of trust in scientists across countries

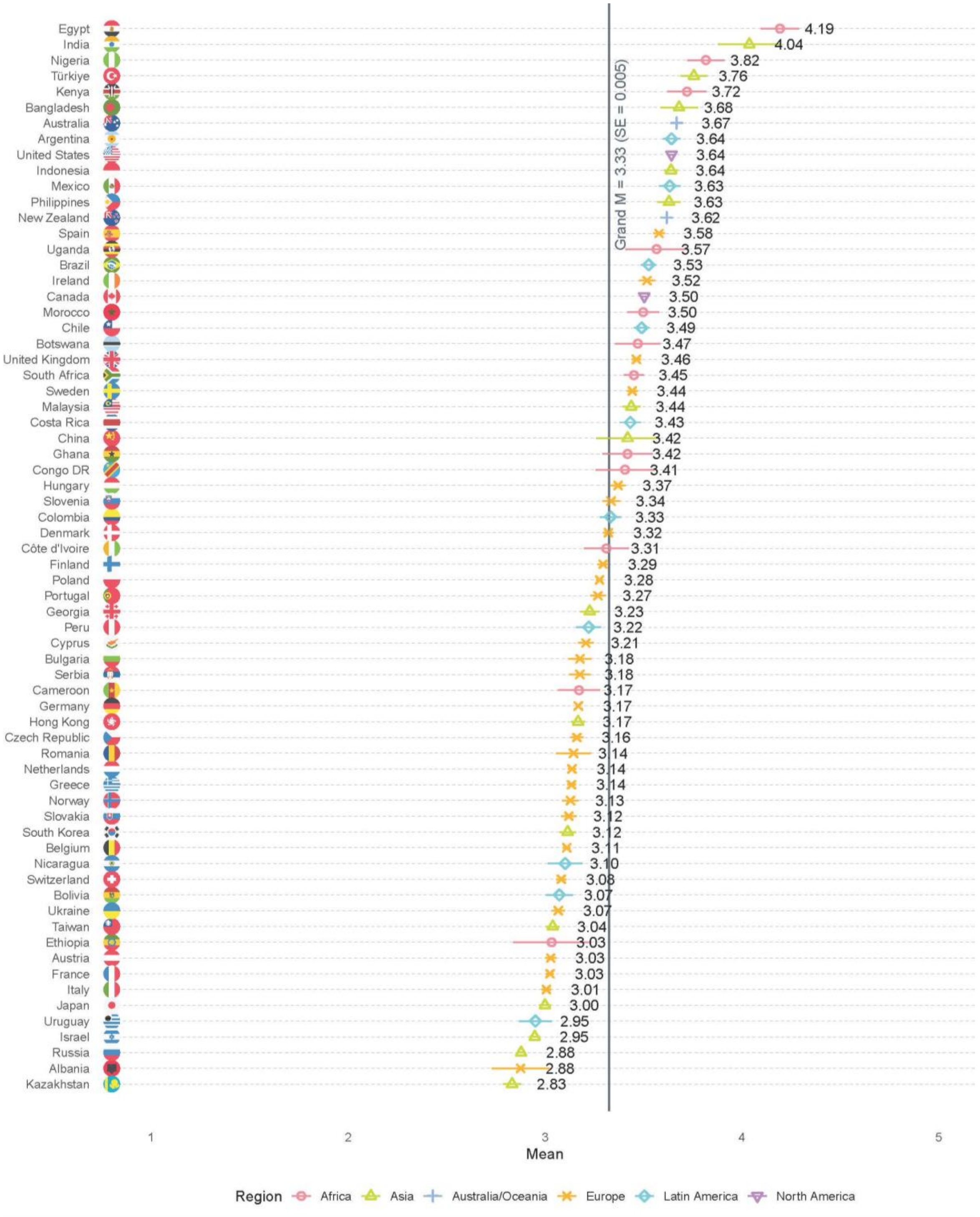

Note: Error bars means +/- standard error. Vertical line indicates the grand mean. Total *N* = 69,523. Country *N*s range between 311 and 8,011(see able S20 for a detailed overview).

Fig. S22. Weighted means and standard errors of confidence that scientists act in public interests across countries

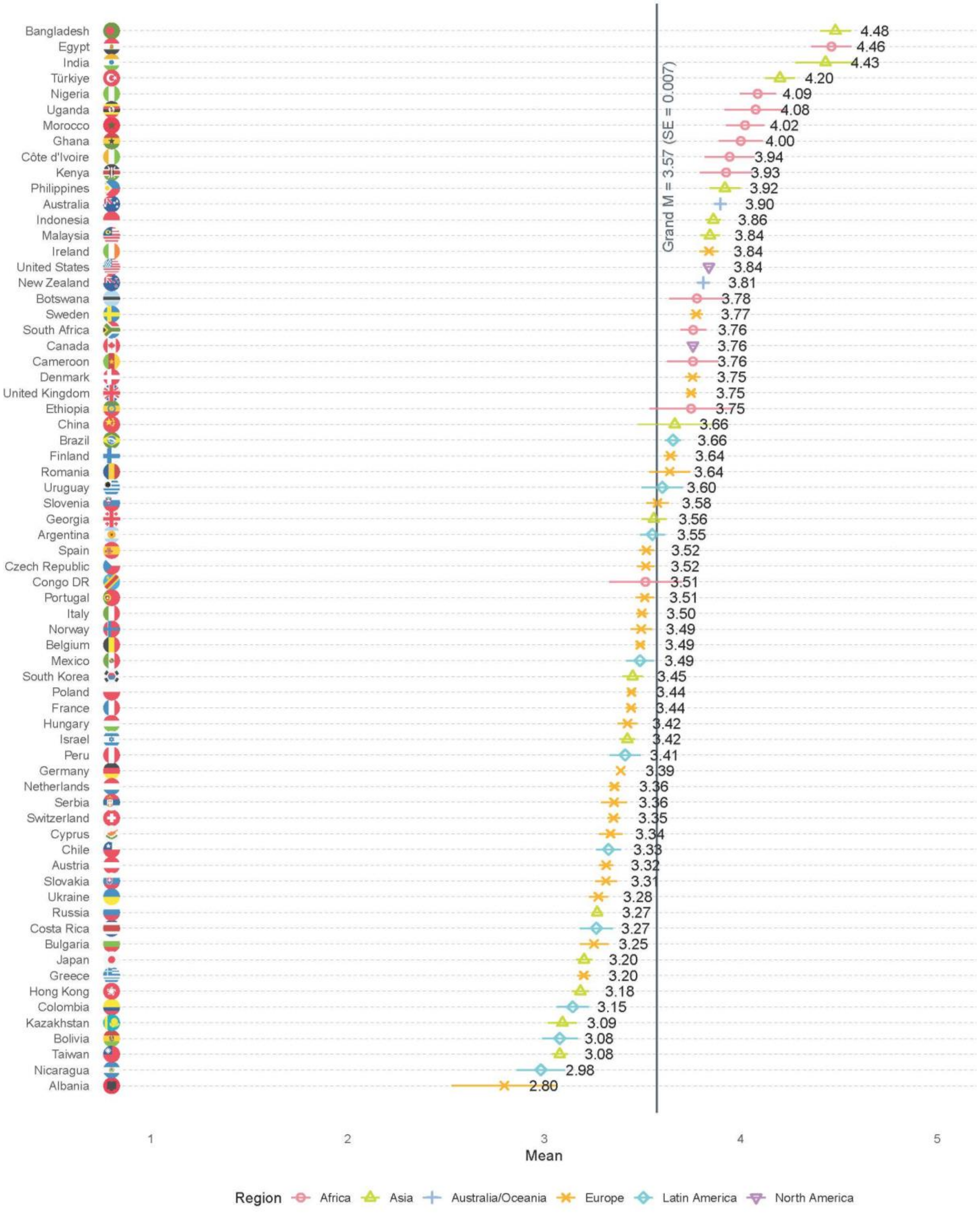

Note: Error bars means +/- standard error. Vertical line indicates the grand mean. Total N = 69,507. Country Ns range between 312 and 8,012 (see able S20 for a detailed overview).

Fig. S23. Weighted means and standard errors of trust in the scientific method across countries

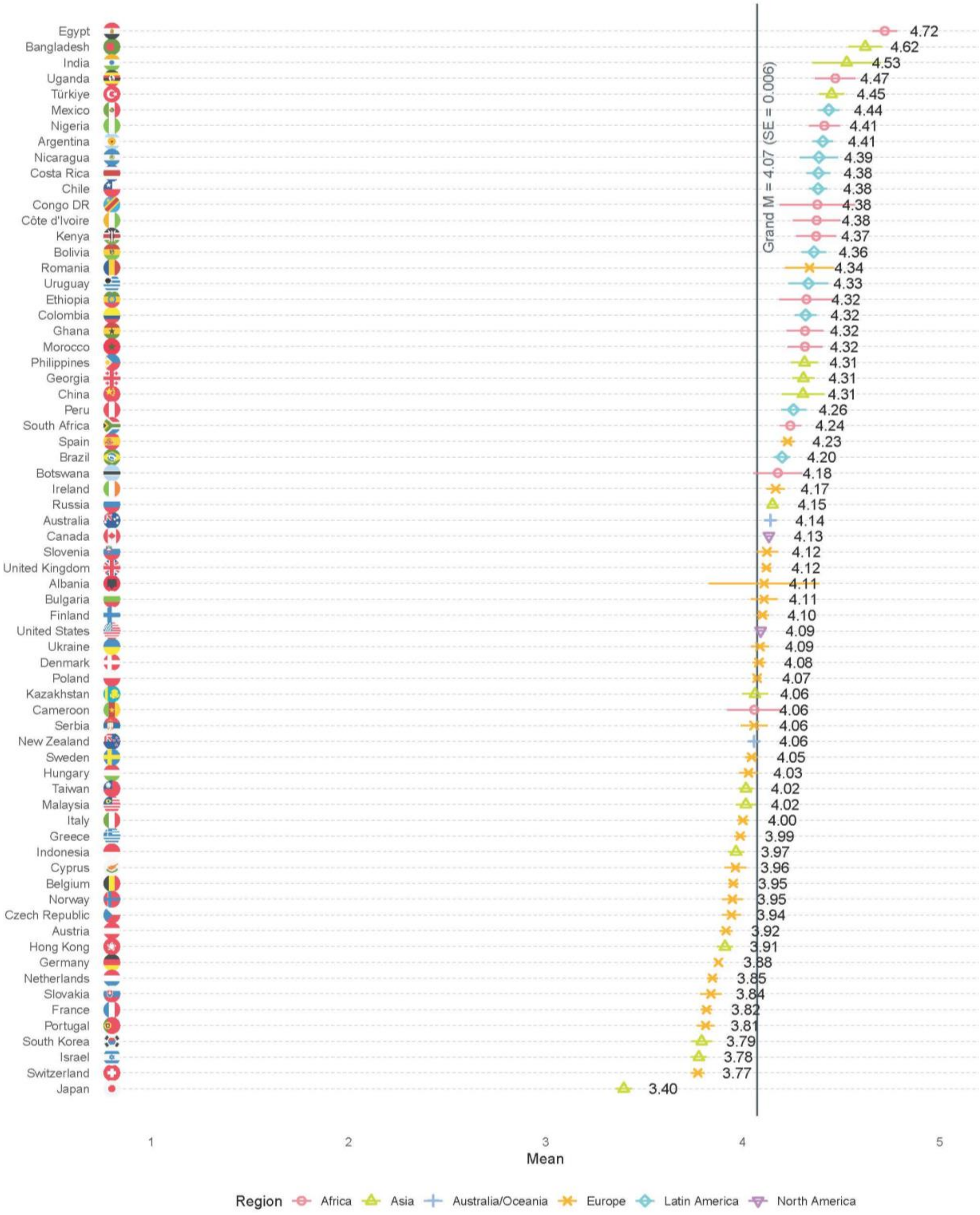

Note: Error bars means +/- standard error. Vertical line indicates the grand mean. Total N = 69,521. Country Ns range between 312 and 8,011 (see able S20 for a detailed overview).

Fig. S24. Standardised estimates of weighted blockwise multilevel regression model testing the association of confidence in scientists to act in public interest with demographic characteristics, ideological views, attitudes, and country-level indicators (random intercepts across countries)

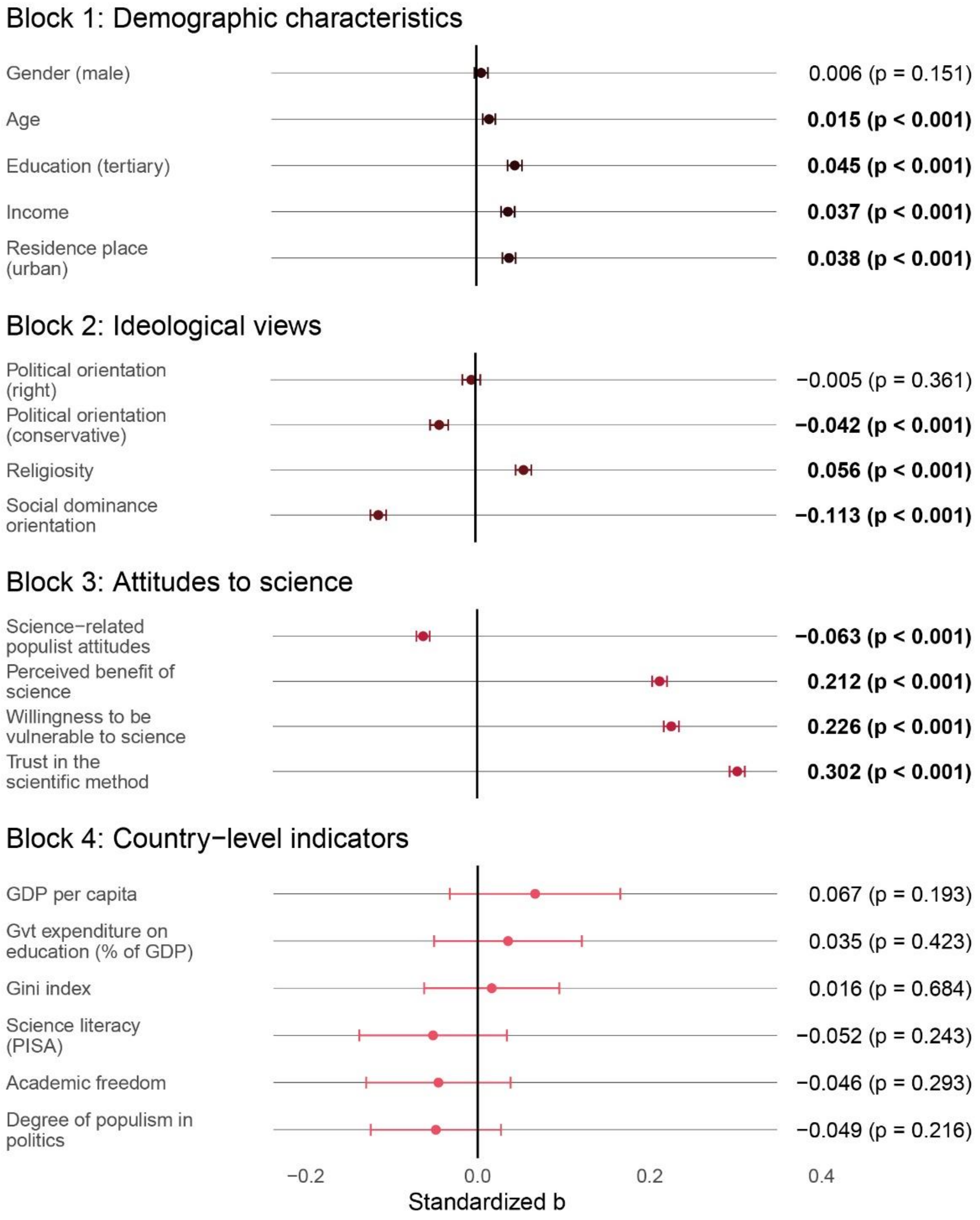

Note: Dots indicate point estimates of fixed effects, horizontal lines indicate 95% confidence intervals based on two-sided t tests. Block 1 uses data from all 68 countries, block 2 uses data from 67 countries (all except Malaysia, where social dominance orientation was not measured), block 3 uses data from 66 countries (all except Malaysia and Mexico, where willingness to be vulnerable to science was not measured), block 4 uses data from 51 countries (all except those where PISA's literacy scores were not available, see supplementary material). Full regression results are reported in table S8.

Fig. S25. Standardised estimates of weighted blockwise multilevel regression model testing the association of trust in the scientific method with demographic characteristics, ideological views, attitudes, and country-level indicators (random intercepts across countries)

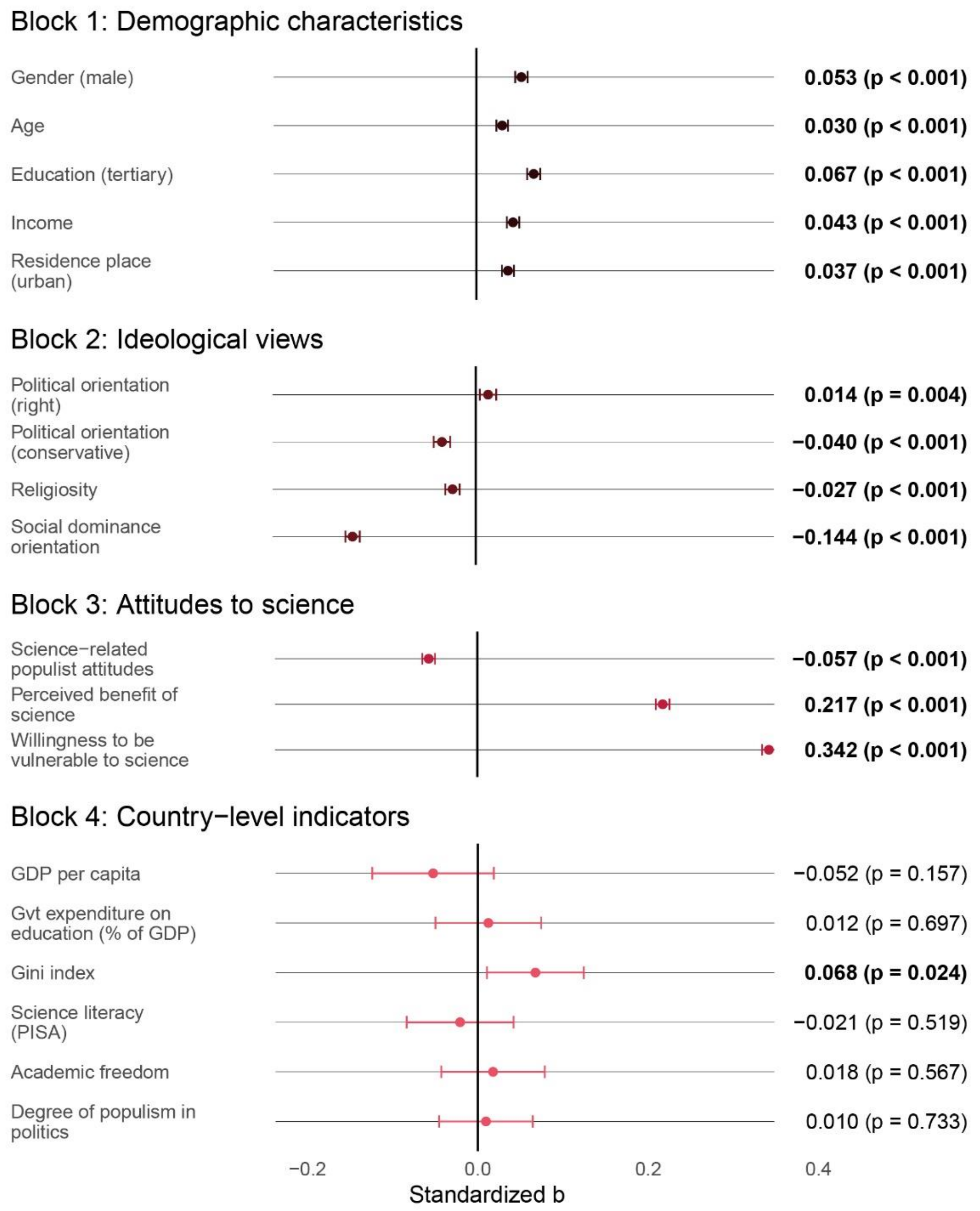

Note: Dots indicate point estimates of fixed effects, horizontal lines indicate 95% confidence intervals based on two-sided t tests. Block 1 uses data from all 68 countries, block 2 uses data from 67 countries (all except Malaysia, where social dominance orientation was not measured), block 3 uses data from 66 countries (all except Malaysia and Mexico, where willingness to be vulnerable to science was not measured), block 4 uses data from 51 countries (all except those where PISA's literacy scores were not available, see supplementary material). Full regression results are reported in table S9.

Fig. S26. Random effects of political orientation (right) on confidence in scientists across countries

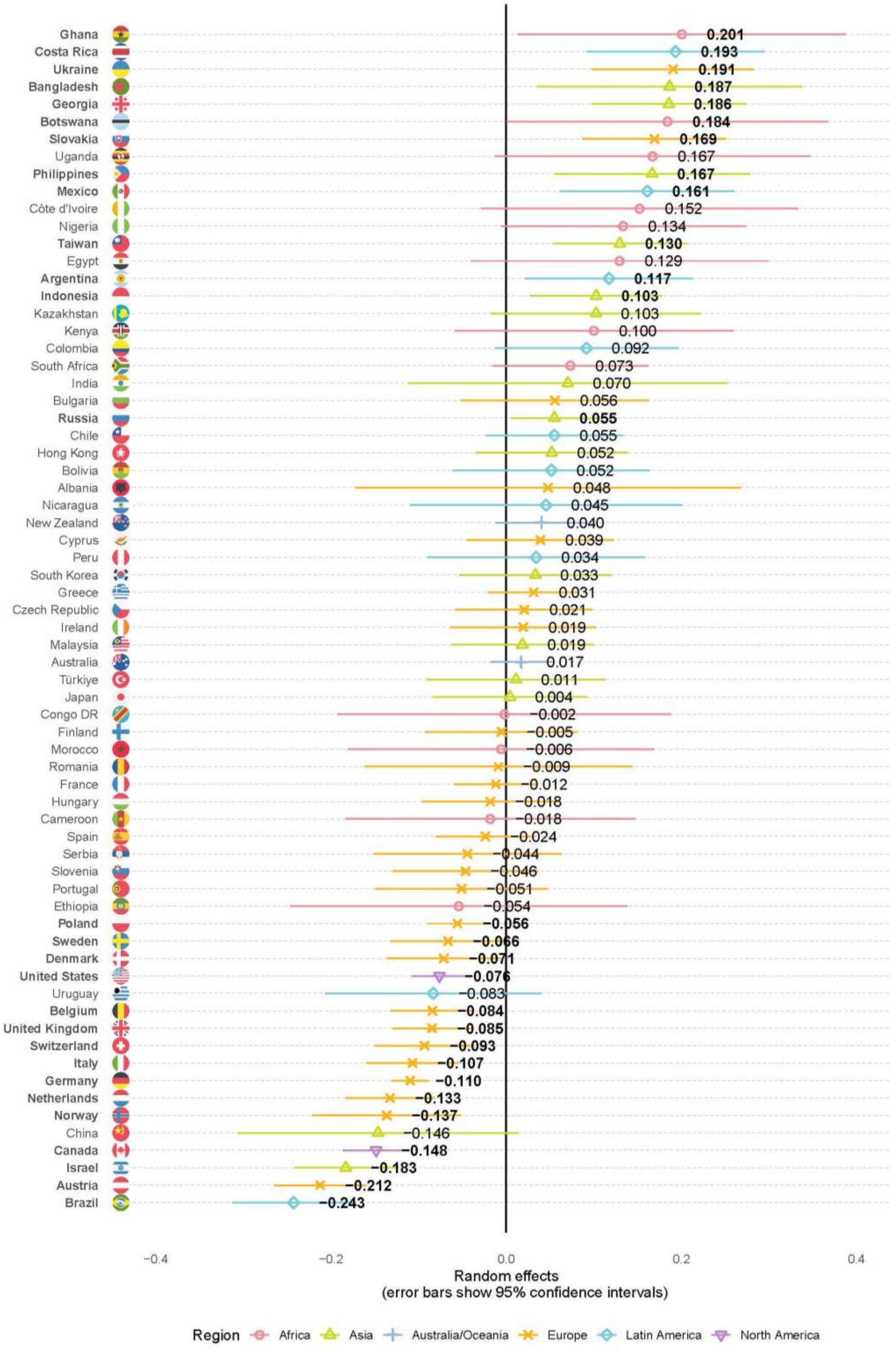

Note: Dots indicate point estimates of random effects, horizontal lines indicate 95% confidence intervals based on two-sided t tests. Effects significant at $p < .05$ are printed in bold. Total $N = 64,442$. Country *N*s range between 284 and 6,941 (see table S21 for N across countries).

Fig. S27. Random effects of political orientation (right) on trust in scientific method across countries

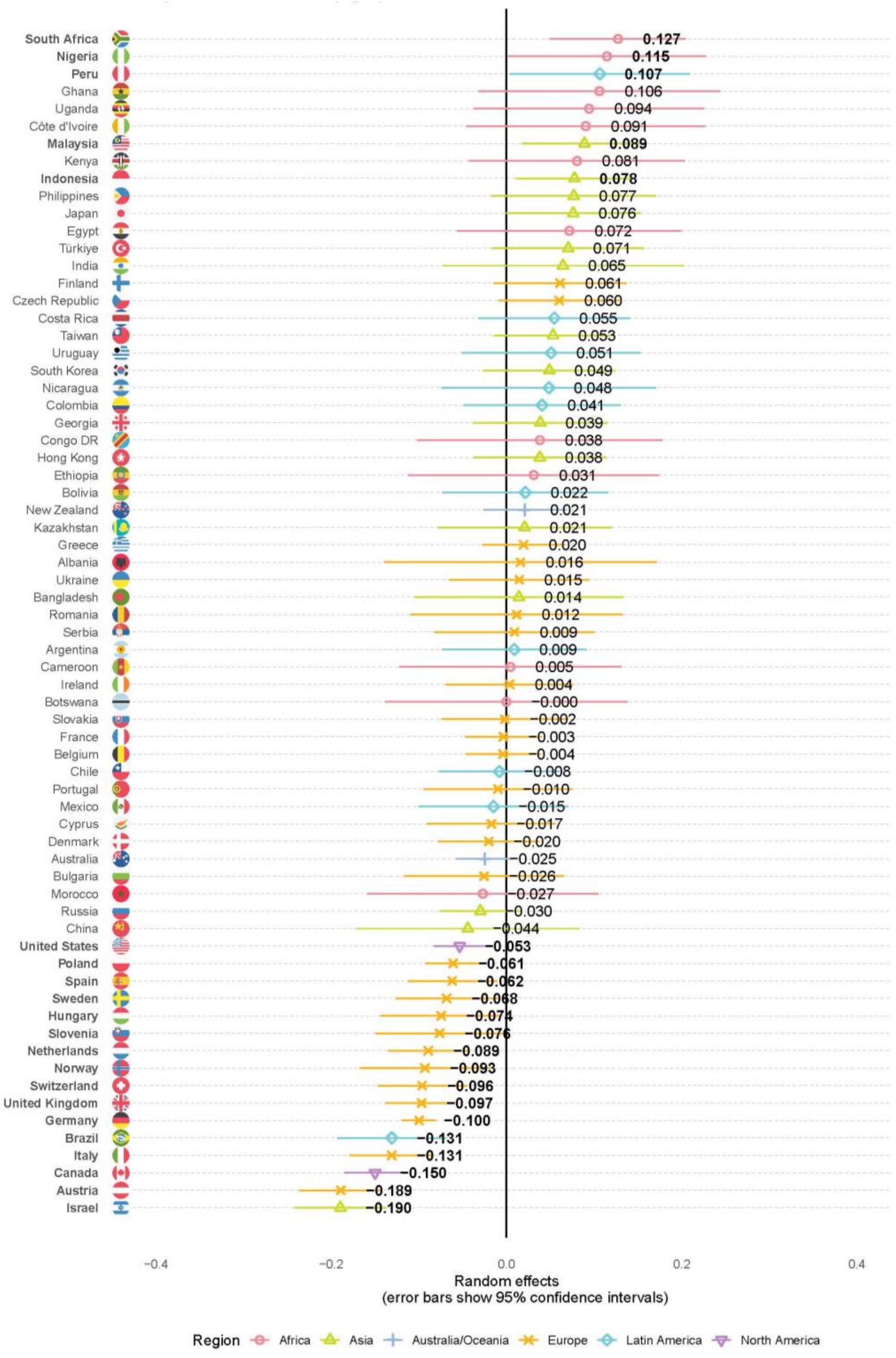

Note: Dots indicate point estimates of random effects, horizontal lines indicate 95% confidence intervals based on two-sided t tests. Effects significant at $p < .05$ are printed in bold. Total $N = 64,454$. Country *N*s range between 284 and 6,941 (see table S21 for N across countries).

Limitations

We showed all participants the following definition of science and scientists at the beginning of the survey to make sure that participants had a common definition in mind when answering the survey:

> "When we say "science", we mean the understanding we have about the world from observation and testing. When we say "scientists", we mean people who study nature, medicine, physics, economics, history, and psychology, among other things."

A similar definition had been pre-tested and used by the Wellcome Global Monitor, one of the main global studies on trust in science. It was added as results from in-depth interviews conducted by the Wellcome Global Monitor[6] suggested that including a definition would improve the reliability of cross-country comparisons. However, we are aware that introducing a very broad definition of science and scientists excludes other epistemological traditions, such as traditional knowledge or indigenous science. While our definition of science reflects the dominant and Western conception of science as an objective and apolitical way of knowing about the world, we want to acknowledge the importance of traditional knowledge and indigenous science.

The structure of our Many Labs study was such that the survey was conceptualized by the first and second author, as well as a "core team" which includes authors 3-9. The core team is composed of experts in the fields of trust in science, science in society, and behavioural science research, who were selected to achieve an expert team with diversity in terms of gender, nationality, and discipline. The fact that almost all core team members are from the Global North and that other co-authors were not involved in the development of the survey provides an important limitation of our work. However, local collaborators could make changes to the survey wherever needed, to make sure that the survey was adequate for the local context. While we are cognizant of the fact that the set-up of our Many Labs study does not fully address concerns of "parachute science", because of the limited involvement of our collaborators in the survey design, we undertook several steps to establish equitable research collaborations. This includes the opportunity to comment on a previous manuscript draft, a Qualtrics tutorial for collaborators in low-income countries to empower researchers to play a leading role in the data collection, the ability to provide feedback and network with other researchers in monthly Zoom meetings, the assistance and mentorship in writing IRB proposals and the support of early career researchers through the writing of reference letters.

There are some limitations regarding some of our socio-demographic variables, such as education and political orientation. Assessing education across countries presents a challenge given the strong differences in educational systems across the world. We therefore allowed co-authors in certain countries to add examples for primary, secondary, and tertiary education to the questionnaire that were more applicable than the ones mentioned in the main survey (e.g., high school). We also recognize the limits of asking for liberal-conservative and right-left political orientations, as these terms are uncommon or less meaningful when describing political orientations in some countries. However, using these two different measures to assess political orientation and presenting a "don't know" answer mitigated some of the concerns of only using one measure for political orientation.

A small limitation pertains to the wording of one trust item: While all items asked about perceptions of “most scientists”, the final trust in science item (TRUST_SCI_otherviews) only asked about “scientists” (i.e., How much or little attention do scientists pay to others’ views?).

Analyses of the reliability and measurement invariance of our 12-item measure for trust in scientists (see *Methods* section) indicate two limitations: First, we find that our measure has exceptionally high reliability globally, with Cronbach’s Alpha = 0.93 and Omega = 0.95. On the one hand, these high values indicate that our trust measure has high internal consistency, which is worthwhile and demonstrates its capacity to capture trust in scientists precisely. On the other hand, some have argued that exceptionally high reliability values indicate that the scale items are too redundant[96] and recommend a maximum value of Cronbach’s Alpha = 0.90[97]. Second, we find low measurement invariance of the trust measure across countries: Cronbach’s Alpha and Omega vary slightly across countries and Confirmatory Factor Analyses show that we can only assume configural invariance, yet no metric or scalar invariance (see *Methods* section). This suggests that our trust items measure slightly different perceptions in different countries and capture the latent factor “trust in scientists” better in some but less so in others. This reduces the validity of cross-country comparisons of trust in scientists (e.g., mean values, regression estimates). However, this reduction is presumably not severe – and after all, it is a common caveat of multilingual comparative survey research that is to some extent unavoidable[39].

Supplementary Methods

Please see the associated publication[38] for a detailed description of the data collection procedure, including information on languages and market research companies used in each country.

Replication materials

All analyses underlying the main article and the supplementary materials were performed with R version 4.3.3. We share data and code to replicate them analyses at the Open Science Framework: https://osf.io/wj34h

This repository contains (1) R code, precomputed statistical models, and power curve plots of the *power analyses*, (2) R code and survey data to reproduce the analysis of the *pretest survey*, and (3) R code and the data (survey data, country-level indicators, respondent IDs for duplicate checks, answers to the two open questions, population data on gender, age and education) to reproduce the analyses underlying the *main article and the supplementary materials*, as well as (4) precomputed statistical models and figures in high resolution.

Countries sampled

To qualify for co-authorship, co-authors collected at least n = 500 complete and balanced responses in each country. Exceptions were made when market research companies were not able to recruit n = 500 complete responses (e.g., in Albania, Uruguay). As shown in table S12, data was collected in k = 68 countries, thereby surveying participants in 31% of the world's countries that make up 79% of the total population. As can be seen in table S12, 42% of high-income, 32% of upper middle income, 26% of lower middle income, and 11% of low-income countries were sampled. In terms of the representativeness of surveyed countries by regions (table S13), our dataset covers more than a fourth of countries across most regions, apart from the Middle East & North Africa and Sub-Saharan Africa, where coverage is lower (14% and 21% respectively).

Table S12. Representativeness of surveyed countries by income groups.

**Income group**	***k* TISP**	***k* WB**	**% TISP**
Low income	3	28	10.71
Lower middle income	14	54	25.93
Upper middle income	17	54	31.48
High income	34	81	41.98
**Total**	**68**	**217***	**31.34**

Note. TISP = Project acronym; WB = World Bank. Based on World Bank (2021) data. *no information for Venezuela

Table S13. Representativeness of surveyed countries by regions.

Region	*k* TISP	*k* WB	% TISP
Europe & Central Asia	31	58	53.45
East Asia & Pacific	10	38	26.32
South Asia	2	8	25.00
Latin America & Caribbean	10	42	23.81
Sub-Saharan Africa	10	48	20.83
Middle East & North Africa	3	21	14.29
North America	2	3	66.67
**Total**	**67**	**218**	**31.19**

Note. TISP = Project acronym; WB = World Bank. Based on World Bank (2021) data.

Market research companies

Collaborators were instructed to work with the market research company Bilendi & Respondi, with the exception of most African countries, where collaborators collected data with MSi. A few exceptions were granted, for example, when collaborators' funding was tied to specific panel providers. Working with one market research company in each country allowed us to make sure that the same participants were not sampled twice in countries with multiple co-authors. Convenience samples were not accepted.

Preregistration

The preregistration including additional materials (e.g., R code for the power analyses, see below) can be accessed at: https://osf.io/9ksrj. There were three deviations from the preregistration:

1. We did not include the item measuring people's confidence that scientists act in the public interest as an explanatory variable in the regression models explaining trust in scientists, albeit we had stated that in the preregistration. This was because this item had a very high correlation with the trust in scientists index (weighted Pearson's $r(69{,}502) = 0.707$ $p < .001$, $t = 301.71$, 95% CI 0.705 to 0.709), which would have led to a substantial inflation of explanatory power, with Conditional R^2 being well above 0.70 (see *Analytical Procedures* for more details).
2. The preregistration did not mention social dominance orientation as a covariate in the regression model testing predictors of normative perceptions of the role of science in society and politics (RQ4). This omission was a mistake. Replicating the procedure of the RQ1, RQ2, RQ3, and RQ5 analyses, we also included social dominance orientation as covariate in the RQ4 analyses.
3. When computing the post-stratification weights via raking (see below), we collapsed single neighbouring age and education strata for some countries: This was because some age and education strata were empty or sparsely populated in several countries, because collaborators had to relax age quotas or oversampled individuals with tertiary education

to reach their target sample size. However, raking is not possible with empty strata and results in extreme weights when applied to data with sparsely populated strata. Therefore, we collapsed empty or sparsely populated age and education strata with adjacent strata in cases where a stratum contained less than 5% of respondents within a country.

We tested all our research questions and hypotheses. However, we do not present results of all these tests in the main article and the supplementary materials, because we did not have enough space or believed they would not fit the focus and narrative of the main article. The research questions and hypotheses whose test results we do not present in the main article are listed in table S14. The preregistered analyses for all of these research questions and hypotheses can be obtained via the code and information contained in the replication materials.

Table S14. Preregistered research questions and hypotheses whose test results are not presented in the main article.

RQ/H	Wording
RQ1b	How much do publics across different countries endorse science-related populist attitudes?
RQ2b	Which individual-level variables predict science-related populist attitudes globally?
H2b-1	Science-related populist attitudes are associated with the following variables: (1) gender, (2) age, (3) educational attainment, (4) income, (5) urban/rural region of residence, (6) political orientation, (7) conservatism, (8) social dominance orientation, and (9) religiosity.
H2b-2	Science-related populist attitudes are associated with the following variables: (1) trust in scientists, (2) perceived benefit of science, (3) willingness to be vulnerable to scientists, (4) trust in the scientific method, and (5) confidence in scientists to act in the public's interest.
RQ3b	How does the effect of individual-level and country-level predictors on science-related populist attitudes differ between countries?
H3b-1	The effects of the following variables (individual level) on science-related populist attitudes vary across countries: (1) gender, (2) age, (3) educational attainment, (4) income, (5) urban/rural region of residence, (6) political orientation, (7) conservatism, (8) social dominance orientation, and (9) religiosity.
H3b-2	The effects of the following variables (individual level) on science-related populist attitudes vary across countries: (1) trust in scientists, (2) perceived benefit of science, (3) willingness to be vulnerable to scientists, (4) trust in the scientific method, and (5) confidence in scientists to act in the public's interest.
H3b-3	The effects of the following indicators (country level) on science-related populist attitudes vary across countries: (1) GDP per capita, (2) Government expenditure on education (% of GDP), (3) GINI Index, (4) PISA Scientific Literacy Score, (5) Academic Freedom Index, and (6) prevalence of populist rhetoric in political discourse.
RQ4b	How do science-related populist attitudes relate to normative perceptions of the role of science in society and politics?
H4b-1	Science-related populist attitudes are negatively associated with demanding an active role of scientists in society and politics.
H4b-2	The association of science-related populist attitudes and normative perceptions varies between countries.

H5-2	Science-related populist attitudes are positively correlated with a higher difference between perceptions and expectations.
H5-3	The correlations hypothesized in H5-1[a] and H5-2 interact with the following individual-level variables: (1) political orientation, (2) conservatism, (3) religiosity, (4) perceived benefit of science, (5) willingness to be vulnerable to scientists, (6) trust in the scientific method, and (7) confidence in scientists to act in the public's interest.
H5-4	The correlations hypothesized in H5-1[a] and H5-2 interact with the following country-level variables: (1) GDP per capita, (2) Government expenditure on education (% of GDP), (3) GINI Index, (4) PISA Scientific Literacy Score, (5) Academic Freedom Index, and (6) prevalence of populist rhetoric in political discourse.
RQ6a	Which world regions do people believe benefit most from science?
RQ6b	Which world regions do people believe benefit least from science?
RQ6c	How do the beliefs addressed by RQ6a and RQ6b differ depending on where respondents are from?

Note. [a] H5-1 was as follows: Trust in scientists is positively correlated with a smaller difference between perceptions and expectations.

Power analysis

First, we simulated datasets with $n \times k$ observations, with n = target sample size per country and k = target number of countries. We chose n we chose a value 500 and for k we chose the values 3 and 15. Note that the simulated data are considerably smaller than those later collected in the main study, because power analyses for generalized linear mixed models require considerable computation power. Using sample sizes matching the scope of the TISP project (e.g., 65,000 with n = 1,000 in k = 65 countries, which was our estimate at that time) would thus have led to an extremely long computation duration. The simulated datasets contained the ten variables: the four dimension scores of our 12-item measure for trust in scientists (competence, integrity, benevolence, openness) and the aggregate score for trust in scientists (mean of the four dimension scores) as well as the four dimension scores of our 8-item measure for science-related populist attitudes (conceptions of the ordinary people, conceptions of the academic elite, demands for decision-making sovereignty, demands for truth-speaking sovereignty) and the aggregate score for science-related populist attitudes (smallest value of the four dimension scores, i.e. the "Goertz score", see[98]; see *Indices* section). Please note that we also ran power analyses for science-related populist attitudes (albeit the main article focuses on trust in scientists), because the preregistration and early versions of the manuscript centered on both trust in scientists and science-related populism.

When simulating the datasets, we derived plausible average values for the dimension scores from existing studies which had used our measures for trust in scientists[48] and science-related populist attitudes[99].

Second, we simulated linear multilevel regression models that contained random intercepts across the k countries and predicted outcome y with the aggregate scores of (1) trust in scientists or (2) science-related populist attitudes. When simulating the regression models, we specified different plausible effect sizes of trust in scientists (b = 0.20 and b = 0.10) and science-related populist attitudes (b = 0.10 and b = 0.05), also derived from previous research (e.g., *44*, *47*, *53*). Note that we expected smaller effect sizes for science-related populist attitudes as we rely on the

“Goertz” aggregation approach, which has been found to lead to more conservative effect estimates[98,100]. For further details on the simulations and parameter specifications (global intercept, standard deviations of random intercepts, residual variances), see the R code we share at: https://osf.io/wj34h.

Third, we ran four power analyses (1,000 model simulations, alpha level = 0.05). They gave the following results:
(1) With an overall sample size of 1,500 (n = 500 in k = 3 countries), we reach a power of 87.00% (95% CI: 84.76, 89.02) to detect a fixed effect of b = 0.20 for trust in scientists on an outcome variable y (see power curve in fig. S28).

Fig. S28. Power curve of simulation-based power analysis for effects of trust in scientists (*b* = 0.20, *k* = 3 countries).

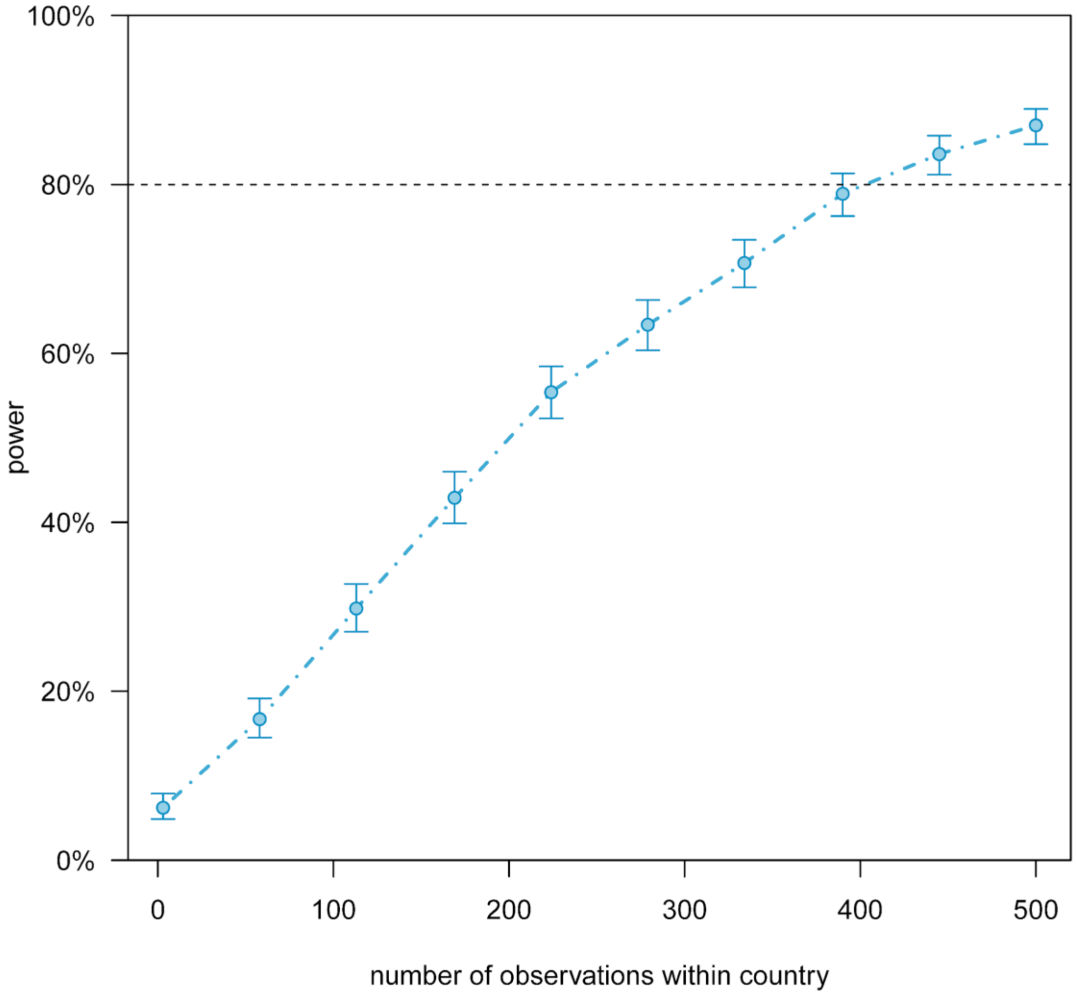

Note: Dots indicate point estimates of statistical power for different numbers of observations within countries. Vertical lines indicate 95% confidence intervals.

(2) With an overall sample size of 1,500 (n = 500 in k = 3 countries), we reach a power of 86.50% (95% CI: 84.22, 88.56) to detect a fixed effect of b = 0.10 for science-related populist attitudes on an outcome variable y (see power curve in fig. S29).

Fig. S29. Power curve of simulation-based power analysis for effects of science-related populist attitudes (*b* = 0.10, *k* = 3 countries).

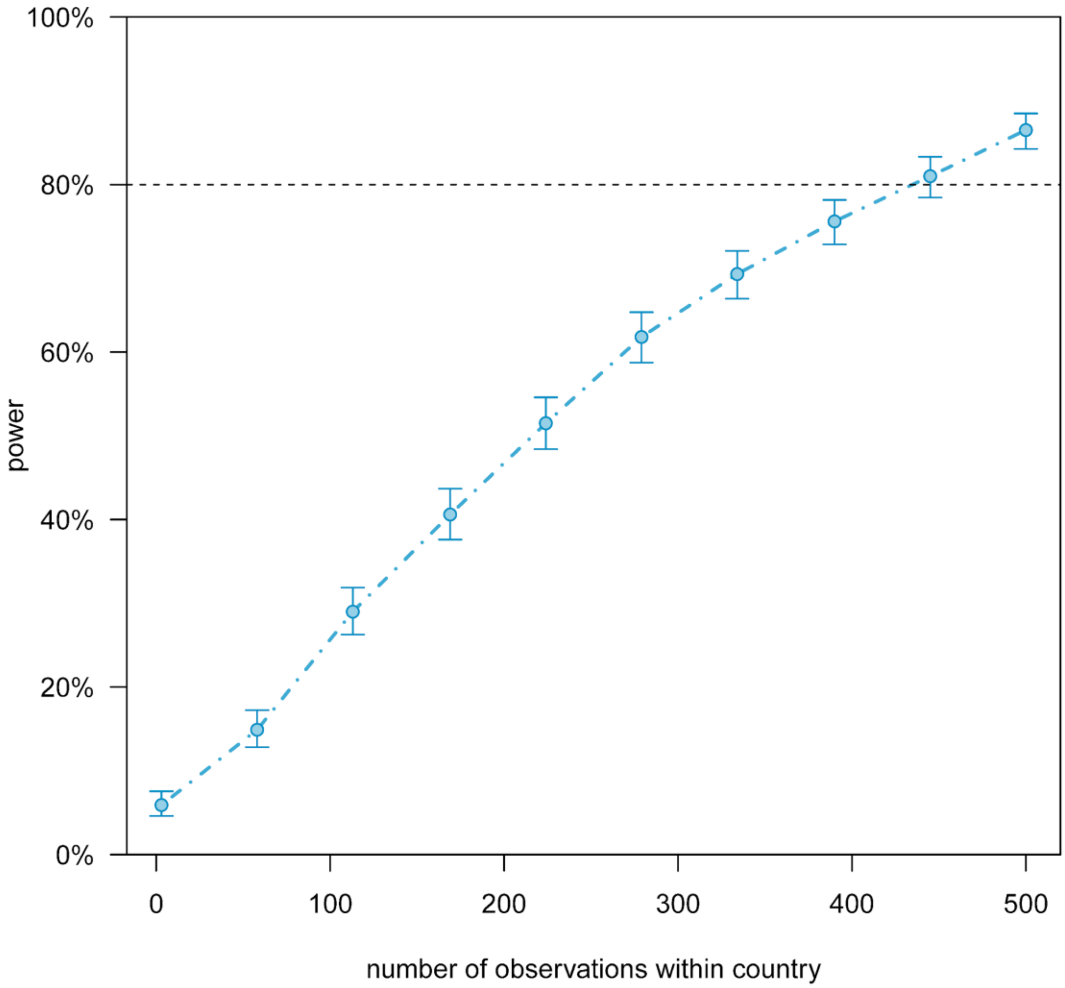

Note: Dots indicate point estimates of statistical power for different numbers of observations within countries. Vertical lines indicate 95% confidence intervals.

(3) With an overall sample size of 7,500 (n = 500 in k = 15 countries), we reach a power of 95.30% (95% CI: 93.80, 96.53) to detect a fixed effect of b = 0.10 for *trust in scientists* on an outcome variable y (see power curve in fig. S230).

Fig. S30. Power curve of simulation-based power analysis for effects of trust in scientists (b = 0.10, k = 15 countries).

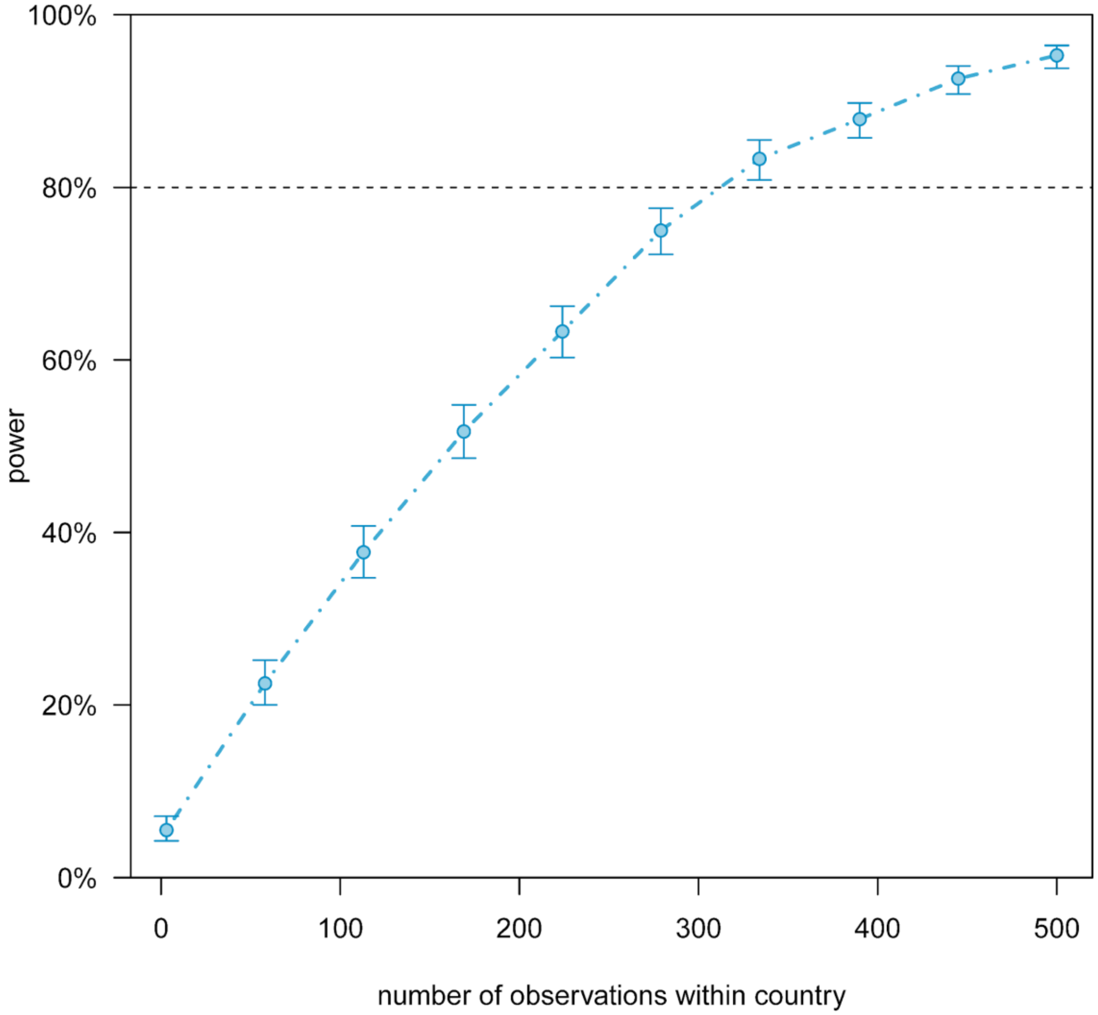

Note: Dots indicate point estimates of statistical power for different numbers of observations within countries. Vertical lines indicate 95% confidence intervals.

(4) With an overall sample size of 7,500 (n = 500 in k = 15 countries), we reach a power of 90.00% (95% CI: 87.97, 91.79) to detect a fixed effect of b = 0.05 for science-related populist attitudes on a given outcome variable (see power curve in fig. S31).

Fig. S31. Power curve of simulation-based power analysis for effects of science-related populist attitudes (*b* = 0.05, *k* = 15 countries).

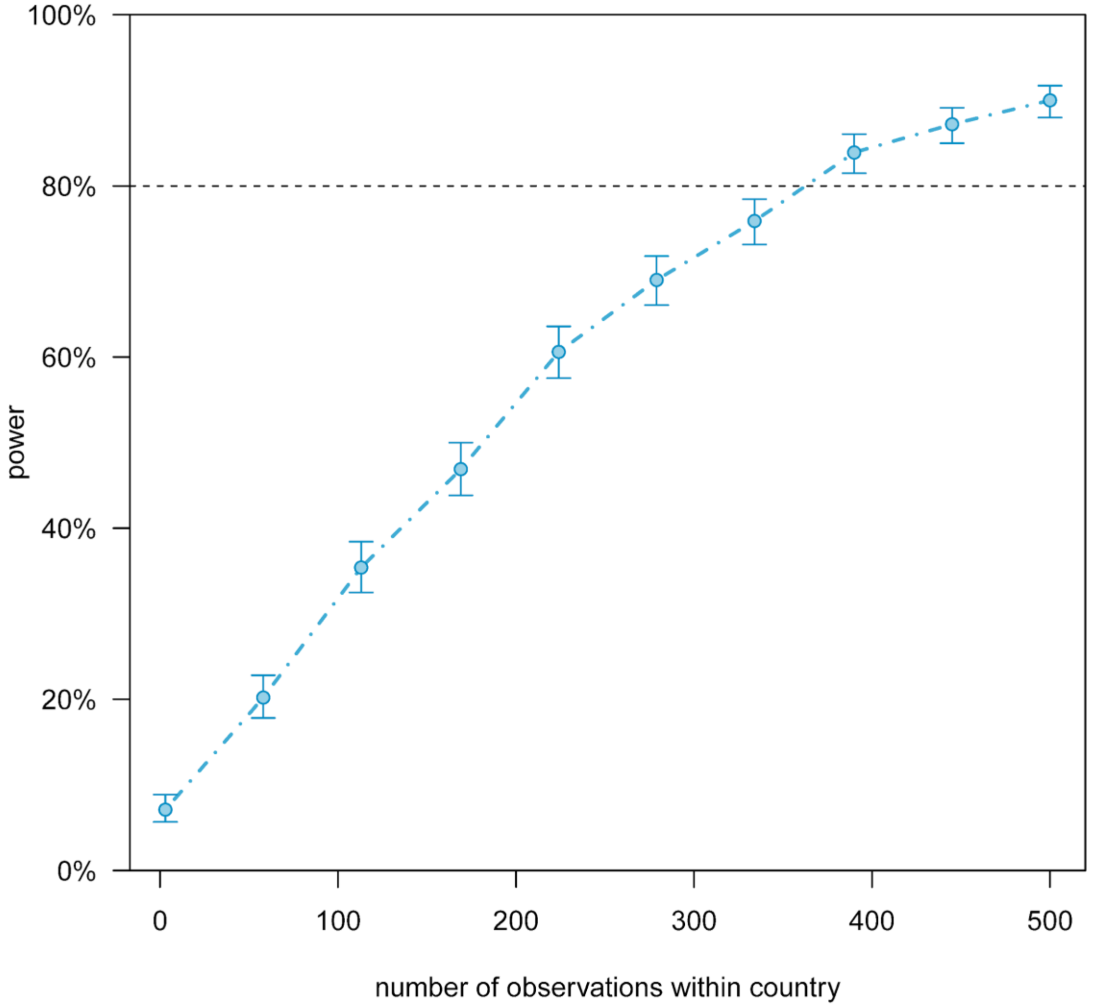

Note: Dots indicate point estimates of statistical power for different numbers of observations within countries. Vertical lines indicate 95% confidence intervals.

Based on these analyses we determined a minimum target sample size of 7,500, with n = 500 in k = 15 countries to detect fixed effects of trust in scientists and science-related populists as small as b = 0.10 and b = 0.05, respectively. Our final sample of 71,922 individuals with k = 68 countries is thus by far big enough to detect even smaller effects of trust in scientists and science-related populist attitudes.

The dataset

The collaborators submitted the complete data sets to the project team, i.e., including respondents who did not complete the survey. All these 88 datasets (68 countries) were then merged to a single dataset. Please see the associated publication[38] for a detailed description of how the final data set was prepared[38.] This publication provides a detailed description of data cleaning and pre-processing procedures (exclusion of duplicate respondents, including the computation of post-stratification weights used in the analyses (see section *Analytical Procedures*). It also contains weighted and unweighted descriptives of the global sample and all country samples, tests the performance of the attention checks, and presents comprehensive tests of the psychometric properties of the trust in scientists scale and the SDO scale – i.e., tests of the internal consistency of the scales (Cronbach's Alpha and Omega), exploratory and confirmatory factor analyses investigating their dimensional structures, analyses of measurement invariance across countries, and convergent validity tests.

Country indicators

We added six country-level indicators to the TISP dataset[38], following the procedure described in the preregistration.

1) *Gross domestic product per capita*: To measure the wealth of each country, we drew on the gross domestic product (GDP) per capita in US dollars. We used 2021 figures provided by the World Bank and the OECD, which are the most recent data[101]. If there were no 2021 data available, we used the most recent value from 2016-2020. There was one exception: We relied on official data of the local government for Taiwan, as the World Bank and OECD do not provide GDP data for it[102].

2) *Government expenditure on education in % of GDP*: To measure how much priority each country attributes to school and academic education, we used data on expenditures of local, regional and central governments on education (current, capital, and transfers) as a percentage of GDP. We relied on 2021 World Bank values that were collected by the UNESCO Institute for Statistics[103], which are the most recent data. If there were no 2021 data available, we used the most recent value from 2016-2020. There were two exceptions: We used data from 2013 for Nigeria, because more recent data were not available, and relied on official data of the local government for Taiwan, as the World Bank does not provide data on education expenditures it[104].

3) *Gini Index*: To measure the extent to which the distribution of income among individuals or households within each country deviates from a perfectly equal distribution, we used the Gini Index. A Gini Index of 0 represents perfect equality, while an index of 100 implies perfect inequality. We used 2021 scores provided by the World Bank, which are the most recent data[105]. If there were no 2021 data available, we used the most recent value from 2016-2020. There were some exceptions: We used earlier scores for the Democratic Republic of the Congo (2012), Cameroon (2014), Botswana (2015), Ethiopia (2015), Kenya (2015), Japan (2013), Malaysia (2015), Morocco (2013), Nicaragua (2014), and South Africa (2014), because more recent data were not available. Moreover, we relied on World Economics, a UK-based data service owned by Information Sciences Ltd., who estimated Gini scores for countries not covered by the World Bank dataset,

using additional data sources[106]. These countries/territories were New Zealand, Hong Kong, and Taiwan.

4) *PISA science performance score*: To measure a population's average literacy "in the use of scientific knowledge to identify questions, acquire new knowledge, explain scientific phenomena, and draw evidence-based conclusions about science-related issues" within each country, we relied on the Science Performance indicator of the Programme for International Student Assessment (PISA) for each country. We used scores provided by the OECD, which are based on data by the most current PISA study from 2018[107]. Since these data only include scores for OECD countries, we also included data from partner studies in non-OECD countries or jurisdictions[108]. If there were no 2018 data available, we used the most recent scores from 2006-2015, which was when previous PISA studies were conducted. Please note that there are still several countries which participated neither in the main PISA study nor in the partner studies, so we could not add science performance scores to the survey data for them. These countries are: Bangladesh, Bolivia, Botswana, Cameroon, the Democratic Republic of the Congo, Côte d'Ivoire, Egypt, Ethiopia, Ghana, India, Kenya, Nicaragua, Nigeria, South Africa, and Uganda.

5) *Academic freedom*: To measure academic freedom across countries, we drew on the Academic Freedom Index, which is a score provided by the Varieties of Democracy (V-Dem) Project and the University of Erlangen–Nuremberg[109]. It is based on a survey of 2,050 country experts who assessed "the freedom to research and teach, the freedom of academic exchange and dissemination, the institutional autonomy of higher education institutions, and campus integrity"[110]. The score ranges between 0 = no freedom and 1 = perfect freedom. We used 2022 data but relied on the most recent scores from 2017-2021 if no 2022 data were available. Academic Freedom Index values of the countries included in the TISP project ranged between 0.977 for the Czech Republic and 0.070 for China (M = 0.720, SD = 0.266).

6) *Prevalence of populist rhetoric in politics*: To measure how much political discourse in each country is determined by populist actors and their arguments, we used a score that is based on the Global Party Survey[111] and was introduced by[20]. It is a continuous score that is "composed from two items measured in the Global Party Survey 2019, which asked 1,861 experts to assess the agendas and rhetoric of 1,043 political parties in 163 countries[111]. To obtain this index, we proceeded as follows: First, we selected two items that asked experts how strongly each party in a given country favours populist vs. pluralist rhetoric (11-point Likert scales, 0 = strongly favours pluralist rhetoric – 10 = strongly favours populist rhetoric) and how important populist rhetoric is for each party in that country (11-point Likert scales, 0 = no importance – 10 = great importance). Second, we computed mean values of these two items across all parties within each country. Third, we averaged these values, which resulted in a single aggregate measure for the prevalence of populist rhetoric within national political discourses" [20] (p. 3). Higher values thus indicate higher prevalence, with a range between 2.759 for Canada and 8.375 for Kazakhstan (M = 5.588, SD = 1.143). Note that there was no Global Party Survey data available for China and Hong Kong, so we used proxy estimates derived from the relevant literature. For China, recent scholarly work suggests that the Chinese political system and its leader Xi Jinping feature several aspects of populism, authoritarianism, and anti-pluralism, so we assigned a relatively high score of 7.00[112–114].

> For Hong Kong, researchers suggest that political discourse has some populist elements, which is due to populist rhetoric of politicians and parties, but mostly due to grassroots movements and localist groups, so we assigned a medium score of 6.00[115–118].

These country indicators can be conceived as proxies for cultural and political similarities between countries. Including them in (most of) our models (see section *Analytical Procedures)* thus enabled us to not only test our hypotheses – but also to account for recent critiques that cross-national studies often do not control for similarities, proximity, and connectedness between the clusters in their samples (i.e., countries), such as common cultural values and political climates, which may violate assumptions of non-independence and lead to higher false positive rates[119].

Analytical Procedures

Below we describe the analyses underlying the results presented in the main article and the Supplementary Materials. They are entirely reproducible with the materials shared at: https://osf.io/wj34h

Indices

Trust in scientists was measured with the unweighted mean of all twelve items capturing four conceptual dimensions of trust in scientists, i.e., competence, integrity, benevolence, and openness (weighted $M = 3.62$, $SD = 0.70$). Higher values indicate higher trust (range: 1 – 5). Scale reliability was very high (Cronbach's Alpha = 0.93; Omega = 0.95; see Mede et al.[46] for a detailed analysis of the psychometric properties and measurement performance of the scale).

Science-related populist attitudes were measured with a score that was based on mean values of the four dimensions of the SciPop scale, i.e., conceptions of the ordinary people, conceptions of the academic elite, demands for decision-making sovereignty, demands for truth-speaking sovereignty (weighted $M = 2.32$, $SD = 0.91$). Higher values indicate stronger science-related populist attitudes (range: 1 – 5). In particular, we followed the "Goertz approach"[98]. It suggests using the smallest dimension mean as an indicator for one's level of science-related populist attitudes. This procedure accounts for the conceptual premise that all four components have to be concurrently present to diagnose science-related populist attitudes, whereas absence of one or more components would disqualify someone to be classified as a proponent of science-related populism[38]. Scale reliability was high (Cronbach's Alpha = 0.79, Omega = 0.87; see Mede et al.[46] for a detailed analysis of the psychometric properties and measurement performance of the scale).

Social dominance orientation (SDO) was measured with the unweighted mean of the respective three items (weighted $M = 3.62$, $SD = 1.76$, range: 1 – 10). Scale reliability was mediocre (Cronbach's Alpha = 0.57, Omega = 0.59; see Mede et al.[46] for a detailed analysis of the psychometric properties and measurement performance of the scale).

Willingness to be vulnerable to scientists was measured with the unweighted mean of the respective three items (weighted $M = 3.85$, $SD = 0.80$). Scale reliability was good (Cronbach's

Alpha = 0.75, Omega = 0.75). Higher values indicate higher willingness to be vulnerable (range: 1 – 5).

Normative perceptions of the role of science in society and politics were measured with the unweighted mean of the respective five items (weighted M = 3.64, SD = 0.87). Scale reliability was good (Cronbach's Alpha = 0.74, Omega = 0.75). Higher values indicate stronger demands for a more active role of scientists in society and politics (range: 1 – 5).

The discrepancy of desired research priorities and perceptions of actual research efforts and was measured with a score that captures if people's desires that scientists should prioritize certain issues match or deviate from people's perceptions that science actually tackles these issues. Higher values indicate that science's actual efforts to tackle those issues are more likely to exceed people's desires that scientists should prioritize them. Accordingly, lower values indicate that science's actual efforts to tackle those issues are more likely to stay behind people's desires that scientists should prioritize them (range: -4 – 4). The score was composed as follows: First, we subtracted response values for desired priorities from perceptions of actual efforts for each of the four items (improving public health, solving energy problems, reducing poverty, developing defence and military technology). This gave us four discrepancy scores, one for each issue. Second, we computed the mean of these four scores (weighted M = -0.41, SD = 0.80). Scale reliability was acceptable (Cronbach's Alpha = 0.45, Omega = 0.67).

Scaling

We scaled all continuous and binary level-1 variables (i.e., variables measured at the level of respondents) within countries and all level-2 variables (i.e., indicators measured at the level of countries) within the full sample: For each level-1 variable, we subtracted the unweighted within-country mean from the response values and divided them by the unweighted within-country standard deviation. For each level-2 variable, we subtracted the unweighted grand mean from the indicator values and divided them by the unweighted global standard deviation. This was to prepare the variables for multilevel regression modelling; it allows valid comparisons of regression estimates, faster model convergence, and compensation of potential multicollinearity issues[120].
We used within-cluster transformation instead of grand mean/SD transformation for level-1 variables because this enabled us to distinguish country-level effects from individual-level effects[121]. Note that we scaled the variables instead of only centering them because some variables were on very different scales (e.g., age, income, and political orientation, or the Gini Index and the Academic Freedom Index). Our decision to scale both continuous and binary variables was because only scaling continuous independent variables in regression models can lead to conflated estimates for categorical independent variables[122].

Analysis of average levels of trust in scientists

To assess average levels of trust in scientists, we computed weighted mean values and SEs of the trust index (average of responses to the 12 items measuring trust in scientists, see *Indices* section). This analysis relied on a survey design object that was created with the R package

survey[84] (v4.4-2) and contained post-stratification weights at country level and sample size weights for each country to account for different selection probabilities across genders (male / female), age groups (18-29 / 30-39 / 40-49 / 50-59 / 60+ years), education levels (none or primary / secondary / tertiary education), and countries (due to different sample sizes). See table S20 for an overview of valid N across countries. See Mede et al.[46] for more information.

Analysis of factors explaining trust in scientists

The independent variables were included in four blocks. See table S21 for an overview of valid N across countries.

- Block 1 contained all variables measuring demographic characteristics, i.e., gender (binary; 1 = male), age (continuous), education (binary; 1 = tertiary education), annual household income in US dollar (continuous, log-transformed), and place of residence (binary; 1 = urban).
- Block 2 contained all variables measuring ideological views, i.e. right-leaning political orientation (5-point scale), conservative political orientation (5-point scale), religiosity (5-point scale), and social dominance orientation (continuous). Please also note that social dominance orientation was not measured in Malaysia, so the block-2 model did not rely on data from all 68 countries but only from 67 countries.
- Block 3 contained variables measuring attitudes to science, i.e., science-related populist attitudes (continuous), perceived personal benefit of science (continuous), willingness to be vulnerable to scientists (continuous), and trust in the scientific method (5-point scale). Please also note that willingness to be vulnerable to scientists was not measured in Mexico, so the block-3 model did not rely on data from 67 countries but only from 66 countries.
- Block 4 contained all country indicators, i.e., the GDP per capita, government expenditure on education in % of GDP, Gini Index, PISA science performance score, Academic Freedom Index, and prevalence of populist rhetoric in politics. Note that there was no PISA data available for some countries, so the block-4 models relied on data from only 51 countries (see section *Country indicators*).

Please note that we did not include the 1-item measure of confidence in scientists to act in the public interest as an independent variable in the models, which is a deviation from the preregistration (see section *Preregistration*). This was because the confidence measure had a very high correlation with the trust in scientists index (weighted Pearson's $r(69{,}502) = 0.707$ $p < .001$, $t = 301.71$, 95% CI 0.705 to 0.709), which is plausible given that confidence that scientists act in the best interests of the public is an essential aspect of the benevolence dimension of the trust index. Including the confidence measure in regression models explaining trust in scientists would thus have led to a substantial inflation of explanatory power, with Conditional R^2 being well above 0.70. We also found pronounced correlations of trust in scientists and the other block-3 independent variables (science-related populist attitudes, perceived personal benefit of science, willingness to be vulnerable, trust in the scientific method), but they were clearly less strong than correlations of the trust index and confidence measure, so we kept them in the models (see table S15).

Table S15. Weighted zero-order correlations of trust in scientists and the attitudes to science.

	Trust in scientists	Science-related populist attitudes	Confidence scientists act in public interest	Benefit of science	Willing-ness to be vulnerable	Trust in scientific method
Trust in scientists	1					
Science-related populist attitudes	-0.11***	1				
Confidence scientists act in public interest	0.71***	-0.14***	1			
Benefit of science	0.45***	-0.15***	0.40***	1		
Willingness to be vulnerable	0.46***	-0.12***	0.42***	0.39***	1	
Trust in scientific method	0.47***	-0.15***	0.48***	0.38***	0.47***	1

Note: Estimates are based on post-stratification weights accounting for unequal selection probabilities across countries, genders, age groups, and education levels. *** $p < .001$.

Table S16. Variance inflation factors for weighted linear multilevel regression model explaining trust in scientists.

Independent variable	VIF
Gender (male)	1.03
Age	1.03
Education (tertiary)	1.06
Income	1.04
Residence place (urban)	1.01
Political orientation (right)	1.46
Political orientation (conservative)	1.46
Religiosity	1.11
Social dominance orientation	1.18
Science-related populist attitudes	1.15
Perceived benefit of science	1.34
Willingness to be vulnerable to scientists	1.44
Trust in the scientific method	1.43
GDP per capita	2.23
Government expenditure on education	1.31
Gini index	1.37
PISA science literacy score	2.10
Academic freedom	1.60
Degree of populism in politics	1.46

Significance tests of regression estimates relied on the Satterthwaite method[89], which is one of several approaches to approximate the denominator degrees of freedom for *F* statistics or degrees of freedom for *t* statistics in multilevel models (besides the t-as-z, LRT, or Kenward-Roger approximations). For our models, which were estimated with restricted maximum likelihood (REML), the Satterthwaite[89] and Kenward-Roger methods[123] were found to produce the least biased estimates: Simulation studies showed that they have the lowest Type-1 error rates and outperform other approximation methods in small samples, with negligible differences between the two methods[124].We tested both the Satterthwaite and the Kenward-Roger method for our models and found that *t*, *df*, and *p* values did almost not differ: Minor deviations, if any, occurred only in the 3rd or 4th decimal place of *t* values, and in the 4th or 5th decimal place of *p* values (see replication materials). The Satterthwaite method seems to be very marginally more conservative in our case (i.e., *t* values were very marginally lower). We thus relied on the Satterthwaite method – also because it is considerably less computationally expensive.

Table S17. Variance inflation factors for weighted linear multilevel regression model explaining normative perceptions of science in society and policymaking.

Independent variable	VIF
Trust in scientists	1.06
Science-related populist attitudes	1.07
Gender (male)	1.02
Age	1.02
Education (tertiary)	1.04
Income	1.03
Residence place (urban)	1.01
Political orientation (right)	1.38
Political orientation (conservative)	1.37
Religiosity	1.07
Social dominance orientation	1.08

Analysis of factors explaining perceived and desired priorities of scientific research

For each model, the independent variables were included in four blocks. See table S21 for an overview of valid N across countries.

- Block 1 contained all variables measuring demographic characteristics, i.e., gender (binary; 1 = male), age (continuous), education (binary; 1 = tertiary education), annual household income in US dollar (continuous, log-transformed), and place of residence (binary; 1 = urban).
- Block 2 contained all variables measuring ideological views, i.e. right-leaning political orientation (5-point scale), conservative political orientation (5-point scale), religiosity (5-point scale), and social dominance orientation (continuous). Please also note that social dominance orientation was not measured in Malaysia, so the block-2 model did not rely on data from all 68 countries but only from 67 countries.
- Block 3 contained variables measuring attitudes to science, i.e., science-related populist attitudes (continuous), perceived personal benefit of science (continuous), willingness to be vulnerable to scientists (continuous), and trust in the scientific method (5-point scale). Please also note that willingness to be vulnerable to scientists was not measured in Mexico, so the block-3 model did not rely on data from 67 countries but only from 66 countries.
- Block 4 contained all country indicators, i.e., the GDP per capita, government expenditure on education in % of GDP, Gini Index, PISA science performance score, Academic Freedom Index, and prevalence of populist rhetoric in politics. Note that there was no PISA data available for some countries, so the block-4 models relied on data from only 51 countries (see section *Country indicators*).

Table S18. Variance inflation factors for weighted linear multilevel regression models explaining the discrepancy of desires that scientists should prioritize certain issues and perceptions that science addresses these issues.

	VIF			
	Model outcome: discrepancy score for…			
Independent variable	“improving public health”	“solving energy problems”	“reducing poverty”	“developing defence and military technology”
Trust in scientists	1.07	1.11	1.06	1.16
Science-related populist attitudes	1.04	1.04	1.02	1.11
Gender (male)	1.03	1.03	1.03	1.03
Age	1.02	1.02	1.02	1.02
Education (tertiary)	1.05	1.05	1.05	1.05
Income	1.03	1.03	1.03	1.03
Residence place (urban)	1.01	1.01	1.01	1.01
Political orientation (right)	1.41	1.41	1.40	1.41
Political orientation (conservative)	1.41	1.41	1.40	1.41
Religiosity	1.09	1.09	1.08	1.09
Social dominance orientation	1.11	1.11	1.10	1.11
Perceived benefit of science	1.12	1.13	1.11	1.14
Willingness to be vulnerable to scientists	1.17	1.19	1.17	1.20
Trust in the scientific method	1.17	1.19	1.17	1.20
GDP per capita	2.18	2.18	2.19	2.21
Government expenditure on education	1.28	1.28	1.29	1.30
Gini index	1.39	1.39	1.39	1.39
PISA science literacy score	2.11	2.11	2.10	2.11
Academic freedom	1.57	1.57	1.58	1.59
Degree of populism in politics	1.47	1.46	1.46	1.45

Table S19. Variance inflation factors for weighted linear multilevel regression model explaining trust in scientists with perceptions that science's efforts to tackle four goals (i.e., perceived priorities) exceed expectations for scientists to prioritize these goals (i.e., desired priorities)

Independent variable	VIF
Discrepancy score for “improving public health”	1.25
Discrepancy score for “solving energy problems”	1.29
Discrepancy score for “reducing poverty”	1.06
Discrepancy score for “developing defence and military technology”	1.09
Science-related populist attitudes	1.09
Gender (male)	1.03
Age	1.02
Education (tertiary)	1.05
Income	1.03
Residence place (urban)	1.01
Political orientation (right)	1.38
Political orientation (conservative)	1.38
Religiosity	1.07
Social dominance orientation	1.12
Perceived benefit of science	1.27
Willingness to be vulnerable to scientists	1.39
Trust in the scientific method	1.36
GDP per capita	2.23
Government expenditure on education	1.31
Gini index	1.38
PISA science literacy score	2.10
Academic freedom	1.60
Degree of populism in politics	1.46

Table S20. Valid N across countries for analyses testing means and standard errors across countries

	Weighted means and standard errors of…								
	Trust in scientists	Normative perceptions of scientists' engagement	Desired priority for developing defence and military technology	Competence dimension of trust in scientists	Integrity dimension of trust in scientists	Benevolence dimension of trust in scientists	Openness dimension of trust in scientists	Confidence that scientists act in public interests	Trust in the scientific method
Total	69,527	69,510	69,429	69,526	69,526	69,522	69,523	69,507	69,521
Albania	343	343	343	343	343	343	343	343	342
Argentina	495	495	493	495	495	495	495	495	495
Australia	3,523	3,522	3,522	3,523	3,523	3,523	3,523	3,521	3,523
Austria	1,035	1,034	1,035	1,035	1,035	1,035	1,035	1,035	1,035
Bangladesh	484	484	481	484	484	484	484	484	484
Belgium	2,035	2,035	2,035	2,035	2,035	2,034	2,034	2,032	2,035
Bolivia	493	493	486	493	493	493	493	492	493
Botswana	401	401	399	401	401	401	401	401	401
Brazil	1,214	1,214	1,207	1,214	1,214	1,214	1,214	1,214	1,214
Bulgaria	497	497	497	497	497	497	497	497	497
Cameroon	473	472	472	473	473	473	473	473	473
Canada	2,507	2,507	2,507	2,507	2,507	2,507	2,507	2,507	2,507
Chile	1,003	1,001	1,002	1,003	1,003	1,003	1,003	1,003	1,003
China	497	495	496	497	497	497	497	496	497
Colombia	498	499	495	498	498	498	498	496	499
Congo DR	389	389	389	389	389	389	389	389	389
Costa Rica	539	539	533	539	539	539	539	537	539
Cyprus	502	502	498	502	502	502	502	502	502
Czech Republic	495	495	495	495	495	495	495	495	495
Côte d'Ivoire	466	465	462	466	466	466	466	466	466
Denmark	1,208	1,208	1,206	1,208	1,208	1,208	1,208	1,208	1,208
Egypt	420	420	420	420	420	420	420	420	420
Ethiopia	363	362	361	363	363	363	363	363	363
Finland	998	998	997	998	998	997	998	998	998
France	2,006	2,003	2,007	2,006	2,006	2,006	2,006	2,006	2,005
Georgia	493	493	493	493	493	493	493	493	493
Germany	8,011	8,011	8,005	8,011	8,011	8,011	8,011	8,012	8,011
Ghana	474	474	474	474	474	474	474	474	474
Greece	1,436	1,436	1,434	1,436	1,436	1,436	1,436	1,436	1,436
Hong Kong	594	594	594	594	594	594	594	594	594
Hungary	498	498	497	498	498	498	498	497	497
India	473	473	473	473	473	473	473	473	473
Indonesia	2,056	2,056	2,055	2,056	2,056	2,056	2,056	2,056	2,055
Ireland	498	498	498	498	498	498	498	497	498
Israel	1,010	1,008	1,008	1,010	1,010	1,010	1,010	1,010	1,010
Italy	1,505	1,505	1,505	1,505	1,505	1,505	1,505	1,505	1,505
Japan	1,000	1,000	1,000	1,000	1,000	1,000	1,000	1,000	1,000
Kazakhstan	513	513	512	513	513	513	513	513	513

Kenya	456	456	456	456	456	456	456	456	456
Malaysia	985	985	983	985	985	984	984	985	985
Mexico	498	498	496	498	498	498	498	497	497
Morocco	375	374	373	375	375	375	375	375	375
Netherlands	1,410	1,409	1,403	1,410	1,410	1,410	1,410	1,409	1,409
New Zealand	2,009	2,009	2,006	2,008	2,008	2,008	2,009	2,009	2,009
Nicaragua	399	399	393	399	399	399	399	398	399
Nigeria	988	988	987	988	988	988	988	988	988
Norway	494	492	493	494	494	494	494	494	494
Peru	496	496	495	496	496	496	496	495	496
Philippines	545	545	545	545	545	545	545	545	545
Poland	3,002	3,002	3,000	3,002	3,002	3,002	3,002	3,002	3,002
Portugal	499	499	499	499	499	499	499	499	499
Romania	432	432	430	432	432	432	432	432	432
Russia	1,503	1,503	1,503	1,503	1,503	1,503	1,503	1,503	1,503
Serbia	499	499	498	499	499	499	499	498	498
Slovakia	530	530	530	530	530	530	530	530	530
Slovenia	501	500	501	501	501	501	501	501	501
South Africa	1,000	1,000	1,000	1,000	1,000	1,000	1,000	1,000	999
South Korea	500	500	500	500	500	500	500	500	500
Spain	1,009	1,009	1,008	1,009	1,009	1,009	1,009	1,009	1,009
Sweden	1,002	1,003	1,004	1,002	1,002	1,002	1,001	1,002	1,003
Switzerland	997	997	996	997	997	997	997	996	997
Taiwan	1,204	1,204	1,204	1,204	1,204	1,204	1,204	1,204	1,204
Türkiye	500	500	500	500	500	500	500	499	500
Uganda	387	387	385	387	387	387	387	387	387
Ukraine	1,008	1,008	1,004	1,008	1,008	1,008	1,008	1,007	1,008
United Kingdom	1,983	1,983	1,982	1,983	1,983	1,983	1,983	1,983	1,983
United States	2,559	2,559	2,559	2,559	2,559	2,559	2,559	2,559	2,559
Uruguay	312	312	310	312	312	311	311	312	312

Table S21. Valid N across countries for multilevel regressions

	Weighted multilevel regression model testing the association of…						
	Trust in scientists with demographic characteristics, attitudes, and country-level indicators (step 1)	Trust in scientists with demographic characteristics, attitudes, and country-level indicators (step 2)	Trust in scientists with demographic characteristics, attitudes, and country-level indicators (step 3)	Trust in scientists with demographic characteristics, attitudes, and country-level indicators (step 4)	Trust in scientists and normative perceptions of science in society	Confidence in scientists with demographic characteristics, attitudes, and country-level indicators (step 1)	Trust in the scientific method with demographic characteristics, attitudes, and country-level indicators (step 1)
Total	64,458	47,664	46,707	41,629	47,646	64,442	64,454
Albania	333	217	217	217	217	333	332
Argentina	478	294	294	294	294	478	478
Australia	3,392	2,772	2,768	2,768	2,771	3,390	3,392
Austria	972	797	797	797	796	972	972
Bangladesh	470	289	289	0	289	470	470
Belgium	1,779	1,273	1,273	1,273	1,273	1,778	1,779
Bolivia	481	303	302	0	302	480	481
Botswana	379	195	195	0	195	379	379
Brazil	1,204	890	889	889	889	1,204	1,204
Bulgaria	482	344	343	343	343	482	482
Cameroon	466	267	267	0	266	466	466
Canada	2,416	1,979	1,979	1,979	1,979	2,416	2,416
Chile	989	725	725	725	725	989	989
China	491	365	365	365	364	490	491
Colombia	460	316	316	316	316	458	461
Congo DR	379	300	300	0	300	379	379
Costa Rica	524	337	336	336	337	522	524
Cyprus	455	217	217	0	217	455	455
Czech Republic	467	377	376	376	376	467	467
Côte d'Ivoire	483	357	357	357	357	483	483
Denmark	1,096	784	784	784	784	1,096	1,096
Egypt	416	311	311	0	311	416	416
Ethiopia	358	188	188	0	188	358	358
Finland	490	363	363	363	363	490	490
France	1,907	1,220	1,219	1,219	1,220	1,907	1,906
Georgia	490	361	361	361	361	490	490
Germany	6,940	5,920	5,918	5,918	5,918	6,941	6,941
Ghana	464	263	263	0	263	464	464
Greece	1,290	992	991	991	991	1,290	1,290
Hong Kong	586	398	397	397	398	586	586
Hungary	473	325	324	324	325	472	472
India	469	401	401	0	401	469	469
Indonesia	2,014	1,546	1,545	1,545	1,546	2,014	2,013
Ireland	464	327	327	327	327	463	464
Israel	940	872	872	872	870	940	940
Italy	1,188	899	899	899	899	1,188	1,188
Japan	906	515	515	515	515	906	906
Kazakhstan	506	261	261	261	261	506	506

Kenya	447	355	355	0	355	447	447
Malaysia	972	0	0	0		972	972
Mexico	494	392	0	0	392	493	493
Morocco	367	171	171	171	171	367	367
Netherlands	1,257	879	336	336	878	1,256	1,256
New Zealand	1,887	1,515	1,513	1,513	1,515	1,887	1,887
Nicaragua	387	201	201	0	201	386	387
Nigeria	966	784	784	0	784	966	966
Norway	438	328	327	327	327	438	438
Peru	472	341	341	341	341	472	472
Philippines	540	434	434	434	434	540	540
Poland	2,861	2,184	2,184	2,184	2,184	2,861	2,861
Portugal	425	316	316	316	316	425	425
Romania	400	363	363	363	363	400	400
Russia	1,471	872	872	872	872	1,471	1,471
Serbia	476	308	307	307	308	476	475
Slovakia	502	362	362	362	362	502	502
Slovenia	479	332	332	332	331	479	479
South Africa	948	773	773	0	773	948	948
South Korea	476	404	404	404	404	476	476
Spain	943	773	773	773	773	943	943
Sweden	695	552	552	552	552	695	696
Switzerland	899	714	714	714	714	898	899
Taiwan	1,116	756	755	755	755	1,116	1,116
Türkiye	485	428	428	428	428	484	485
Uganda	368	232	232	0	232	368	368
Ukraine	992	397	397	397	397	991	992
United Kingdom	1,848	1,527	1,527	1,527	1,527	1,848	1,848
United States	2,466	2,147	2,147	2,147	2,147	2,466	2,466
Uruguay	284	264	263	263	263	284	284

Table S22. Overview IRB applications for ethics approval

Country of data collection	Surname lead collaborator(s)	Partner institution	Outcome / exception	IRB reference
Albania	Bajrami	University of Tirana	Harvard IRB approval sufficient	n/a
Argentina	Nobre	Universidade do Estado de Minas Gerais	Harvard IRB approval sufficient	n/a
Australia	Stanley	Australian National University	IRB approval granted	2022 506
Austria	Lamm	University of Vienna	IRB approval granted	EK Nr: 000412
Bangladesh	Islam	Jahangirnagar University	Harvard IRB approval sufficient	n/a
Belgium	De Peuter	KU Leuven	IRB approval granted	G-2022-5777-R2(MAR)
Bolivia	Cologna	Harvard University	Harvard IRB approval sufficient	n/a
Botswana	Kotcher	George Mason University	Exempt from full IRB review	IRBNet #2008429
	Bati	Health Research and Development Division, Ministry of Health, Botswana	IRB approval granted	HPRD: 6/14/1
Brazil	Azevedo	Friedrich Schiller University Jena	Harvard IRB approval sufficient	n/a
Bulgaria	Lamm	University of Vienna	IRB approval granted	EK Nr: 000412
Cameroon	Ntui-Njock	University of Buea	IRB approval granted	2196-11
Canada	Palmer-Hague	Trinity Western University	Harvard IRB approval sufficient	n/a
	Breeden	Pomona College	Exempt from full IRB review	#23-01-04
	Goddard	University of Alberta	IRB approval granted	Pro00128481
Chile	Garrido-Vásquez	Universidad de Concepción	IRB approval granted	CEBB 1372-2022
China	Xia	Tongji University	Harvard IRB approval sufficient	n/a
Colombia	Douglas	University of Kent	IRB approval granted	8016
Congo DR	Späth	Université Officielle de Bukavu	IRB approval granted	UOB/FSSPA/CFE/05/2023
Costa Rica	Altenmüller	LMU Munich	No IRB application required as per national/institutional regulations	n/a
Côte d'Ivoire	Toko	Mohammed VI Polytechnic University	IRB approval granted	n/a
Cyprus	Kyza	Cyprus University of Technology	Harvard IRB approval sufficient	n/a

Czech Republic	Klabíková Rábová	Charles University	Harvard IRB approval sufficient	n/a
Denmark	Fuglsang	Aarhus University	Harvard IRB approval sufficient	n/a
Egypt	Aldoh	University of Amsterdam	IRB approval granted	FMG-1067
Ethiopia	Debnath	University of Cambridge	Exempt from full IRB review	5051.145
Finland	Koivula	University of Turku	No IRB application required as per national/institutional regulations	n/a
France	Bret	Université Paris Nanterre (CER-PN)	IRB approval granted	n/a
Georgia	Pisareva	KIMEP University	Harvard IRB approval sufficient	n/a
Germany	Von Bubnoff	Rhine-Waal University	No IRB application required as per national/institutional regulations	n/a
	Kojan	University of Lübeck	IRB approval granted	2022-632
	Dries	Harding Center for Risk Literacy, University of Potsdam	Harvard IRB approval sufficient	n/a
	Genschow	Leuphana University Lüneburg	Harvard IRB approval sufficient	n/a
	Fischer	Leibniz Institut für Wissensmedien	Declared exempt from full IRB review	n/a
	Metag	University of Münster	Harvard IRB approval sufficient	n/a
	Rödder	Universität Hamburg	Harvard IRB approval sufficient	n/a
	Dohle	University of Bonn	No IRB application required as per national/institutional regulations	n/a
Ghana	Feuerriegel	LMU Munich	Harvard IRB approval sufficient	n/a
Greece	Gkinopoulos	University of Warsaw	IRB approval granted	n/a
	Kuppens	University of Groningen	Declared exempt from full IRB review	PSY-2223-S-0167
Hong Kong	Huang	City University of Hong Kong	IRB approval granted	HU-STA-00000276
Hungary	Szaszi	Eötvös Loránd University	IRB approval granted	2022/472
India	Debnath	University of Cambridge	Declared exempt from full IRB review	5051.145
Indonesia	Gordon	Sunan Kalijaga State Islamic University	IRB approval granted	B-292a/Un.02/L3/TL/01/2023
	Facciani	University of Notre Dame	No IRB application required as per	n/a

			national/institutional regulations	
Ireland	Roche	Trinity College Dublin	IRB approval granted	n/a
Israel	Herziger	Technion – Israel Institute of Technology	IRB approval granted	135260
Italy	Bertsou	University of St. Gallen	IRB approval granted	HSG-EC-020221101
Japan	Tanaka	Waseda University	IRB approval granted	#2022-446
Kazakhstan	Ibadildin	KIMEP University	IRB approval granted	n/a
Kenya	Amollo	National Commission for Science, Innovation and Technology	IRB approval granted	EUISERC/APP/228/ 2023
		Egerton University	IRB approval granted	EUISERC/APP/244/ 2023
Malaysia	Facciani	University of Notre Dame	No IRB application required as per national/institutional regulations	n/a
Mexico	Hernández-Mondragón	Center for Research and Advanced Studies of the National Polytechnic Institute	Harvard IRB approval sufficient	n/a
Morocco	Jeddi	Mohammed VI Polytechnic University	IRB approval granted	n/a
Netherlands	Kuppens	University of Groningen	Declared exempt from full IRB review	PSY-2223-S-0167
New Zealand	Stanley	Australian National University	IRB approval granted	2022 506
	Milfont	University of Waikato	IRB approval granted	FS2022-64
Nicaragua	Röer	Witten/Herdecke University	Harvard IRB approval sufficient	n/a
Nigeria	Von Bubnoff	Rhine-Waal University	Declared exempt from full IRB review	n/a
Norway	Kristiansen	University of Bergen	Harvard IRB approval sufficient	n/a
Peru	Monge	Ernest Manheim Public Opinion Laboratory	IRB approval granted	D20230104
Philippines	Cases	College of Social Sciences and Philosophy, University of the Philippines Diliman	IRB approval granted	CSSPERB-2023-005
Poland	Hensel	University of Warsaw	IRB approval granted	n/a
	Czarnek	Jagiellonian University	IRB approval granted	KE/1_2023
Portugal	Santos	Institute of Environmental Health Lisbon of the School of Medicine	Harvard IRB approval sufficient	n/a
Romania	Etienne	VU University Amsterdam	Declared exempt from full IRB review	2023-1-30-434
Russia	Grigoryev	HSE University	IRB approval granted	01/10.2022
Serbia	Lazić	University of Belgrade	IRB approval granted	#2022-76

Slovakia	Buchel	Institute for Sociology of the Slovak Academy of Sciences	IRB approval granted	IRB22-1046
Slovenia	Muršič	University of Ljubljana	Harvard IRB approval sufficient	n/a
South Africa	Joubert	Stellenbosch University	IRB approval granted	SU - SEBR - 26177
South Korea	Scoggins	KIMEP University	Harvard IRB approval sufficient	n/a
Spain	Cabrera Alvarez	University of Essex	Harvard IRB approval sufficient	n/a
Sweden	Parnamets	Karolinska Institutet	No IRB application required as per national/institutional regulations	n/a
Switzerland	Freundt	University of Fribourg	Harvard IRB approval sufficient	n/a
Taiwan	Rauchfleisch	National Taiwan University	IRB approval granted	202312HS006
Türkiye	Çoksan	Erzurum Technical University	Harvard IRB approval sufficient	n/a
Uganda	Namutebi	Makerere University College of Humanities and Social Sciences	IRB approval granted	MAKSSREC 05.2023.680
Ukraine	Ploszaj	University of Warsaw	Harvard IRB approval sufficient	n/a
United Kingdom	Bhatiya	University of Birmingham	Harvard IRB approval sufficient	n/a
	Debnath	University of Cambridge	Declared exempt from full IRB review	5051.145
	Douglas	University of Kent	IRB approval granted	8016
	Alabrese	University of Bath	Harvard IRB approval sufficient	n/a
	Ulug	University of Sussex	Harvard IRB approval sufficient	n/a
USA	Motta	Boston University	Declared exempt from full IRB review	H-43232
	Vilares	University of Minnesota	IRB approval granted	STUDY00005811
Uruguay	Etienne	VU University Amsterdam	Declared exempt from full IRB review	2023-1-30-546

Additional References

90. van der Meer, T. & Hakhverdian, A. Political Trust as the Evaluation of Process and Performance: A Cross-National Study of 42 European Countries. *Polit. Stud.* **65**, 81–102 (2017).
91. O'Brien, T. L. & Noy, S. Cultural Authority in Comparative Context: A Multilevel Analysis of Trust in Science and Religion. *J. Sci. Study Relig.* **57**, 495–513 (2018).
92. Vallejo, B. M. & Ong, R. A. C. Policy responses and government science advice for the COVID 19 pandemic in the Philippines: January to April 2020. *Prog. Disaster Sci.* **7**, 100115 (2020).
93. Tony Blair Institute for Global Change. Pandemic Populism: An Analysis of Populist Leaders' Responses to Covid-19. https://www.institute.global/insights/public-services/pandemic-populism-analysis-populist-leaders-responses-covid-19 (2020).
94. Buštíková, L. & Baboš, P. Best in Covid: Populists in the Time of Pandemic. *Polit. Gov.* **8**, 496–508 (2020).
95. Allen, M. S., Iliescu, D. & Greiff, S. Single Item Measures in Psychological Science. *Eur. J. Psychol. Assess.* **38**, 1–5 (2022).
96. Tavakol, M. & Dennick, R. Making sense of Cronbach's alpha. *Int J Med Educ* **2**, 53–55 (2011).
97. Streiner, D. L. Starting at the Beginning: An Introduction to Coefficient Alpha and Internal Consistency. *J. Pers. Assess.* **80**, 99–103 (2003).
98. Wuttke, A., Schimpf, C. & Schoen, H. When the Whole Is Greater than the Sum of Its Parts: On the Conceptualization and Measurement of Populist Attitudes and Other Multidimensional Constructs. *Am. Polit. Sci. Rev.* **114**, 356–374 (2020).
99. Mede, N. G., Schäfer, M. S., Metag, J. & Klinger, K. Who supports science-related populism? A nationally representative survey on the prevalence and explanatory factors of populist attitudes toward science in Switzerland. *PLOS ONE* **17**, e0271204 (2022).
100. Mede, N. G., Schäfer, M. S. & Metag, J. Cognitio populi – Vox populi: Implications of science-related populism for communication behavior. *Communications* (2023) doi:10.1515/commun-2022-0059.
101. World Bank & OECD. GDP per capita (current US$). (2022).

102. Directorate-General of Budget, Accounting and Statistics, Republic of China. Statistical Yearbook of the Republic of China 2021: Per capita income and consumption. (2022).
103. UNESCO Institute for Statistics. Government expenditure on education, total (% of GDP). (2022).
104. Directorate-General of Budget, Accounting and Statistics, Republic of China. Statistical Yearbook of the Republic of China 2021: Summary of R&D expenditure. (2022).
105. World Bank. Gini index. Poverty and Inequality Platform. (2022).
106. World Economics. Gini Coefficient. (2022).
107. PISA. Science performance (PISA): Boys / Girls, Mean score, 2018 or latest available. (2022).
108. OECD. Pisa Database. (2022).
109. V-Dem. Country-Year: V-Dem Core Dataset v13. (2023).
110. Spannagel, J. & Kinzelbach, K. The Academic Freedom Index and Its indicators: Introduction to new global time-series V-Dem data. *Qual. Quant.* (2022) doi:10.1007/s11135-022-01544-0.
111. Norris, P. & Inglehart, R. *Cultural Backlash: Trump, Brexit, and Authoritarian Populism.* (Cambridge University Press, 2019).
112. Devinney, T. M. & Hartwell, C. A. Varieties of populism. *Glob. Strategy J.* **10**, 32–66 (2020).
113. Guan, T. & Yang, Y. Rights-oriented or responsibility-oriented? Two subtypes of populism in contemporary China. *Int. Polit. Sci. Rev.* **42**, 672–689 (2021).
114. Zhang, C. Right-wing populism with Chinese characteristics? Identity, otherness and global imaginaries in debating world politics online. *Eur. J. Int. Relat.* **26**, 88–115 (2020).
115. Aslanidis, P. Populism and Social Movements. in *The Oxford Handbook of Populism* (eds. Kaltwasser, C. R., Taggart, P., Espejo, P. O. & Ostiguy, P.) 0 (Oxford University Press, 2017). doi:10.1093/oxfordhb/9780198803560.013.23.
116. Lam-Knott, S. "Populism" in Hong Kong's contemporary politics. *The Immanent Frame.* https://tif.ssrc.org/2020/02/26/populism-in-hong-kongs-contemporary-politics/ (2020).
117. Ng, H.-Y. & Kennedy, K. J. Localist groups and populist radical regionalism in Hong Kong. *China: An International Journal* **17**, 111–134 (2019).

118. Tang, G. Media populism in post-handover Hong Kong: an investigation of media framing of public finance. *Chin. J. Commun.* **10**, 433–449 (2017).
119. Claessens, S., Kyritsis, T. & Atkinson, Q. D. Cross-national analyses require additional controls to account for the non-independence of nations. *Nat. Commun.* **14**, 5776 (2023).
120. Paccagnella, O. Centering or Not Centering in Multilevel Models? The Role of the Group Mean and the Assessment of Group Effects. *Eval. Rev.* **30**, 66–85 (2006).
121. Enders, C. K. & Tofighi, D. Centering predictor variables in cross-sectional multilevel models: A new look at an old issue. *Psychol. Methods* **12**, 121–138 (2007).
122. Yaremych, H. E., Preacher, K. J. & Hedeker, D. Centering categorical predictors in multilevel models: Best practices and interpretation. *Psychol. Methods* **28**, 613–630 (2023).
123. Kenward, M. G. & Roger, J. H. Small Sample Inference for Fixed Effects from Restricted Maximum Likelihood. *Biometrics* **53**, 983–997 (1997).
124. Luke, S. G. Evaluating significance in linear mixed-effects models in R. *Behav. Res. Methods* **49**, 1494–1502 (2017).
