# Supplementary material for: Trust in scientists and their role in society across 68 countries
Source: Nat Hum Behav. 2025 Jan 20;9(4):713–30. doi: 10.1038/s41562-024-02090-5 (PMC7617525; doi:10.1038/s41562-024-02090-5)
Supplement: Supplementary file 2 — Reporting Summary [file 41562_2024_2090_MOESM2_ESM.pdf]

nature portfolio

Corresponding author(s): Viktoria Cologna

Last updated by author(s): Oct 24, 2024

Reporting Summary

Nature Portfolio wishes to improve the reproducibility of the work that we publish. This form provides structure for consistency and transparency in reporting. For further information on Nature Portfolio policies, see our Editorial Policies and the Editorial Policy Checklist.

Statistics

For all statistical analyses, confirm that the following items are present in the figure legend, table legend, main text, or Methods section.

n/a	Confirmed	
☐	☒	The exact sample size (*n*) for each experimental group/condition, given as a discrete number and unit of measurement
☐	☒	A statement on whether measurements were taken from distinct samples or whether the same sample was measured repeatedly
☐	☒	The statistical test(s) used AND whether they are one- or two-sided<br>*Only common tests should be described solely by name; describe more complex techniques in the Methods section.*
☐	☒	A description of all covariates tested
☐	☒	A description of any assumptions or corrections, such as tests of normality and adjustment for multiple comparisons
☐	☒	A full description of the statistical parameters including central tendency (e.g. means) or other basic estimates (e.g. regression coefficient) AND variation (e.g. standard deviation) or associated estimates of uncertainty (e.g. confidence intervals)
☐	☒	For null hypothesis testing, the test statistic (e.g. *F*, *t*, *r*) with confidence intervals, effect sizes, degrees of freedom and *P* value noted<br>*Give P values as exact values whenever suitable.*
☒	☐	For Bayesian analysis, information on the choice of priors and Markov chain Monte Carlo settings
☐	☒	For hierarchical and complex designs, identification of the appropriate level for tests and full reporting of outcomes
☐	☒	Estimates of effect sizes (e.g. Cohen's *d*, Pearson's *r*), indicating how they were calculated

Our web collection on statistics for biologists contains articles on many of the points above.

Software and code

Policy information about availability of computer code

Data collection	Qualtrics software, versions 11-2022 throughout 08-2023. Qualtrics, Provo, UT, USA.
Data analysis	R v4.4.1 (2024-06-14 ucrt), platform: x86_64-w64-mingw32/x64 under Windows 11 x64 (build 22631). Name and version of packages:<br>broom 1.0.7<br>broom.mixed 0.2.9.5<br>car 3.1-3<br>carData 3.0-5<br>corrplot 0.94<br>countrycode 1.6.0<br>data.table 1.16.0<br>datawizard 0.12.3<br>dplyr 1.1.4<br>forcats 1.0.0<br>ggalt 0.4.0<br>ggbump 0.1.0<br>ggdag 0.2.13<br>ggflags 0.0.4<br>ggplot2 3.5.1<br>ggpubr 0.6.0<br>GPArotation 2024.3-1<br>gridExtra 2.3

haven 2.5.4
Hmisc 5.1-3
insight 0.20.4
jtools 2.3.0
lavaan 0.6-19
lme4 1.1-35.5
lmerTest 3.1-3
lubridate 1.9.3
magrittr 2.0.3
maps 3.4.2
marginaleffects 0.22.0
Matrix 1.7-0
moments 0.14.1
MVN 5.9
optimx 2023-10.21
performance 0.12.3
psych 2.4.6.26
purrr 1.0.2
quantmod 0.4.26
readr 2.1.5
readxl 1.4.3
remotes 2.5.0
reshape2 1.4.4
rlang 1.1.4
rsample 1.2.1
rstudioapi 0.16.0
scales 1.3.0
sjlabelled 1.2.0
sjPlot 2.8.16
srvyr 1.3.0
stringr 1.5.1
survey 4.4-2
survival 3.6-4
tibble 3.2.1
tidyr 1.3.1
tidyverse 2.0.0
TTR 0.24.4
viridis 0.6.5
viridisLite 0.4.2
weights 1.0.4
xts 0.14.0
zoo 1.8-12

For manuscripts utilizing custom algorithms or software that are central to the research but not yet described in published literature, software must be made available to editors and reviewers. We strongly encourage code deposition in a community repository (e.g. GitHub). See the Nature Portfolio guidelines for submitting code & software for further information.

Data

Policy information about availability of data

All manuscripts must include a data availability statement. This statement should provide the following information, where applicable:

- Accession codes, unique identifiers, or web links for publicly available datasets
- A description of any restrictions on data availability
- For clinical datasets or third party data, please ensure that the statement adheres to our policy

The dataset underlying this article is publicly available at: https://doi.org/10.17605/OSF.IO/5C3QD Mede46 provide detailed information on the dataset, including data collection and pre-processing.

Research involving human participants, their data, or biological material

Policy information about studies with human participants or human data. See also policy information about sex, gender (identity/presentation), and sexual orientation and race, ethnicity and racism.

Reporting on sex and gender	Gender was determined based on self-reporting. Participants were also given the option to select "Prefer not to say".
Reporting on race, ethnicity, or other socially relevant groupings	Race and ethnicity were not assessed in this study. All assessed socio-demographic variables were determined based on self-reporting.
Population characteristics	Participant's gender, age, education, income, place of residence, political orientation, and religiosity were determined based on self-reporting.

Recruitment	Data were collected in on line surveys that used quotas for age (five bins: 20% 18-29 years, 20% 30-39 years, 20% 40-49 I years, 20% 50-59 years, 20% 60 years and older) and gender (two bins: 50% male, 50% female). Participants had to be 18 years of age or older and provide informed consent to participate in the study. The surveys were programmed in Qualtrics. Participants that completed the survey were remunerated according to the market research company's local rates. All data was collected via on line surveys, except for the Democratic Republic of Congo, where participants were interviewed in face/ to-face interviews and responses recorded in Qualtrics by the interviewers. Respondents were recruited from online panels of the market research companies Bilendi & respondi, MSi, Prolific, 2muse, and Kieskompas. They received vouchers/credit points for completing the full survey, which they could redeem and/or transfer into money.
Ethics oversight	Harvard University-Area Committee on the Use of Human Subjects (protocol# IRB22-1046).

Note that full information on the approval of the study protocol must also be provided in the manuscript.

Field-specific reporting

Please select the one below that is the best fit for your research. If you are not sure, read the appropriate sections before making your selection.

☐ Life sciences ☒ Behavioural & social sciences ☐ Ecological, evolutionary & environmental sciences

For a reference copy of the document with all sections, see nature.com/documents/nr-reporting-summary-flat.pdf

Behavioural & social sciences study design

All studies must disclose on these points even when the disclosure is negative.

Study description	This study uses quantitative data. They were collected in a global, pre-tested, pre-registered, cross-sectional on line survey (N = 71,922 participants in k = 68 countries) between November 2022 and August 2023 as part of the TISP Many Labs project ("Trust in Science and Science-Related Populism"). TISP is an international, multidisciplinary consortium of 241 researchers from 171 institutions across all continents.
Research sample	Researchers conducted on line surveys within 88 post-hoe weighted quota samples in 68 countries, using the same questionnaire translated into 37 languages. Data were collected in surveys that used quotas for age (five bins: 20% 18-29 years, 20% 30-39 years, 20% 40-49 years, 20% 50-59 years, 20% 60 years and older) and gender (two bins: 50% male, 50% female). Participants had to be 18 years of age or older and provide informed consent to participate in the study. Therefore the samples are not representative. We were interested in public attitudes towards scientists and their role in society and policy-making, hence we used samples of the general population.
Sampling strategy	To determine our minimum target sample size, we ran simulation-based power analyses using the R package simr (vl.0.7) which is designed to conduct power analyses for generalized linear mixed models such as those we used in the main study (for detailed information see SI). Based on these analyses we determined a minimum target sample size of 7,500, with n = 500 in k = 15 countries to detect fixed effects of trust in scientists and science-related populists as small as b = 0.10 and b = 0.05, respectively. Our final sample of 71,922 individuals with k = 68 countries is thus by far big enough to detect even smaller effects of trust in scientists and science-related populist attitudes. We used non-probability quota sampling, with balanced quotas for age (five bins: 20% 18-29 years, 20% 30-39 years, 20% 40-49 years, 20% 50-59 years, 20% 60 years and older) and gender (two bins: 50% male, 50% female).
Data collection	Data were collected in surveys that used quotas for age (five bins: 20% 18-29 years, 20% 30-39 years, 20% 40-49 years, 20% 50-59 years, 20% 60 years and older) and gender (two bins: 50% male, 50% female). Participants had to be 18 years of age or older and provide informed consent to participate in the study. The surveys were programmed in Qualtrics. Participants that completed the survey were remunerated according to the market research company's local rates. All data was collected via on line surveys, except for the Democratic Republic of Congo, where participants were interviewed in face-to-face interviews and responses recorded in Qualtrics by the interviewers. Participants were recruited with the market research company Bilendi & Respondi, except for most African countries, where data was collected with the market research company MSi.<br>The researchers were not blinded to the research questions and hypotheses.
Timing	Data were collected between November 2022 and August 2023.
Data exclusions	We excluded all respondents who did not complete the survey, because they cancelled participation during the survey, were filtered as their gender x age quota was already full, or because they did not pass one of the two attention checks. The TISP dataset contains complete records of N = 71,922 participants from 88 samples across k = 68 countries. Overall, we collected a total of N = 72,135 complete responses but had to delete 213 records from duplicate respondents.
Non-participation	We are not aware of how many participants that were invited to participate by the market research company declined participation.
Randomization	Participants were not allocated into experimental groups.

Reporting for specific materials, systems and methods

We require information from authors about some types of materials, experimental systems and methods used in many studies. Here, indicate whether each material, system or method listed is relevant to your study. If you are not sure if a list item applies to your research, read the appropriate section before selecting a response.

Materials & experimental systems

n/a	Involved in the study
☒	☐ Antibodies
☒	☐ Eukaryotic cell lines
☒	☐ Palaeontology and archaeology
☒	☐ Animals and other organisms
☒	☐ Clinical data
☒	☐ Dual use research of concern
☒	☐ Plants

Methods

n/a	Involved in the study
☒	☐ ChIP-seq
☒	☐ Flow cytometry
☒	☐ MRI-based neuroimaging

Plants

Seed stocks	*Report on the source of all seed stocks or other plant material used. If applicable, state the seed stock centre and catalogue number. If plant specimens were collected from the field, describe the collection location, date and sampling procedures.*
Novel plant genotypes	*Describe the methods by which all novel plant genotypes were produced. This includes those generated by transgenic approaches, gene editing, chemical/radiation-based mutagenesis and hybridization. For transgenic lines, describe the transformation method, the number of independent lines analyzed and the generation upon which experiments were performed. For gene-edited lines, describe the editor used, the endogenous sequence targeted for editing, the targeting guide RNA sequence (if applicable) and how the editor was applied.*
Authentication	*Describe any authentication procedures for each seed stock used or novel genotype generated. Describe any experiments used to assess the effect of a mutation and, where applicable, how potential secondary effects (e.g. second site T-DNA insertions, mosiacism, off-target gene editing) were examined.*
